# Modulation by DREADD reveals the therapeutic effect of human iPSC-derived neuronal activity on functional recovery after spinal cord injury

**DOI:** 10.1016/j.stemcr.2021.12.005

**Published:** 2022-01-11

**Authors:** Takahiro Kitagawa, Narihito Nagoshi, Yasuhiro Kamata, Momotaro Kawai, Kentaro Ago, Keita Kajikawa, Reo Shibata, Yuta Sato, Kent Imaizumi, Tomoko Shindo, Munehisa Shinozaki, Jun Kohyama, Shinsuke Shibata, Morio Matsumoto, Masaya Nakamura, Hideyuki Okano

**Affiliations:** 1 Department of Orthopaedic Surgery, Keio University School of Medicine, 35 Shinanomachi, Shinjuku-ku, Tokyo 160-8582, Japan; 2 Department of Physiology, Keio University School of Medicine, 35 Shinanomachi, Shinjuku-ku, Tokyo 160-8582, Japan; 3 Graduate School of Science and Technology, Keio University, 3-14-1 Hiyoshi, Kohoku-ku, Yokohama City, Kanagawa 223-8522, Japan; 4 Laboratory for Marmoset Neural Architecture, RIKEN Center for Brain Science, 2-1 Hirosawa, Wako City, Saitama 351-0198, Japan; 5 Electron Microscope Laboratory, Keio University School of Medicine, 35 Shinanomachi, Shinjuku-ku, Tokyo 160-8582, Japan; 6 Division of Microscopic Anatomy, Graduate School of Medical and Dental Sciences, Niigata University, 1-757 Asahimachi-dori, Chuo-ku, Niigata City, Niigata 951-8510, Japan

**Keywords:** spinal cord injury, cell transplantation, human iPS cell, DREADD

## Abstract

Transplantation of neural stem/progenitor cells (NS/PCs) derived from human induced pluripotent stem cells (hiPSCs) is considered to be a promising therapy for spinal cord injury (SCI) and will soon be translated to the clinical phase. However, how grafted neuronal activity influences functional recovery has not been fully elucidated. Here, we show the locomotor functional changes caused by inhibiting the neuronal activity of grafted cells using a designer receptor exclusively activated by designer drugs (DREADD). *In vitro* analyses of inhibitory DREADD (hM4Di)-expressing cells demonstrated the precise inhibition of neuronal activity via administration of clozapine N-oxide. This inhibition led to a significant decrease in locomotor function in SCI mice with cell transplantation, which was exclusively observed following the maturation of grafted neurons. Furthermore, trans-synaptic tracing revealed the integration of graft neurons into the host motor circuitry. These results highlight the significance of engrafting functionally competent neurons by hiPSC-NS/PC transplantation for sufficient recovery from SCI.

## Highlights

• The neuronal activity of hM4Di-NS/PCs was controlled by CNO administration
• Inhibiting the neuronal activity of grafted NS/PCs led to functional decline
• Grafted neurons derived from hiPSC-NS/PCs integrated into host motor circuits

## Teaser

This study by Okano, Nagoshi, and colleagues demonstrated decreased locomotor function in hiPSC-NS/PC-transplanted SCI mice induced by inhibition of graft-derived neuronal activity using a DREADD. Locomotor changes caused by the graft neuronal activity inhibition were observed exclusively in the chronic phase. These results suggested the significance of engrafting functionally mature neurons via hiPSC-NS/PC transplantation for behavioral improvement following SCI.

## Introduction

Stem cell-based approaches have been reported to be an effective therapy for spinal cord injury (SCI) (Cummings et al., 2005; Kobayashi et al., 2012; Nori et al., 2011). Previous reports from our group and others have demonstrated the efficacy of transplanting neural stem/progenitor cells (NS/PCs) derived from human induced pluripotent stem cells (hiPSCs), which are considered a promising cell source with limited ethical concerns (Fujimoto et al., 2012; Kobayashi et al., 2012; Nori et al., 2011; Okubo et al., 2016). Several beneficial factors of NS/PC transplantation have been proposed, including axonal regeneration with synaptic formation, remyelination, and neuroprotection by trophic factors (Assinck et al., 2017; Nori et al., 2017). Among these mechanisms, axonal regeneration is of great significance due to the characteristics of NS/PCs, which provide neuronal cells that integrate into host tissue and reconnect neuronal networks (Abematsu et al., 2010; Bonner et al., 2011; Bonner and Steward, 2015; Lu et al., 2012).

A number of previous studies emphasized the synaptic connection between graft and host neurons and implied the necessity of integrating graft neurons into host neuronal circuits (Ceto et al., 2020; Kadoya et al., 2016; Lu et al., 2012, 2019). For instance, our previous study transplanting neurogenic NS/PCs led to a further functional improvement of host animals, which suggested the potency of robust neuronal differentiation (Okubo et al., 2016). However, our studies and others advocated the importance of neuronal connectivity mainly by histological analyses, and there was a lack of direct proof displaying the relationship between neuronal activity in grafted neurons and locomotor outcomes. Definitive evidence of the therapeutic mechanism mediated by integrated graft-derived neurons is crucial to promote hiPSC-NS/PC transplantation therapy for SCI, which is now on the road toward clinical trials (Nagoshi et al., 2020; Tsuji et al., 2019).

Several approaches can be utilized to demonstrate how the transplanted cells actually contribute to the improvement of motor function. Administration of diphtheria toxin is frequently used to ablate engrafted human-derived cells (Abematsu et al., 2010; Cummings et al., 2005; Fujimoto et al., 2012). However, this methodology eliminates all the grafted cells and cannot assess the function of engrafted neurons. To compensate for this limitation, designer receptors exclusively activated by designer drugs (DREADD) have attracted attention for their ability to specifically control neuronal activity (Nichols and Roth, 2009; Roth, 2016). This is one of the chemogenetically engineered proteins that permit control of G protein signaling by administering the ligand clozapine N-oxide (CNO) and manipulating neuronal activity (Aldrin-Kirk et al., 2016; Nichols and Roth, 2009; Roth, 2016). Several studies have conducted cell transplantation research using the DREADD system for central nervous system disorders (Aldrin-Kirk et al., 2016; Chen et al., 2016; Zou et al., 2016). In a model of Parkinson's disease, functional decline in host animals induced by the selective inhibition of transplanted dopamine neurons indicated the therapeutic efficacy of cell replacement therapy (Chen et al., 2016). In a previous study on SCI, one study transplanted neuroepithelial stem cells derived from embryonic postmortem specimens and assessed the impact of graft neuronal inhibition on locomotor function (Dell'Anno et al., 2018). However, this report had the following limitations that prohibited complete elucidation of the role of graft-derived neuronal function: limited functional recovery by the cell replacement, inadequate control animals that did not fully eliminate the possibility of off-target effects by the DREADD system, and no confirmation that the grafted neurons were connected to the host motor circuits. Most importantly, no previous study has revealed the direct contribution of neuronal activity in grafted hiPSC-NS/PCs using an SCI animal model.

The present study aimed to evaluate the association of graft neuronal activity and host locomotor function in hiPSC-derived NS/PC transplantation by controlling the neuronal activity using the inhibitory DREADD receptor hM4Di (Nichols and Roth, 2009). Adding a technique of trans-synaptic tracing, the current study rigorously clarified the mechanisms of cell transplantation therapy in SCI, especially focusing on the neuronal function.

## Results

### The activity of hM4Di-expressing neurons derived from hiPSC-NS/PCs was inhibited by administration of CNO

**Figure 1:**
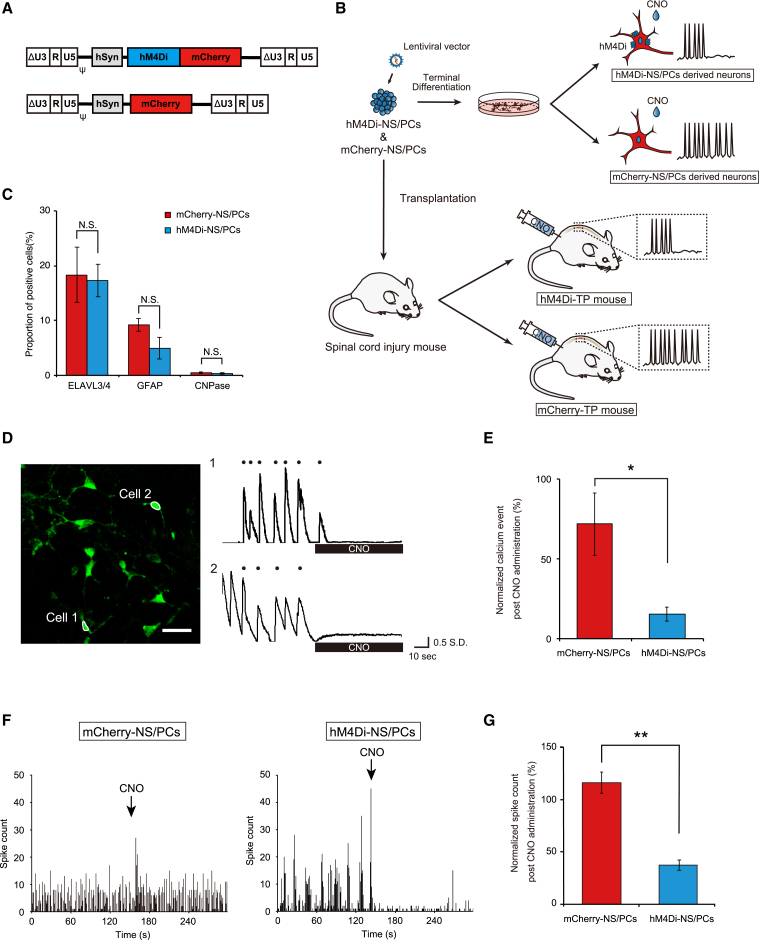
Establishment of hM4Di-expressing NS/PCs; differential potential and inhibitory functional assessments *in vitro* (A) Schematic of the integrated proviral form of the lentiviral vectors. Top: CSIV-hSyn-hM4Di-mCherry. Bottom: CSIV-hSyn-mCherry. (B) Schematic of the experiments. (C) Percentage of cell-type-specific marker cells in differentiated cells of mCherry-NS/PCs and hM4Di-NS/PCs (mCherry-NS/PCs, n = 4 wells/2 independent experiments; hM4Di-NS/PCs, n = 4 wells/2 independent experiments). (D) Representative calcium transients of neurons differentiated from hM4Di-NS/PCs. Left: an image of neurons expressing jGCaMP7f in a single field of view. Scale bar, 30 μm. Right: time traces of representative cells highlighted with white outlines in the left image (the black dot indicates the calcium event). (E) Average of normalized calcium events after CNO administration for neurons differentiated from mCherry-NS/PCs and hM4Di-NS/PCs (mCherry-NS/PCs, n = 15 cells/3 independent experiments; hM4Di-NS/PCs, n = 18 cells/3 independent experiments). (F) Evaluation of neural activity by multi-electrode array assay. Representative time traces of the spike count by CNO administration (left, mCherry-NS/PCs; right, hM4Di-NS/PCs). (G) Average normalized spike counts after CNO administration of neurons differentiated from mCherry-NS/PCs and hM4Di-NS/PCs (mCherry-NS/PCs, n = 10 wells/2 independent experiments; hM4Di-NS/PCs, n = 10 wells/2 independent experiments). ^∗^p < 0.05, ^∗∗^p < 0.01, and N.S., not significant according to the Mann-Whitney U test (C, E, and G). The data are presented as the mean ± SEM.

We used lentiviral vectors to label NS/PCs, which derived neurons expressing hM4Di fused with mCherry (hM4Di-NS/PCs) and neurons expressing only mCherry as a negative control (mCherry-NS/PCs) (Figures 1A and 1B). The expression of these genes was controlled by the human Synapsin (hSyn) promoter (Egashira et al., 2018).

The differential potentials of the two cell lines were not different as determined by immunocytochemical (ICC) analysis, which yielded the following results: embryonic lethal abnormal vision-like protein 3/4 (ELAVL3/4)^+^ neurons (17.3% ± 3.0% versus 18.4% ± 5.0%, p = 1.000), glial fibrillary acidic protein (GFAP)^+^ astrocytes (4.9% ± 2.0% versus 9.3% ± 1.1%, p = 0.400), and cyclic nucleotide phosphodiesterase (CNPase)^+^ oligodendrocytes (0.4% ± 0.0% versus 0.4% ± 0.1%, p = 0.700) (Figures 1C, S1A, and S1B).

To test the functionality of hM4Di in neurons, we cotransfected a lentiviral vector encoding the Janelia green fluorescent protein-calmodulin fusion protein (jGCaMP7f) gene (Dana et al., 2019), which is a genetically encoded calcium indicator, under the control of the hSyn promoter into hM4Di-NS/PCs and mCherry-NS/PCs (Figure S2A). The NS/PCs were differentiated to obtain neurons, followed by 30 days of culture for maturation. The matured neurons exhibited sporadic calcium events, indicating spontaneous neural activities (Figures 1D and S2B, Videos S1, and S2). The addition of CNO significantly decreased the number of calcium events in neurons differentiated from hM4Di-NS/PCs (5.6 ± 0.8 events/min versus 1.1 ± 0.3 events/min, p < 0.001), whereas that in neurons differentiated from mCherry-NS/PCs did not differ significantly from the base line (4.9 ± 0.7 events/min versus 3.5 ± 1.1 events/min, p = 0.053) (Figure S2C). The normalized number of calcium events after CNO administration was significantly lower in hM4Di-expressing neurons than in the negative control (15.5% ± 4.4% versus 71.5% ± 19.3%, p = 0.016) (Figure 1E).

Video S1. Calcium imaging of hM4Di-NS/PC-derived neurons CNO (10 μM) was administered at 60 s.

Video S2. Calcium imaging of mCherry-NS/PC-derived neurons CNO (10 μM) was administered at 60 s.

To further confirm the electrophysiological activity in the cell population, a micro-electrode array (MEA) analysis was performed (Okubo et al., 2016; Sato et al., 2021). After 30 days of culture to allow neuronal maturation, neurons from both cell lines showed spontaneous activity before CNO administration (Figure 1F). The neuronal activity of cells differentiated from hM4Di-NS/PCs showed clear inhibition following the administration of CNO, whereas that of cells differentiated from mCherry-NS/PCs was not altered. The normalized spike counts after CNO administration revealed that neuronal activity was significantly inhibited by CNO administration in differentiated cells from hM4Di-NS/PCs (37.2% ± 5.1% versus 116.0% ± 10.0%, p < 0.001) (Figure 1G). Together, these results show that we generated hM4Di-expressing neurons from hiPSC-NS/PCs that were well modulated by CNO administration.

### Engrafted NS/PCs differentiated into neural cells in the injured spinal cord

We transplanted hM4Di-NS/PCs (hM4Di-TP group) and mCherry-NS/PCs (mCherry-TP group) into SCI mice at the subacute phase (Nori et al., 2011; Okubo et al., 2016). Histological analyses were performed to evaluate the characteristics of the engrafted cells at 10 weeks after transplantation.

**Figure 2:**
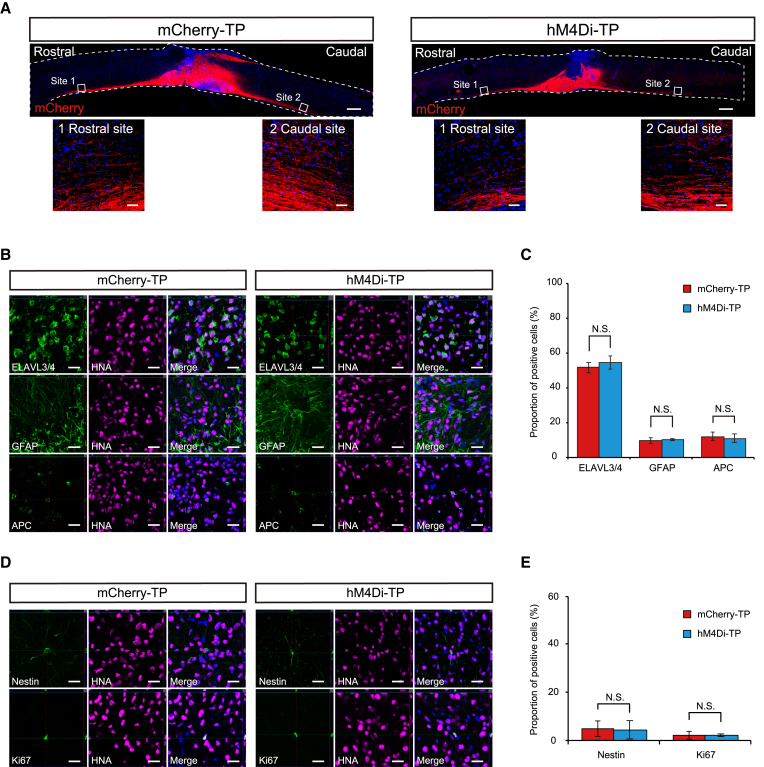
Histological appearance of a spinal cord with transplanted hiPSC-NS/PCs (A) Representative image of sagittal sections stained for mCherry (left, mCherry-TP; right, hM4Di-TP). mCherry-positive engrafted neurons were elongated rostrally and caudally. Scale bars, 500 and 50 μm (enlarged images). (B) Representative images of neural cells differentiated from graft cells of the mCherry-TP group (left) and hM4Di-TP group (right). HNA (human cell)-positive engrafted cells merged with ELAVL3/4 (neurons), GFAP (astrocytes), and APC (oligodendrocytes) in each transplanted group. Scale bars, 20 μm. (C) Histograms showing the quantification of three engrafted neural lineage cells in each transplanted group (mCherry-TP group, n = 6; hM4Di-TP group, n = 6). (D) Representative images of immature graft cells in the mCherry-TP group (left) and hM4Di-TP group (right). HNA (human cell)-positive engrafted cells merged with Nestin (immature cell) and Ki67 (immature cell) in each transplanted group. Scale bars, 20 μm. (E) Histograms showing the quantification of immature cells in engrafted cells (mCherry-TP group, n = 6; hM4Di-TP group, n = 6). Not significant (N.S.) according to the Mann-Whitney U test (C and E). The data are presented as the mean ± SEM.

mCherry-positive fibers showed graft-derived neuronal axons that extended caudally and rostrally from the engrafted site (Figure 2A). The neural differentiation and maturation of the engrafted cells were evaluated by double staining with human nuclear antigen (HNA) and cell-type-specific markers (Figures 2B and 2D). In both groups, the quantified proportions did not differ for ELAVL3/4^+^ neurons (54.5% ± 3.9% versus 51.6% ± 3.1%, p = 0.699), GFAP^+^ astrocytes (10.2% ± 0.6% versus 9.6% ± 1.6%, p = 0.589), or adenomatous polyposis coli CC-1 (APC)^+^ oligodendrocytes (10.8% ± 2.4% versus 11.9% ± 2.5%, p = 0.699) (Figure 2C). Furthermore, no differences were observed in the number of immature cells quantified by the neural stem cell marker Nestin (4.4% ± 0.8% versus 5.0% ± 1.0%, p = 0.589) or the cell proliferation marker Ki67 (2.3% ± 0.3% versus 2.3% ± 0.1%, p = 0.699) (Figure 2E). In addition to the immunohistochemical (IHC) features, no tumor-like tissue formation of engrafted cells was observed by hematoxylin and eosin (H&E) staining (Figures S3A and S3B). These results demonstrated that transplanted NS/PCs dominantly differentiated into mature neurons and did not show different differentiation characteristics or tumorigenic changes that could be affected by the induction of each lentiviral vector.

### Neurons differentiated from the grafted NS/PCs connected to host neurons by synaptic formation

**Figure 3:**
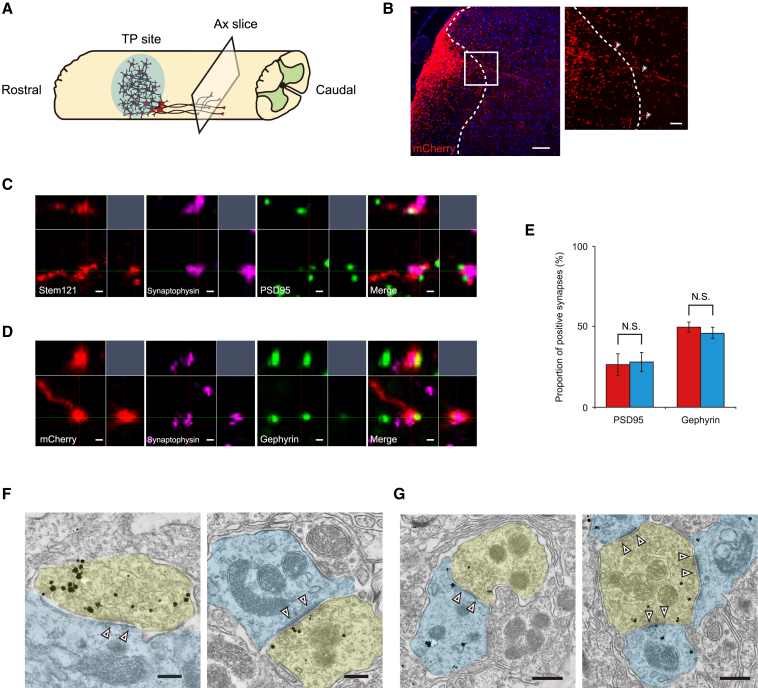
Synaptic formation of neurons derived from transplanted NS/PCs (A and B) Schema (A) and representative image (B) of axial sections 4 mm caudal to the injury site stained for mCherry. mCherry-positive fibers elongated into the gray matter (arrowheads). Scale bars, 100 and 20 μm (enlarged image). (C) High-magnification view of excitatory synaptic formation. The presynaptic marker synaptophysin merged with Stem121 and adjacent to the excitatory postsynaptic marker postsynaptic density 95. Scale bars, 0.5 μm. (D) High-magnification view of inhibitory synaptic formation. The presynaptic marker synaptophysin merged with mCherry and adjacent to the inhibitory postsynaptic marker gephyrin. Scale bars, 0.5 μm. (E) Histograms showing the quantification of excitatory (PSD95^+^) and inhibitory (gephyrin^+^) synapses in each transplanted group (mCherry-TP group, n = 3; hM4Di-TP group, n = 3). (F) Immunoelectron microscopy image of a synaptic connection (white arrowheads) from immunogold-labeled mCherry-positive graft neuron (yellow) to host neuron (light blue). Symmetrical connection (left) and asymmetrical connection (right) were observed. Scale bars, 200 nm. (G) Immunoelectron microscopy image of a synaptic connection (white arrowhead) between two graft neurons. The presynaptic button containing synaptic vesicles (yellow) and postsynaptic button without vesicles (light blue) are both immunogold labeled with mCherry. Symmetrical connection (left) and asymmetrical connection (right) were observed. Scale bars, 500 nm. Not significant (N.S.) according to the Mann-Whitney U test (E). The data are presented as the mean ± SEM.

To determine the ability of the transplanted neurons to integrate into host neuronal circuitry, we histologically assessed the synaptic formation of graft neurons. IHC staining of mCherry demonstrated that the descending graft fibers penetrated into the gray matter from the white matter (Figures 3A and 3B). The presynaptic boutons of grafted cells colocalized with both the excitatory postsynapse button marker postsynaptic density protein 95 (PSD95) and the inhibitory postsynapse button marker gephyrin (Figures 3C and 3D). Quantitative analysis revealed the inhibitory dominant proportion of graft-to-host synaptic connections (PSD95-positive synapse, 26.5% ± 6.6% versus 27.8% ± 5.8%, p = 1.000; gephyrin-positive synapse, 49.5% ± 3.1% versus 45.8% ± 3.5%, p = 0.400) (Figure 3E).

Additional evaluation of synaptic formation was performed by immunoelectron microscopy analysis. Connections suggesting excitatory and inhibitory synapses were confirmed in both grafted-to-host neurons and grafted-to-grafted neurons (Figures 3F and 3G) (Gray, 1959).

### Transplanted NS/PCs enhanced functional recovery following SCI

To evaluate the therapeutic effect of NS/PC transplantation, recovery of locomotor function was assessed by the Basso mouse scale (BMS) and treadmill gait analysis (Basso et al., 2006; Okubo et al., 2016).

**Figure 4:**
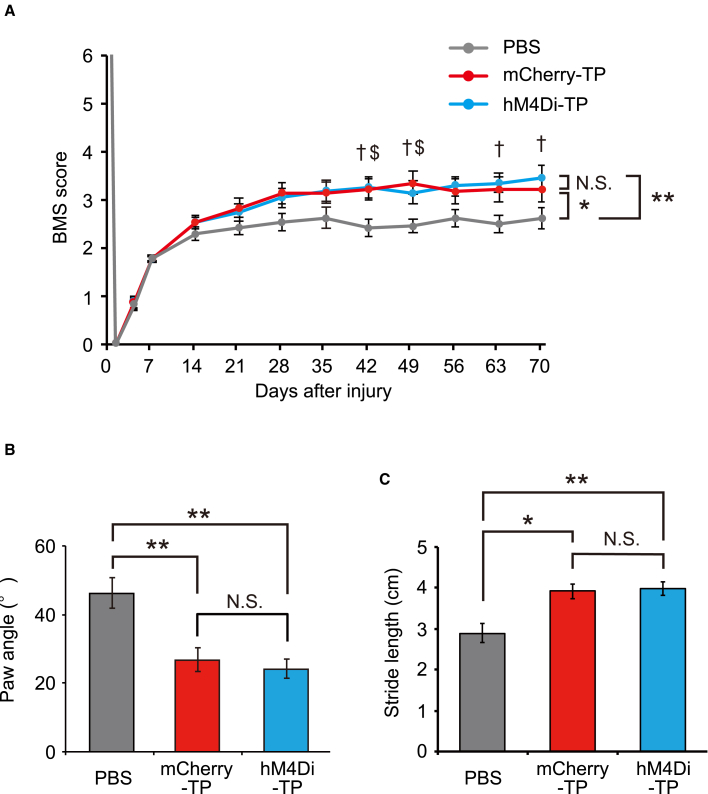
Transplanted NS/PCs promote functional recovery after SCI (A) BMS scores before and after SCI in non-transplanted animals (PBS) and transplanted animals (mCherry-TP and hM4Di-TP) (PBS group, n = 20; mCherry-TP group, n = 18; hM4Di-TP group, n = 20). (B) Comparison of paw angles determined by treadmill gait analysis performed at 9 weeks after transplantation (PBS group, n = 20; mCherry-TP group, n = 18; hM4Di-TP group, n = 20). (C) Comparison of stride lengths determined by treadmill gait analysis performed at 9 weeks after transplantation (PBS group, n = 20; mCherry-TP group, n = 18; hM4Di-TP group, n = 20). ^∗^p < 0.05, ^∗∗^p < 0.01, and N.S., not significant according to the repeated-measures two-way ANOVA (A) and the Mann-Whitney U test following Kruskal-Wallis test (B and C). ^†^p < 0.05, hM4Di-TP versus PBS, and ^$^p < 0.05, mCherry-TP versus PBS, according to the two-sided unpaired Student t test. The data are presented as the mean ± SEM. All multiple testing data analyses were followed by Bonferroni correction.

The BMS score was significantly improved by NS/PC transplantation, whereas no significant difference in the BMS score was detected between the two transplanted groups (repeated two-way ANOVA, p = 0.007; *post hoc* hM4Di-TP versus PBS, adjusted p = 0.003; mCherry-TP versus PBS, adjusted p = 0.027; hM4Di-TP versus mCherry-TP, adjusted p = 1.000) (Figure 4A). In treadmill gait analysis performed at 9 weeks after transplantation, animals in the transplanted groups showed significantly narrower paw angles and longer stride lengths than those in the non-transplanted group (paw angle: hM4Di-TP, 24.3° ± 2.7°; mCherry-TP, 26.9° ± 3.4°; PBS, 46.1° ± 4.4°; Kruskal-Wallis test, p < 0.001; *post hoc* hM4Di-TP versus PBS, adjusted p < 0.001; mCherry-TP versus PBS, adjusted p = 0.006; hM4Di-TP versus mCherry-TP, adjusted p = 1.000; stride length: hM4Di-TP, 4.0 ± 0.2 cm; mCherry-TP, 3.9 ± 0.2 cm; PBS, 2.9 ± 0.2 cm; Kruskal-Wallis test, p = 0.002; *post hoc* hM4Di-TP versus PBS, adjusted p = 0.003; mCherry-TP versus PBS, adjusted p = 0.012; hM4Di-TP versus mCherry-TP, adjusted p = 1.000) (Figures 4B and 4C). In contrast, neither the paw angle nor the stride length differed between the hM4Di-TP group and the mCherry-TP groups. From these results, transplanting NS/PCs enhanced locomotor functional recovery after SCI. In addition, there was no difference in functional recovery after hM4Di-NS/PC and mCherry-NS/PC transplantation.

### The locomotor function of hM4Di-NS/PC-transplanted mice was controlled by CNO administration

**Figure 5:**
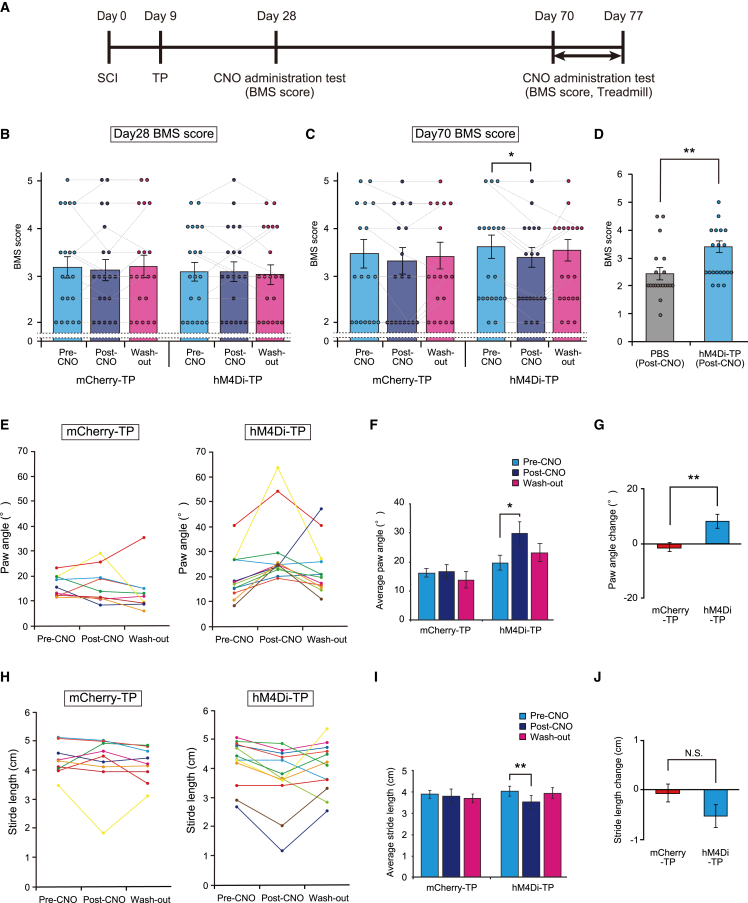
hM4Di-NS/PC-transplanted mice showed locomotor functional changes induced by the inhibition of graft neuronal activity (A) Schematic representing the time schedule of the *in vivo* experiment. (B and C) BMS scores before CNO administration, after CNO administration, and after CNO washout. The scores at 28 days after SCI (B) and 70 days after SCI (C) are presented (mCherry-TP group, n = 18; hM4Di-TP group, n = 20). (D) Comparison of BMS scores in CNO-administered mice on day 70 (PBS group, n = 20; hM4Di-TP group, n = 20). (E) Raw data of paw angles before CNO administration, after CNO administration, and after CNO washout for mCherry-TP mice (left) and hM4Di-TP mice (right). (F) Quantification of paw angles before CNO administration, after CNO administration, and after CNO washout (mCherry-TP group, n = 9; hM4Di-TP group, n = 12). (G) Quantification of the paw angle changes before and after CNO administration in each animal (mCherry-TP group, n = 9; hM4Di-TP group, n = 12). (H) Raw stride-length data before CNO administration, after CNO administration, and after CNO washout for mCherry-TP mice (left) and hM4Di-TP mice (right). (I) Quantification of stride length before CNO administration, after CNO administration, and after CNO washout (mCherry-TP group, n = 9; hM4Di-TP group, n = 12). (J) Quantification of the stride length changes before and after CNO administration in each animal (mCherry-TP group, n = 9; hM4Di-TP group, n = 12). ^∗^p < 0.05, ^∗∗^p < 0.01, and N.S., not significant (not displayed in the figures [B, C, F, and I]), according to the Wilcoxon signed rank test following Friedmann test (B, C, F, and I) and the Mann-Whitney U test (D, G, and J). The data are presented as the mean ± SEM (B–D, F, G, I, and J). All multiple testing data analyses were followed by Bonferroni correction.

Finally, we evaluated the effect of transplanted neurons on the locomotor function of host mice. Functional behavior was assessed before (pre-CNO), 1 h after (post-CNO), and 24 h after (washout) CNO administration. The BMS score with CNO administration was assessed at 3 and 9 weeks after transplantation. Treadmill gait analysis with CNO administration was performed at 9 weeks after transplantation (Figure 5A).

After administration of CNO at 3 weeks after transplantation, no functional changes were observed in the hM4Di-TP group or mCherry-TP group (mCherry-TP: pre-CNO, 3.2 ± 0.2; post-CNO, 3.1 ± 0.2; washout, 3.2 ± 0.3, Friedman test, p = 0.651; hM4Di-TP: pre-CNO, 3.1 ± 0.2; post-CNO, 3.1 ± 0.2; washout, 3.0 ± 0.2, Friedman test, p = 0.468) (Figure 5B). In contrast, at 9 weeks after transplantation, the hM4Di-TP group showed a significantly lower BMS score after administration of CNO, whereas the score in the mCherry-TP group was not significantly different (mCherry-TP: pre-CNO, 3.2 ± 0.3; post-CNO, 3.1 ± 0.3; washout, 3.2 ± 0.2, Friedman test, p = 0.143; hM4Di-TP: pre-CNO, 3.3 ± 0.2; post-CNO, 3.1 ± 0.2; washout, 3.3 ± 0.2, Freidman test, p = 0.010; *post hoc* pre-CNO versus post-CNO, adjusted p = 0.021; pre-CNO versus washout, adjusted p = 1.000; post-CNO versus washout, adjusted p = 0.174) (Figure 5C). Although the BMS scores of hM4Di-TP mice declined following the inhibition of graft neuronal activity, the locomotor functions of mice in this condition remained refined compared with those of the non-transplanted animals, which showed no functional change after the administration of CNO (PBS post-CNO, 2.4 ± 0.2, hM4Di-TP versus PBS, p = 0.009) (Figures 5D, S4A, and S4B).

In the treadmill gait analysis test, the paw angle of the hM4Di-TP group was significantly enlarged following the administration of CNO (pre-CNO, 19.8° ± 2.6°; post-CNO, 29.9° ± 4.1°; washout, 23.2° ± 3.1°, Friedman test, p = 0.013; *post hoc* pre-CNO versus post-CNO, adjusted p = 0.009; pre-CNO versus washout, adjusted p = 0.717; post-CNO versus washout, adjusted p = 0.150) (Figures 5E and 5F). In addition, the stride length was significantly reduced after CNO administration in the hM4Di-TP group (pre-CNO, 4.2 ± 0.2 cm; post-CNO, 3.7 ± 0.3 cm; washout, 4.0 ± 0.2 cm, Friedman test, p = 0.013; *post hoc* pre-CNO versus post-CNO, adjusted p = 0.009; pre-CNO versus washout, adjusted p = 1.000; post-CNO versus washout, adjusted p = 0.618) (Figures 5H and 5I). In contrast, the parameters of treadmill gait analysis in the mCherry-TP group and the PBS group did not differ before and after CNO administration (mCherry-TP, paw angle: pre-CNO, 16.2° ± 1.4°; post-CNO, 16.6° ± 2.5°; washout, 13.8° ± 2.9°, Friedman test, p = 0.236; stride length: pre-CNO, 4.4 ± 0.2 cm; post-CNO, 4.3 ± 0.3 cm; washout, 4.2 ± 0.2 cm, Friedman test, p = 0.097) (Figures 5E, 5F, 5H, 5I, and S4C–S4F). The effect of CNO in the hM4Di-TP group tended to return to the basal levels after CNO withdrawal. In contrast, CNO administration did not alter the paw angles and stride lengths of mice in the mCherry-TP group. Assessment of functional changes before and after CNO administration revealed a significantly larger functional decline in the paw angle in the hM4Di-TP group compared with the mCherry-TP group and a tendency for a more substantial change in stride length in the hM4Di-TP group (paw angle: 10.2° ± 2.8° versus 0.4° ± 1.8°, p = 0.009; stride length: −0.5 ± 0.1 cm versus −0.1 ± 0.2, p = 0.069) (Figures 5G and 5J).

Together, the results show that locomotor function of only the hM4Di-TP group was controlled by CNO administration, suggesting that inhibiting the neuronal activity of the graft neurons adversely affected the locomotor functions of the host mice.

### The functional change induced by graft neuronal activity inhibition was sustained in the mice during long-term follow-up

**Figure 6:**
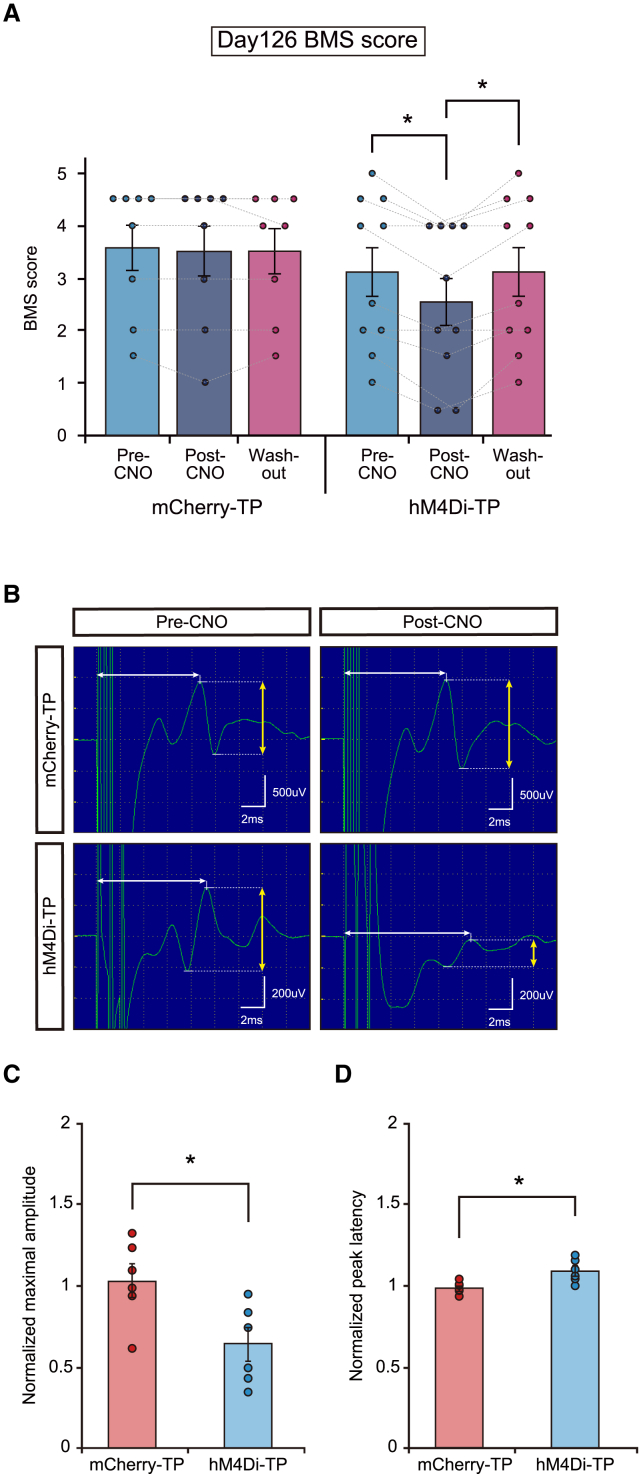
Neuronal activity inhibition of engrafted cells affected the locomotor function and electrophysiological parameters in long-term-followed animals (A) BMS scores before CNO administration, after CNO administration, and after CNO washout (mCherry-TP group, n = 8; hM4Di-TP group, n = 10). (B) Representative images of MEP waves in mCherry-TP animals and hM4Di-TP animals (left, pre-CNO; right, post-CNO). (C) Average of normalized maximal amplitude in mCherry-TP group and hM4Di-TP group (mCherry-TP group, n = 6; hM4Di-TP group, n = 6). (D) Average of normalized peak latency in mCherry-TP group and hM4Di-TP group (mCherry-TP group, n = 6; hM4Di-TP group, n = 6). ^∗^p < 0.05 according to the Wilcoxon signed rank test following Friedman test (A) and the Mann-Whitney U test (C and D). The data are presented as the mean ± SEM (A, C, and D). All multiple testing data analyses were followed by Bonferroni correction.

To confirm the sustained therapeutic effect of graft neuronal activity, we administered CNO to mice followed for 17 weeks after transplantation. Similar to the experiment at 9 weeks after transplantation, the BMS score significantly decreased in the hM4Di-TP group after administration of CNO (mCherry-TP: pre-CNO, 3.6 ± 0.4; post-CNO, 3.5 ± 0.5; washout, 3.5 ± 0.4; Friedman test, p = 0.607; hM4Di-TP: pre-CNO, 3.1 ± 0.5; post-CNO, 2.6 ± 0.5; washout, 3.1 ± 0.5, Friedman test, p < 0.001; *post hoc* pre-CNO versus post-CNO, adjusted p = 0.027; pre-CNO versus washout, adjusted p = 1.000; post-CNO versus washout, adjusted p = 0.027) (Figure 6A). The parameters of the treadmill gait analysis in hM4Di-TP animals showed a significantly larger change in paw angle and tended to show a decrease in stride length (Figures S5A–S5F).

Electrophysiological analysis by neuronal activity inhibition was also performed. The motor evoked potential (MEP) wave was analyzed before and after CNO administration, and the post-CNO parameters were normalized based on the pre-CNO parameters. The normalized maximal amplitude was significantly lower and the normalized peak latency was significantly longer in the hM4Di-TP group compared with the mCherry-TP group (normalized maximal amplitude 1.0 ± 0.1 versus 0.6 ± 0.1, p = 0.026; normalized peak latency 1.0 ± 0.04 versus 1.1 ± 0.03, p = 0.015) (Figures 6B and 6C). Accordingly, the functional decline of host animals by inhibiting engrafted neuronal activity was sustained in long-term-followed animals.

### Trans-synaptic tracing revealed the integration of engrafted neurons into the host motor circuit

**Figure 7:**
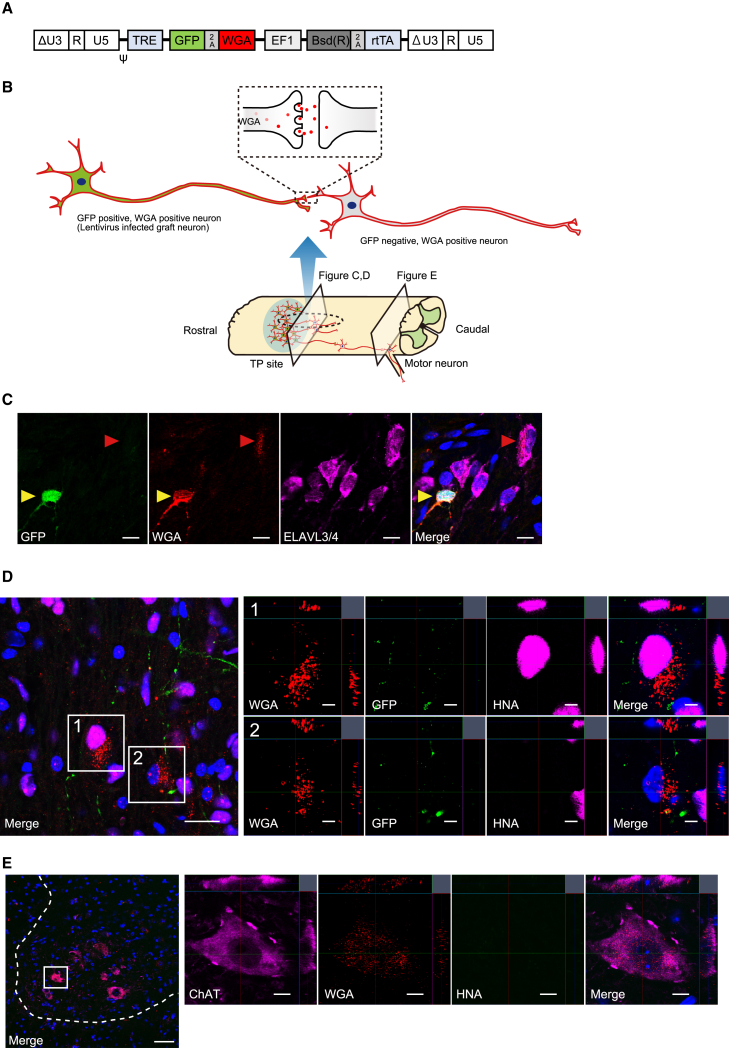
WGA expressed in graft neurons demonstrates the trans-synaptic integration into host neuronal circuits (A) Schematic illustration of the integrated proviral form of the Tet-inducible lentiviral vector CSIV-TRE-AcGFP1-WGA-EF1a-Bsd(R)-rtTA2M2. (B) Schematic image of the experiments. TP site, transplantation site. (C) Representative images of migrating WGA. The yellow arrowheads indicate lentivirus-infected graft neurons (GFP, WGA double-positive neurons) and the red arrowheads indicate migrated WGA in GFP-negative neurons. Scale bars, 10 μm. (D) GFP, WGA, and HNA staining identifies cells connected to lentivirus-infected neurons. Enlarged image 1 shows a non-lentivirus-infected graft cell stained with WGA. Enlarged image 2 shows a host cell stained with WGA. Scale bars, 20 and 5 μm (enlarged images). (E) HNA, WGA, and ChAT staining revealed host motor neurons connected to lentivirus-infected neurons. Scale bars, 50 and 10 μm (enlarged images). Dashed whitline showes the edge of gray matter.

As presented above, differentiated neuronal cells directly affected locomotor function. To clarify the integration of graft neurons into the host motor circuitry, trans-synaptic tracing by wheat germ agglutinin (WGA) was performed (Fabian and Coulter, 1985). A Tet-inducible lentiviral vector containing the AcGFP1 and WGA genes linked by the porcine teschovirus-1 2A peptide sequence was transfected into NS/PCs (WGA-NS/PCs) (Figure 7A) (Ohashi et al., 2011). NS/PCs were transplanted into mice with thoracic contusion injuries in the subacute phase, and the mice were fed a rodent diet containing doxycycline for 2 weeks before their spinal cord sections were extracted (Kojima et al., 2019).

IHC analyses were performed to confirm the presence of WGA^+^/AcGFP1^−^ cells, indicating trans-synaptic migration of WGA and thereby suggesting the synaptic connection of transplanted neurons (Figure 7B). Migration of WGA to ELAVL3/4^+^ neurons was confirmed, indicating the synaptic connection of graft neurons with other neurons (Figure 7C). WGA showed migration to both rostral and caudal sites. The WGA-positive area decreased with distance from the transplanted site; however, WGA was still observed 4 mm caudal to the epicenter (Figures S6A and S6B). As for migrated cells, WGA was detected not only in HNA^+^/AcGFP1^−^ graft cells but also in HNA^−^ host cells, demonstrating the synaptic connection between graft neurons and host neurons (Figure 7D). To validate the connection with host motor neurons, we evaluated the migration of WGA to choline acetyltransferase (ChAT)^+^ motor neurons at the caudal site (Figure 7E). WGA was confirmed in the ChAT^+^ host motor neurons in the region of lumbar enlargement, demonstrating that the engrafted neurons were integrated into the host motor circuits.

## Discussion

By generating hM4Di-expressing hiPSC-NS/PCs, we have shown the chemical inhibition of neuronal activity via a reduction in intracellular calcium events and attenuation of electrical activity. The precise and remote inhibition of neuronal activity was confirmed *in vitro*, and when these cells were transplanted during the subacute phase of SCI, recovered locomotor function at the chronic stage transiently declined following the administration of CNO in host animals. This neuronal function was corroborated by the trans-synaptic tracing of WGA, revealing the integration of grafted neurons into the host motor circuits. These results directly demonstrated the significance of neuronal activity in grafted hiPSC-NS/PCs for behavioral improvement after SCI.

Intriguingly, loss of motor function induced by the DREADD was not observed at 3 weeks but was observed after 9 weeks after transplantation, indicating that the grafted neurons remained immature at the early stage after transplantation and reached maturity over time, thereby leading to locomotor function. In parallel to these findings, our previous histological analyses revealed that NeuN^+^ mature neuronal cell numbers increased gradually from 6 to 14 weeks after hiPSC-NS/PC transplantation (Nori et al., 2011). These results were corroborated by other *in vitro* studies revealing that cultured hiPSC-derived neurons required approximately 6 weeks to form synaptic connections and evoke spontaneous neuronal activity (Meijer et al., 2019; Wilson and Newell-Litwa, 2018). Together, these findings suggest that a certain amount of time is required for the transplanted NS/PCs to differentiate and mature into functional neurons, which contributes to locomotor improvement.

On the other hand, the DREADD-mediated inhibition of graft neuronal activity only partially deteriorated locomotor function, which did not reach the level of that in non-transplanted SCI animals. This suggests that graft-derived beneficial factors other than neuronal function influenced locomotor recovery. A candidate factor underlying this result is neuroprotection by the trophic factors. We previously demonstrated the secretion of nerve growth factor, brain-derived neurotrophic factor, and hepatocyte growth factor from hiPSC-derived NS/PCs, and these factors exerted a favorable effect on behavior soon after transplantation (Nori et al., 2011). The slight improvement in the early phase after transplantation could be theorized as the effect of these factors. An alternative factor for the beneficial mechanisms was remyelination by the transplanted NS/PC-derived oligodendrocytes (Assinck et al., 2017; Kawabata et al., 2016; Nori et al., 2017). However, the therapeutic effect of remyelination is still controversial (Duncan et al., 2018; Yasuda et al., 2011). The present study revealed that the frequency of oligodendrocyte differentiation was approximately 10%, which was noticeably lower than the more than 50% rate for neuronal differentiation. Although a small population of differentiated myelinating cells might contribute to locomotion, further detailed studies are required to estimate the contribution of differentiated oligodendrocytes. Taken together, hiPSC-NS/PCs most likely promote functional recovery via multiple mechanisms, and the present study demonstrated at least the significance of differentiated neuronal cells that directly contribute to locomotion.

The safety of using hiPSCs as a cell source for NS/PCs must be verified due to tumorigenicity concerns (Iida et al., 2017; Tanimoto et al., 2020). We have previously addressed this issue by using a Notch signal inhibitor (γ-secretase inhibitor) to suppress cell proliferation and promote neuronal differentiation (Okubo et al., 2016, 2018). In the present study, we prevented tumor-like overgrowth by pretreating NS/PCs with this inhibitor and successfully engrafted neuronal cells constituting functional neuronal circuits. Since we plan to use this inhibitor in actual clinical trials (Tsuji et al., 2019), our strategy of using hiPSCs for SCI could be validated in terms of both efficacy and safety.

The results of the current study raise the question of which neuronal cells derived from the graft most influence the locomotor function of host animals. Although there are several reports indicating functional recovery by maintaining the inhibitory and excitatory balance of neurons following SCI (Chen et al., 2018; Fandel et al., 2016; Huang et al., 2016), our present study did not reveal which subtype of neuronal cells derived from the graft contributed to functional recovery. In addition, the connectivity of grafted neurons to each spinal tract is a matter of great concern and could also be important for clarifying the therapeutic mechanisms (Adler et al., 2017; Kumamaru et al., 2018, 2019). Further investigation will uncover more detailed functional contributions of NS/PC transplantation to the treatment of SCI.

In conclusion, we have successfully controlled the activity of neurons derived from hiPSC-NS/PCs in the injured spinal cord and confirmed the change in locomotor function induced by the suppression of graft-derived neuronal activity. The present study provided some mechanistic insight into functional recovery from SCI after hiPSC-NS/PC transplantation.

## Experimental procedures

### Lentiviral vector preparation

Recombinant lentiviral vector production was performed as described previously (Miyoshi et al., 1998). The details are described in the supplemental information.

### Cell culture, lentiviral transduction, and *in vitro* neuronal differentiation assay

NS/PCs were cultured from a non-tumorigenic hiPSC clone (414C2), and lentiviral transduction was performed as previously reported (Nori et al., 2011; Okada et al., 2008; Okubo et al., 2018).

Differentiated NS/PCs were cultured for 30 days and ICC stained to evaluate the differentiation potential. Cultured cells were also evaluated by calcium imaging and MEA analyses. The detailed methods are described in the supplemental information.

### SCI animal model and NS/PC transplantation

Contusive SCI was induced at the level of the 10^th^ thoracic spinal vertebra in immunodeficient mice. Transplantation of NS/PCs was performed at 9 days after injury as previously reported (Kamata et al., 2021; Okubo et al., 2016). The details are provided in the supplemental information.

### Histological analyses

Histological analyses were performed by H&E staining and IHC staining. The detailed methods and antibodies used for IHC staining are described in the supplemental information.

### Immunoelectron microscopy analysis

Immunoelectron microscopy analysis was performed on spinal cord sections from hM4Di-TP mice and mCherry-TP mice. The detailed procedure was described previously (Shibata et al., 2019) and is provided in the supplemental information.

### Neural activity inhibition assay (*in vivo*)

BMS scores (Basso et al., 2006) and treadmill gait analysis were evaluated before, 1 h after, and 24 h after CNO (10 mg/kg) administration. The detailed methods are described in the supplemental information.

### Electrophysiology

Electrophysiological experiments were performed in long-term-followed hM4Di-TP mice and mCherry-TP mice. The detailed methods are described in the supplemental information.

### Statistical analyses

Statistical analyses were performed using SPSS statistics (Japan IBM, Tokyo, Japan, v.26.0.0.0). Data are reported as the mean ± SEM. The sample sizes are indicated in the respective figure legends. The Mann-Whitney U test was performed for the *in vitro* ICC staining, calcium imaging, MEA imaging, *in vivo* IHC staining, MEP analysis, BMS score, and treadmill gait analyses in a non-paired situation. The Wilcoxon signed rank test was used to compare the BMS score and the treadmill parameter changes in the neuronal activity inhibition assay. Repeated-measures two-way ANOVA was used for the weekly BMS scoring followed by two-sided unpaired Student's t test for each time point. All multiple testing data were analyzed by the Kruskal-Wallis test (non-paired) or Friedman test (paired) and *post hoc* analysis with Bonferroni correction (described as adjusted p). *p* values of <0.05 indicate statistical significance (^∗^p < 0.05 and ^∗∗^p < 0.01).

## Author contributions

T.K. designed the project, performed most of the experiments, and interpreted the data. T.K. and N.N. wrote the manuscript. N.N., Y.K., M.K., K.A., K.K., R.S., Y.S., K.I., M.S., J.K., T.S., and S.S. provided experimental support and ideas for the project. M.N. and H.O. supervised the overall project. T.K., N.N., H.O., and M.N. provided financial support. N.N., M.M., M.N., and H.O. edited the manuscript and gave administrative support. All authors read and approved the final manuscript.

## Conflict of interests

H.O. and M.N. are compensated scientific consultants to K Pharma, and H.O. is also a compensated scientific consultant to SanBio Ltd. No management, preparation, analysis, interpretation, or review of data was performed by the funding sources.

## Supplemental information

Document S1. Supplemental experimental procedures and Figures S1–S6

Document S2. Article plus supplemental information

## Footnotes

Supplemental information can be found online at https://doi.org/10.1016/j.stemcr.2021.12.005.
