# Supplementary material for: Modulation by DREADD reveals the therapeutic effect of human iPSC-derived neuronal activity on functional recovery after spinal cord injury
Source: Stem Cell Reports. 2022 Jan 11;17(1):127–42. doi: 10.1016/j.stemcr.2021.12.005 (PMC8758967; doi:10.1016/j.stemcr.2021.12.005)
Supplement: Document S1. Supplemental experimental procedures and Figures S1–S6 [file mmc1.pdf]

Stem Cell Reports, Volume *17*

Supplemental Information

Modulation by DREADD reveals the therapeutic effect of human iPSC--derived neuronal activity on functional recovery after spinal cord injury

Takahiro Kitagawa, Narihito Nagoshi, Yasuhiro Kamata, Momotaro Kawai, Kentaro Ago, Keita Kajikawa, Reo Shibata, Yuta Sato, Kent Imaizumi, Tomoko Shindo, Munehisa Shinozaki, Jun Kohyama, Shinsuke Shibata, Morio Matsumoto, Masaya Nakamura, and Hideyuki Okano

Supplemental Information

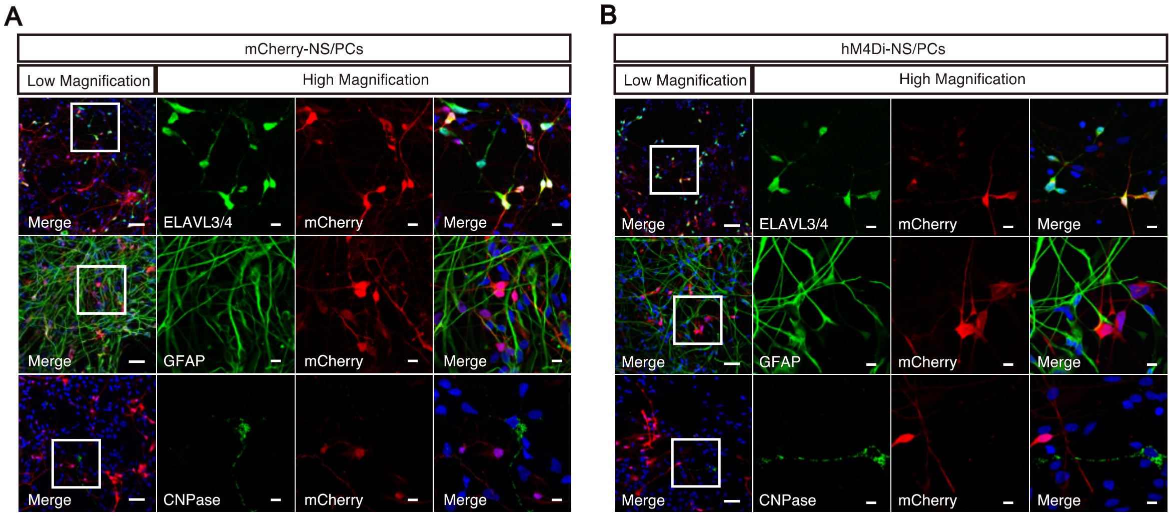

Figure S1. *In vitro* differentiation potential of hM4Di-NS/PCs and mCherry-NS/PCs

(A and B) Representative immunocytochemical images of three neural lineage cell types in mCherry-NS/PCs (A) and hM4Di-NS/PCs (B): ELAVL3/4 (neurons), GFAP (astrocytes), CNPase (oligodendrocytes). Scale bar, 50 μm for low-magnification images and 10 μm for high-magnification images.

ELAVL3/4: embryonic lethal abnormal vision-like protein 3/4, GFAP: glial fibrillary acidic protein, CNPase: cyclic nucleotide phosphodiesterase.

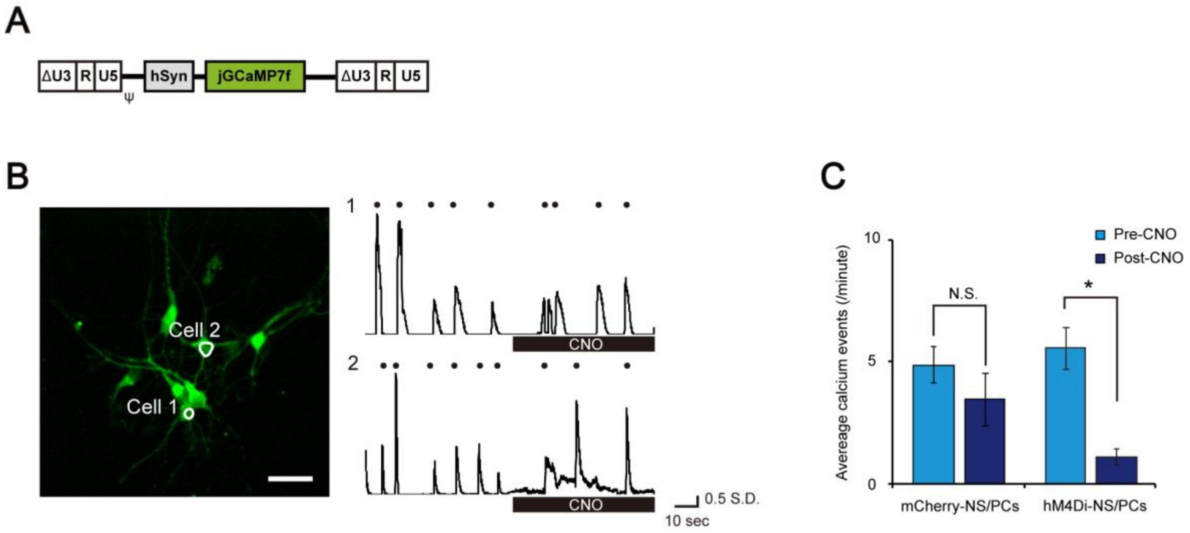

Figure S2. Calcium imaging of neurons differentiated from NS/PCs with CNO administration.

(A) Schematic illustration of the integrated proviral form of the lentiviral vector CSIV-hSyn-jGCaMP7f, which encodes the jGCaMP7f protein under the control of the hSyn promoter.

(B) Representative calcium transients of neurons differentiated from mCherry-NS/PCs. Left: An image of neurons expressing jGCaMP7f in a single field of view. Right: Time traces of representative cells highlighted with white outlines in the left image (the black dot indicates the calcium event). Scale bar, 30 μm.

(C) Average calcium events before and after CNO administration in neurons differentiated from mCherry-NS/PCs and hM4Di-NS/PCs (mCherry-NS/PCs, n = 15 cells / 3 independent experiments, hM4Di-NS/PCs, n = 18 cells / 3 independent experiments).

* $p < 0.05$ and not significant (N.S.) according to the Wilcoxon signed rank test (C). The data are presented as the mean ± SEM (C).

NS/PCs: neural stem/progenitor cells, CNO: clozapine N-oxide, hSyn: human synapsin

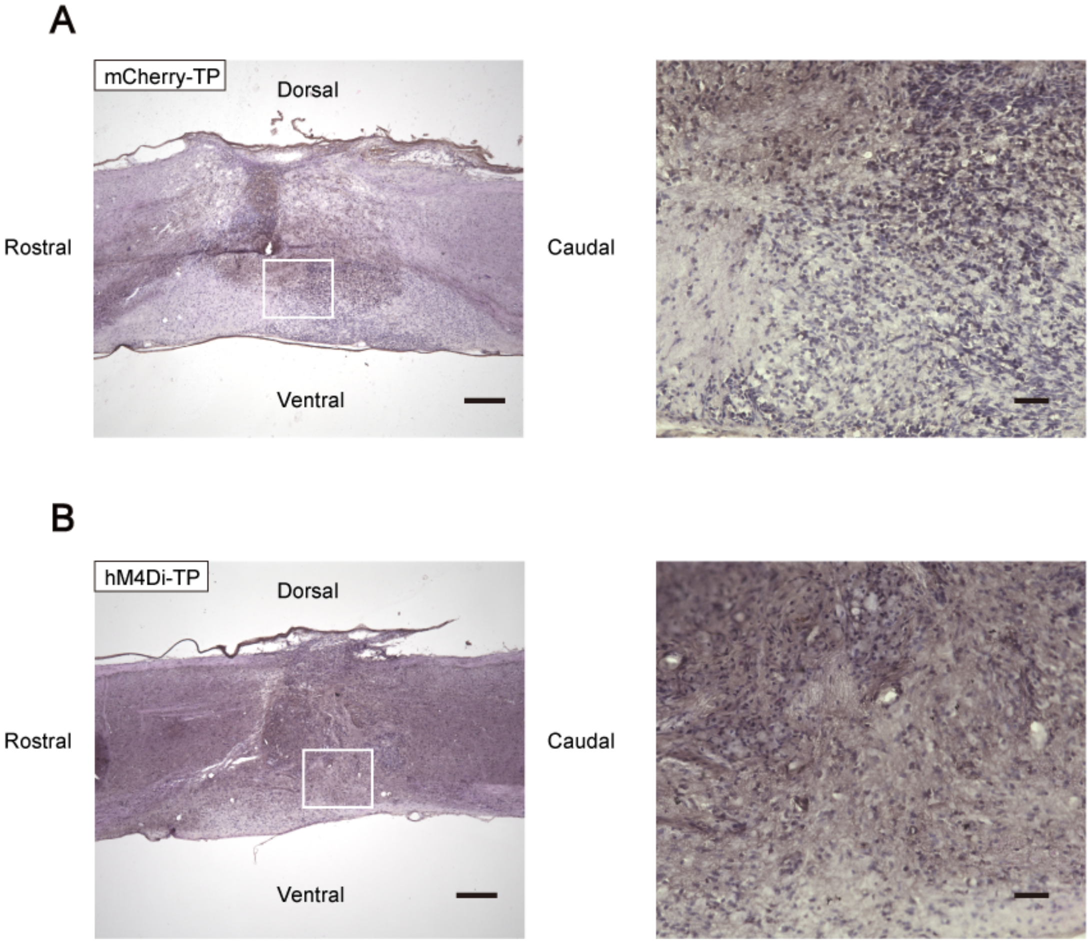

Figure S3. H&E staining of a spinal cord sections

(A and B) Representative images of H&E stained sections. No nuclear atypia nor rosette formation was observed. A: mCherry-TP group, B: hM4Di-TP group. Scale bar, 300μm for low-magnification images and 50 μm for high-magnification images.

TP: transplantation, H&E: hematoxylin-eosin

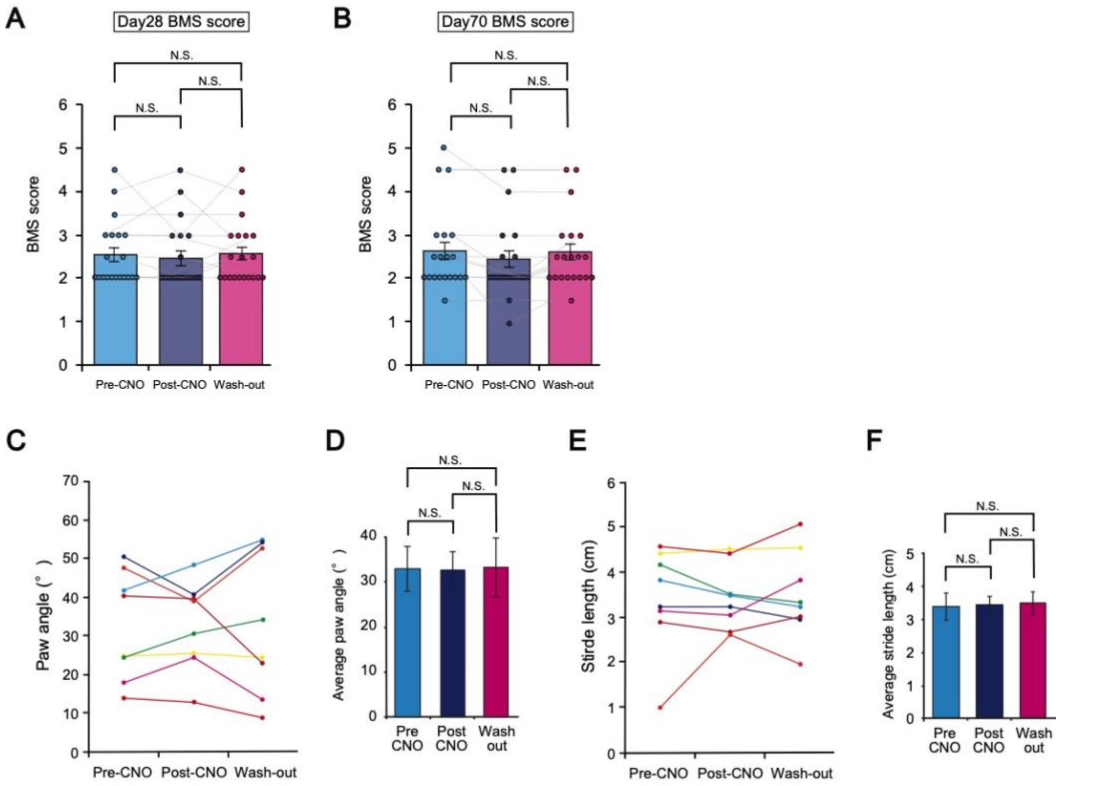

Figure S4. Neuronal activity inhibition assay for PBS group mice

(A and B) BMS scores before CNO administration, after CNO administration and after CNO washout. The scores at 28 days after SCI (A) and 70 days after SCI (B) are presented. (n = 20) (Day28; pre-CNO, 2.6 ± 0.2, post-CNO, 2.5 ± 0.2, wash-out, 2.6 ± 0.2, Friedman test, p = 0.895, day70; pre-CNO, 2.6 ± 0.2, post-CNO, 2.4 ± 0.2, wash-out, 2.6 ± 0.2, Friedman test, p = 0.016, *post hoc* pre-CNO vs. post-CNO, adjusted p = 0.114, pre-CNO vs. wash-out, adjusted p = 1.000, post-CNO vs. wash-out, adjusted p = 0.114).

(C) Raw data of paw angles before CNO administration, after CNO administration and after CNO wash-out for PBS-mice (n = 8).

(D) Quantification of paw angles before CNO administration, after CNO administration and after CNO wash-out for PBS mice (n = 8, pre-CNO, 32.9 ± 5.0°, post-CNO, 32.8 ± 4.0°, wash-out, 33.4 ± 6.6°, Friedman test, p = 1.000).

(E) Raw stride length data before CNO administration, after CNO administration and after CNO wash-out for PBS-mice (n = 8).

(F) Quantification of stride length before CNO administration, after CNO administration and after CNO wash-out for PBS-mice (n = 8, pre-CNO, 3.4 ± 0.4cm, post-CNO, 3.4 ± 0.3cm, wash-out, 3.5 ± 0.3cm, Friedman test, p = 0.687).

Not significant (N.S.) according to the Wilcoxon signed rank test following Friedmann test(A, B, D and F). The data are presented as the mean ± SEM (A, B, D and F).

TP: transplantation, BMS: Basso Mouse Scale, CNO: clozapine N-oxide.

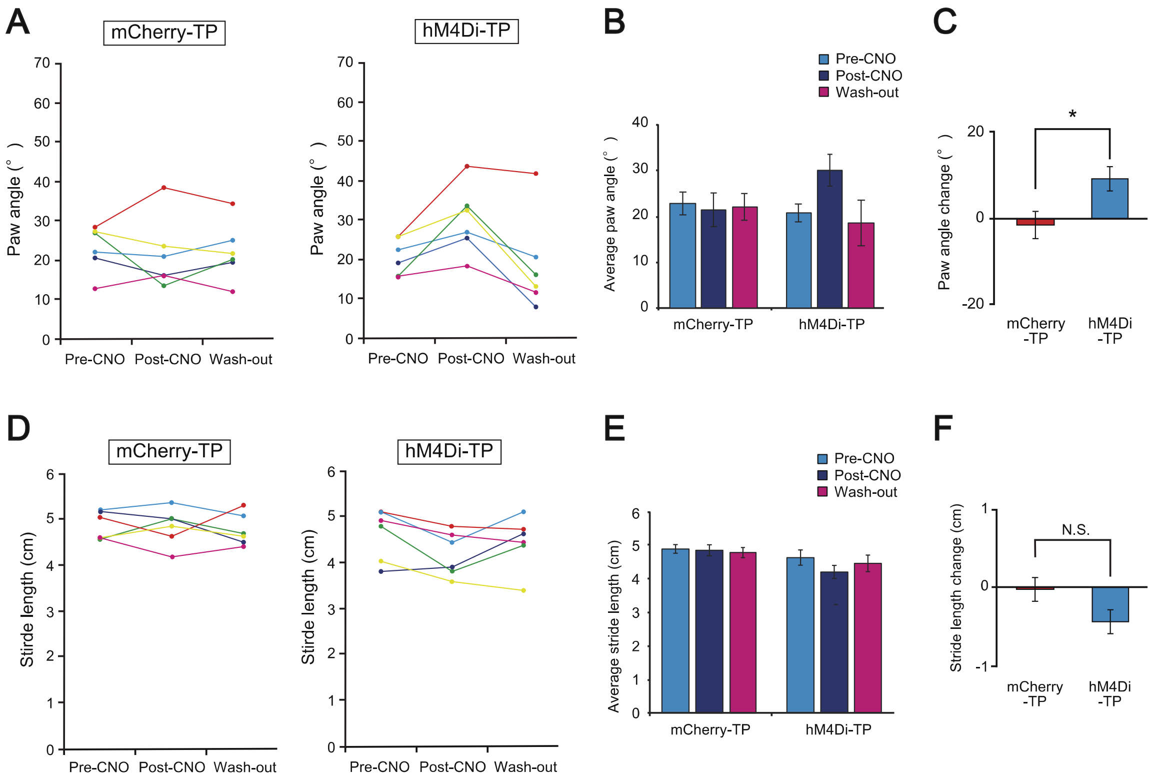

Figure S5. Treadmill analysis data in long-term followed animals

(A) Raw data of paw angles before CNO administration, after CNO administration and after CNO wash-out for mCherry-TP mice (left) and hM4Di-TP mice (right).

(B) Quantification of paw angles before CNO administration, after CNO administration and after CNO wash-out (mCherry-TP group, n = 6, hM4Di-TP group, n = 6, mCherry-TP: pre-CNO, 23.1 ± 2.4°, post-CNO, 21.5 ± 3.7°, wash-out, 22.3 ± 3.0°, Friedman test, p = 0.607, hM4Di-TP: pre-CNO, 20.9 ± 1.9°, post-CNO, 30.1 ± 3.5°, wash-out, 18.7 ± 5.0°, Friedman test, p = 0.009, *post hoc* pre-CNO vs. post-CNO, adjusted p = 0.084, pre-CNO vs. wash-out, adjusted p = 1.000, post-CNO vs. wash-out, adjusted p = 0.084).

(C) Quantification of the paw angle changes before and after CNO administration in each animal (mCherry-TP group, n = 6, hM4Di-TP group, n = 6, mCherry-TP, -1.6 ± 3.2 vs. hM4Di-TP, 9.2 ± 2.8, p = 0.041).

(D) Raw stride length data before CNO administration, after CNO administration and after CNO wash-out for mCherry-TP mice (left) and hM4Di-TP mice (right).

(E) Quantification of stride length before CNO administration, after CNO administration and after CNO wash-out (mCherry-TP group, n = 6, hM4Di-TP group, n = 6, mCherry-TP: pre-CNO, 4.9 ± 0.1cm, post-CNO, 4.9 ± 0.2cm, wash-out, 4.8 ± 0.1cm, Friedman test, $p = 0.846$, hM4Di-TP: pre-CNO, 4.6 ± 0.2cm, post-CNO, 4.2 ± 0.2cm, wash-out, 4.5 ± 0.2cm, Friedman test, $p = 0.135$).

(F) Quantification of the stride length changes before and after CNO administration in each animal (mCherry-TP group, n = 6, hM4Di-TP group, n = 6, mCherry-TP, 0.0 ± 0.2cm vs. hM4Di-TP, -0.4 ± 0.1cm, $p = 0.093$).

* $p < 0.05$ according to the Wilcoxon signed rank test following Friedman test (B and E) and the Mann-Whitney U test (C and F). The data are presented as mean ± SEM. All multiple testing data analyses were followed by Bonferroni correction.

TP: transplantation, BMS: Basso Mouse Scale, CNO: clozapine N-oxide.

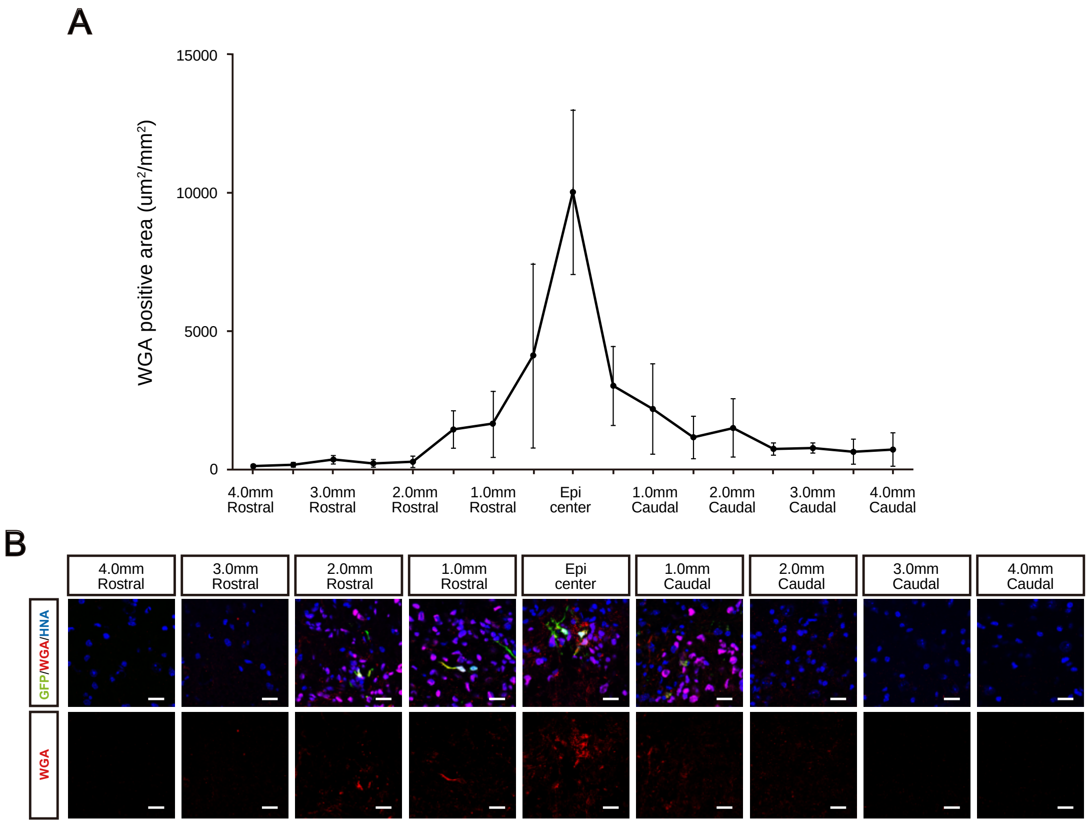

Figure S6. Migrated WGA in the injured spinal cord

(A) Quantitative data of WGA^+/GFP^- areas in different regions (n = 3).

(B) Representative image of migrated WGA at each region. Scale bar, 20 μm.

The data are presented as the mean ± SEM (A).

WGA: Wheat germ agglutinin.

Supplemental Experimental Procedures

Lentiviral vector preparation

Human Synapsin (hSyn), hM4Di and mCherry cDNAs were amplified from pAAV-hSyn-hM4Di-mCherry (addgene #50475) by the polymerase chain reaction (PCR) (1). jGCaMP7f cDNA was amplified from pGP-CMV-jGCaMP7f (addgene #104483). AcGFP1-P2A-WGA cDNA was PCR-amplified from a vector kindly provided by Dr. Yasushi Miyashita (The University of Tokyo, Tokyo, Japan). All cDNAs were transferred into lentiviral vector plasmids: AcGFP1-P2A-WGA cDNA was transferred into the tetracycline(Tet)-inducible lentiviral vector CSIV-TRE-RfA-EF1-Bsd(t), other cDNAs were transferred into the lentiviral vectors CSIV-RfA and CSII-RfA. Recombinant lentiviral vector production was performed as described previously (2). Titer determination was performed by a qRT-PCR based lentiviral titer assay according to the manufacturer's instructions (631235, Cellartis-Takara Bio, Shiga, Japan).

Cell culture and lentiviral transduction

Neural stem/progenitor cells (NS/PCs) were derived as previously reported from a non-tumorigenic clone 414C2 human induced pluripotent stem cells (hiPSCs), that was reprogrammed with episomal plasmid vectors containing six factors (Oct3/4, Sox2, Klf4, L-Myc, LIN28, and p53shRNA) (3-5). The hiPSCs were cultured for 12 days on adhesion culture with mouse embryonic fibroblasts (MEFs). Then, the collected hiPSCs were formed into embryo bodies in floating culture for 30 days. Aggregated cells were dissociated and differentiated into NS/PCs in KBM neural stem cell medium (16050100, Kohjin Bio, Saitama, Japan) containing 2% NeuroBrew-21 (130-093-566, Miltenyi Biotec, North Rhine-Westphalia, Germany), 20 ng/ml human fibroblast growth factor (FGF)-basic (100-18B, Peprotech, Cranbury, NJ, USA) and 10 ng/ml human recombinant leukemia inhibitory factor (hLIF: LIF-1010, Merck Millipore, Burlington, MA, USA). NS/PCs were cultured for 10 to 14 days to allow sphere formation (primary neurospheres). After two passages, the lentiviral vectors were transduced and again cultured as tertiary neurospheres (LV/hSyn-hM4Di-mCherry: hM4Di-NS/PCs, LV/hSyn-mCherry: mCherry-NS/PCs and LV/TRE-AcGFP1-P2A-WGA-EF1-Bsd(t): WGA-NS/PCs).

Neuronal differentiation analysis (*in vitro*)

Dissociated NS/PCs from forth neurospheres were plated onto poly-D lysin/laminin coated 8-well

chamber slides at a density of 1×10^5 cells/well. The cells were cultured in KBM neural stem cell medium containing 2% NeuroBrew-21 at 37°C in 5% CO2 and 95% air for 30 days. Differentiated cells were fixed with 4% paraformaldehyde (PFA) in 0.1 M PBS and stained with the following primary antibodies for immunocytochemical staining: rabbit anti-mCherry (ab167453, Abcam, Cambridge, MA, USA, 1:1000), mouse anti-embryonic lethal abnormal vision-like protein 3/4 (ELAVL 3/4: A21271, Thermo Fisher Scientific, Waltham, MA, USA, 1:200), rat anti-glial fibrillary acidic protein (GFAP: 13-0300, Thermo Fisher Scientific, Waltham, MA, USA, 1:100) and anti-cyclic nucleotide phosphodiesterase (CNPase: C5922, Sigma-Aldrich, St.Louis, MO, USA, 1:2000). Nuclei were stained with Hoechst 33258 (Sigma-Aldrich, St.Louis, MO, USA, 10μg/ml). All *in vitro* images were obtained by confocal laser scanning microscopy (LSM 700, Carl Zeiss, Jena, Germany). Analyses were performed semiautomatically with Zen 2012 SP5 (version 14.0.0.0, Carl Zeiss, Jena, Germany) or a customized macro using ImageJ software(ver. 2.1.0/1.53.c).

Calcium imaging analysis

Tertiary neurospheres of hM4Di-NS/PCs and mCherry-NS/PCs were dissociated and LV/hSyn-jGCaMP7f was transduced. The cells were cultured again as forth neurospheres.

Astrocytes were mechanically dissociated from the cerebral cortices of 3 days old mice (C57B6J, Charles river laboratories Japan, Kanagawa, Japan) and plated on the poly-D lysin coated glass wells. Astrocytes were cultured as feeder cells for 3-7 days at 37°C in 5% CO2 and 95% air. Forth neurospheres of hM4Di-NS/PCs and mCherry-NS/PCs were then dissociated, plated at a density of 2×10^5 cells/well and cultured in Neurobasal Plus Medium (A35829-01, Thermo Fisher Scientific, Waltham, MA, USA) containing: 10 μg/ml Recombinant human brain-derived neurotrophic factor (BDNF: B-250, Alamone labs, Jerusalem, Israel), 10 μg/ml recombinant human glial cell line-derived neurotrophic factor (GDNF: G-240, Alamone labs, Jerusalem, Israel), 100 mM dibutyryladenosine cyclic monophosphate sodium salt (dbcAMP: D0627, Sigma-Aldrich, St.Louis, MO, USA), 200 mM L-ascorbic acid (A8960, Sigma-Aldrich, St.Louis, MO, USA) and 2% B-27 Plus Supplement (A3582801, Thermo Fisher Scientific, Waltham, MA, USA). After culture for 30 days, the cell culture medium was replaced with Hanks' balanced salt solution (HBSS: 14025092, Thermo Fisher Scientific, Waltham, MA, USA) for calcium imaging analysis. Clozapine N-oxide (CNO: BML-NS-105-0025, Enzo Life Sciences, Farmingdale, NY, USA, 10μM of final concentration) was added after 60 seconds of

imaging (total of 120 seconds of observation). Movies were captured at 32 frames/second using an IX83 inverted microscope (Olympus, Tokyo, Japan) equipped with an electron multiplying CCD camera (Hamamatsu Photonics, Shizuoka, Japan) and a pE-4000 LED illumination system (CoolLED, Andover, UK). Data were corrected and analyzed by the miniscope 1-photon-based calcium imaging signal extraction pipeline (6), followed by manual annotation based on identified ROIs and signals using a dedicated GUI written in MATLAB (MathWorks, Inc., MA, USA). Calcium events of detected neurons were identified as the beginning of the rising phase of ΔF/F0 as previously reported (peak ΔF/F0>0.5 standard deviation units from the baseline, derivation of ΔF/F0>2 standard deviation units from the baseline) (7). Normalized calcium event counts after CNO administration for each cell were calculated as follows: Normalized calcium events after CNO administration = calcium events after CNO administration/calcium events before CNO administration (%).

Micro-electrode array

Astrocyte feeder cells were plated on the poly-D lysin coated micro-electrode array (MEA) plates (M768-tMEA-48W, Axion Biosystems, Atlanta, GA, USA). Feeder cells were cultured for 3-7 days at 37°C in 5% CO2 and 95% air. Forth neurospheres of hM4Di-NS/PCs and mCherry-NS/PCs were dissociated, plated at a density of 1×10^5 cells/well and cultured in Neurobasal Plus Medium containing: 10 μg/ml recombinant human BDNF, 10 μg/ml recombinant human GDNF, 100 mM dbcAMP, 200 mM L-ascorbic acid and 2% B-27 Plus Supplement.

All channels were sampled at a rate of 12.5 kHz/channel and filtered using a 200-3000 Hz Butterworth bandpass filter. CNO (10 μM of final concentration) was added after 150 seconds (total of 300 seconds of observation). Spikes were detected at a threshold of 6×SD from the baseline electrode noise. Spikes were counted using the Axion Integrated Studio program (Axion Biosystems, Atlanta, GA, USA). The normalized spike count after CNO administration for each well was calculated as follows: Normalized spike count after CNO administration = spike counts after CNO administration/spike counts before CNO administration (%).

SCI animal model and NS/PC transplantation

Eight weeks old female non-obese diabetic severe combined immunodeficient mice (NOD-SCID, Charles river laboratories Japan, Kanagawa, Japan) were anesthetized with an intraperitoneal

injection of ketamine (100 mg/kg) and xylazine(10 mg/kg). Before making an incision, 12.5 mg/kg ampicillin was administered subcutaneously. Laminectomy was then performed by removing the 10th thoracic spinal vertebral laminae, and the dorsal surface of the dura mater was exposed. Contusive SCI was induced at the 10th level by an Infinite Horizon impactor (70-75 kdyn; Precision Systems and Instrumentation, Lexington, KY, USA), as described previously (8, 9).

7 days after SCI, motor function was evaluated by Basso Mouse Scale (BMS) score. Scores of 2.5 or higher and 1 or lower were excluded from the study. Mice for the neuronal activity inhibition assay (58 mice total) were randomly assigned to one of three groups (hM4Di-TP group, n = 20; mCherry-TP, group n = 18; PBS group, n = 20). Additionally, 18 mice were prepared for the long-term follow up experiment and randomly assigned to two groups (hM4Di-TP group, n = 10; mCherry-TP, group n = 8). 10 mice were prepared for the trans-synaptic tracing assay.

For mice included in the transplantation group, NS/PCs cultured with a small-molecule gamma secretase inhibitor, N-[N-(3,5-difluorophenacetyl)-l-alanyl]-S-phenylglycine t-butyl ester (DAPT: D5942, Sigma-Aldrich, St.Louis, MO, USA, 10 μM) (8), for one day were transplanted into the lesion epicenter of the injury site (5×10^5 cells in 2 μl of PBS, hM4Di-TP group: hM4Di-NS/PCs, mCherry-TP group: mCherry-NS/PCs, trans-synaptic tracing assay group: WGA-NS/PCs) with a Hamilton syringe (87931, Hamilton, Reno, NV, USA) and a 28 G metal needle using a microstereotaxix injection system (KDS310, Muromachi-Kikai Co., Ltd., Tokyo, JAPAN). NS/PCs were injected at a rate of 1 μl/minute, and the syringe was left at the injection site for two minutes after the injection before being removed. Using the same method, 2 μl of PBS was injected into the lesion epicenter of the injury site in PBS group mice.

All experiments were performed in accordance with the Guidelines for the Care and Use of Laboratory Animals of Keio University (Tokyo, Japan, Permit Number; 13020) and NIH Guide for the Care and Use of Laboratory Animals. All surgeries were performed under anesthesia.

Histological analyses

Ten weeks after transplantation, animals were anesthetized and euthanized by transcardial perfusion with 0.1 M PBS containing 4% PFA, followed by sequential soaking overnight in 10% and 30% sucrose. Spinal cord tissues were the embedded in optimal cutting temrerture (O.C.T.) compound (Sakura Finetechnical Co., Ltd., Tokyo, Japan) and sectioned at a thickness of 16 mm for the sagittal

plane and 20 mm for the axial plane.

Spinal cord sections were immunohistochemically stained for histological analysis using the following primary antibodies; mouse anti-human nuclear antigen (HNA: MAB4383, Merck Millipore, Burlington, MA, USA, 1:400), rabbit anti-mCherry (ab167453, 1:1000), mouse anti-ELAVL 3/4 (A21271, 1:200), rabbit anti-GFAP (16825-1-AP, Proteintech, Rosemont, IL, USA, 1:4000), mouse anti-adenomatous poluposis coli CC-1 (APC: OP80, Merck Millipore, Burlington, MA, USA, 1:400), rabbit anti-Nestin (18741, Immuno-Biological Laboratories, Gunma, Japan, 1:400), rabbit anit-Ki67 (NCL-Ki67p, Leica Biosystems, Buffalo Grove, IL, USA, 1:2000), mouse anti-synaptophysin (MAB329, Merck Millipore, Burlington, MA, USA, 1:10000), mouse anti-Gephyrin(1470011, Synaptic Systems, Goettingen, Germany, 1:1000), anti-post synaptic density 95 (PSD95: 51-6900, Thermo Fisher Scientific, Waltham, MA, USA, 1:100), mouse anti-human cytoplasm antibody (STEM121: Y40420, Cellartis-Takara Bio, Shiga, Japan, 1:200), rabbit anti-wheat germ agglutinin (WGA: T4144, Thermo Fisher Scientific, Waltham, MA, USA, 1:20000), goat anti-GFP (600-101-215, Rockland Immunochemicals, Pottstown, PA, USA, 1:1000), goat anti-choline acetyltransferase antibody (ChAT: AB144P, Merck Millipore, Burlington, MA, USA, 1:200). Nuclei were stained with Hoechst 33258 (10µg/ml). Sample images were obtained using a fluorescence microscope (BZ 9000, Keyence Co., Osaka, Japan) or a confocal laser scanning microscopy. Analyses were performed in combination with Zen 2012 SP5 and a customized macro using ImageJ software.

Immunoelectron microscopy analysis

Briefly, spinal cord tissues were prepared as mentioned above by perfusion and by postfixation with 4% PFA, followed by the cryoprotective treatment with 15% and 30% sucrose, embedding and freezing into a cryomold with Frozen Section Compound (FSC22, Leica Microsystems, Germany). Frozen sections were prepared at 20 µm thickness with a cryostat (CM3050s, Leica Microsystems, Germany). Sections were incubated with 5% Blockace (DS Pharma Biomedical, Osaka, Japan) supplemented with 0.01% Saponin in 0.1 M PB for an hour. The sections were stained with a rabbit anti-mCherry (ab167453, 1:200) primary antibody for 72 hours at 4°C and incubated with a FluoroNanogold-conjugated goat anti-rabbit secondary antibody (A24922, Thermo Fisher Scientific, Waltham, MA, USA, 1:100) for 24 hours at 4°C. After 2.5% glutaraldehyde fixation, nanogold signals were enhanced with Silver Enhancement solution for seven minutes at 25°C. The sections were

postfixed with 1.0% Osmium Tetroxide for 90 minutes at 4°C, *en bloc* stained with uranyl acetate for 20 minutes at 4°C, dehydrated through a graded ethanol series, and embedded into pure Epon. Ultrathin sections (80 nm) were prepared with an ultramicrotome (UC7, Leica Microsystems, Germany) and electron stained with uranyl acetate and lead citrate. Ultrathin sections were finally observed under a transmission electron microscope (JEM1400 plus, JEOL, Tokyo, Japan).

Behavioral analyses

Investigators blinded to identify the experimental groups performed this assessment. Hind limb locomotor function was evaluated using the BMS scoring system (10) on a weekly basis. The gait analyses were performed on a treadmill (DigiGait system, Mouse Specifics, Framingham, MA, USA) at the final follow-up point. The stride length and stance angle were determined on a treadmill set to a speed of 7 cm/second (8).

Neural activity inhibiting assay (*in vivo*)

BMS scores were evaluated as a baseline function (8-10). CNO (10mg/kg) dissolved in saline was intraperitoneally administered to all animals (11, 12). Functional analyses were performed again at 1 and 24 hours after CNO administration. BMS scoring of mice with neuronal activity inhibition was performed at 28 days after SCI and at the final follow-up. Treadmill gait analysis of mice with neural activity inhibition was performed at the final follow-up for mice with BMS scores of three or more on either side of the hind limb in the hM4Di-TP and mCherry-TP groups. The administration of CNO and the functional analysis were performed at time points similar to those used for BMS scoring.

Electrophysiology

Electrophysiological experiments were performed using a Neuropack S1 MEB9402 signal processor (Nihon Kohden, Tokyo, Japan) at 18 weeks post injury as previously described (13, 14). Mice were anesthetized by intraperitoneal injections of three types mixed anesthesia (5 mg/kg butorphanol, 0.75 mg/kg medetomidine, and 4 mg/kg midazolam). The spinal cord at the Th5 level was exposed and stimulated by a nichrome wire tied to artificial dura mater that was placed on the exposed spinal cord. The signal in the hindlimb was detected by wire electrodes (gage 0.0508 mm, nichrome). The active electrode was placed in the quadriceps muscle belly, the reference electrode was placed near the

distal quadriceps tendon of the muscle, and the ground electrode was placed in the tail. Stimulation with an intensity of 1.0–3.0 mA, 1-3 train, duration of 0.2 ms, and interstimulus interval of 1 Hz was used. An average of 20 MEP wave was aggregated as a representative wave. The maximal amplitude was measured from the lowest point to the highest point of the wave. Peak latency was measured as the length of time from the stimulation to the highest point of the MEP wave. After the first evaluation, 10mg/kg CNO was administered under anesthesia. MEP wave was re-evaluated 30 minutes after CNO administration. Post-CNO measurements were normalized as follows: Normalized measurement = post-CNO measurement / pre-CNO measurement.

Induction of doxycycline

Mice allocated to the trans-synaptic tracing assay group were fed an autoclaved rodent diet supplemented with 0.0625% doxycycline(DOX: D13110903, Research Diets, New Brunswick, NJ, USA) beginning at 56 days after SCI for a period of 14 days (15).

Supplemental References

1. Roth BL. DREADDs for Neuroscientists. Neuron. 2016;89(4):683-94.

2. Miyoshi H, Blomer U, Takahashi M, Gage FH, Verma IM. Development of a self-inactivating lentivirus vector. J Virol. 1998;72(10):8150-7.

3. Okada Y, Matsumoto A, Shimazaki T, Enoki R, Koizumi A, Ishii S, et al. Spatiotemporal recapitulation of central nervous system development by murine embryonic stem cell-derived neural stem/progenitor cells. Stem Cells. 2008;26(12):3086-98.

4. Nori S, Okada Y, Yasuda A, Tsuji O, Takahashi Y, Kobayashi Y, et al. Grafted human-induced pluripotent stem-cell-derived neurospheres promote motor functional recovery after spinal cord injury in mice. Proc Natl Acad Sci U S A. 2011;108(40):16825-30.

5. Okubo T, Nagoshi N, Kohyama J, Tsuji O, Shinozaki M, Shibata S, et al. Treatment with a Gamma-Secretase Inhibitor Promotes Functional Recovery in Human iPSC- Derived Transplants for Chronic Spinal Cord Injury. Stem Cell Reports. 2018;11(6):1416-32.

6. Lu J, Li C, Singh-Alvarado J, Zhou ZC, Frohlich F, Mooney R, et al. MIN1PIPE: A Miniscope 1-Photon-Based Calcium Imaging Signal Extraction Pipeline. Cell Rep. 2018;23(12):3673-84.

7. Nemoto A, Kobayashi R, Yoshimatsu S, Sato Y, Kondo T, Yoo AS, et al. Direct Neuronal

Reprogramming of Common Marmoset Fibroblasts by ASCL1, microRNA-9/9*, and microRNA-124 Overexpression. Cells. 2020;10(1).
8. Okubo T, Iwanami A, Kohyama J, Itakura G, Kawabata S, Nishiyama Y, et al. Pretreatment with a gamma-Secretase Inhibitor Prevents Tumor-like Overgrowth in Human iPSC-Derived Transplants for Spinal Cord Injury. Stem Cell Reports. 2016;7(4):649-63.
9. Kamata Y, Isoda M, Sanosaka T, Shibata R, Ito S, Okubo T, et al. A robust culture system to generate neural progenitors with gliogenic competence from clinically relevant induced pluripotent stem cells for treatment of spinal cord injury. Stem Cells Transl Med. 2021;10(3):398-413.
10. Basso DM, Fisher LC, Anderson AJ, Jakeman LB, McTigue DM, Popovich PG. Basso Mouse Scale for locomotion detects differences in recovery after spinal cord injury in five common mouse strains. J Neurotrauma. 2006;23(5):635-59.
11. Dell'Anno MT, Wang X, Onorati M, Li M, Talpo F, Sekine Y, et al. Human neuroepithelial stem cell regional specificity enables spinal cord repair through a relay circuit. Nat Commun. 2018;9(1):3419.
12. Jendryka M, Palchaudhuri M, Ursu D, van der Veen B, Liss B, Katzel D, et al. Pharmacokinetic and pharmacodynamic actions of clozapine-N-oxide, clozapine, and compound 21 in DREADD-based chemogenetics in mice. Sci Rep. 2019;9(1):4522.
13. Tashiro S, Nishimura S, Iwai H, Sugai K, Zhang L, Shinozaki M, et al. Functional Recovery from Neural Stem/Progenitor Cell Transplantation Combined with Treadmill Training in Mice with Chronic Spinal Cord Injury. Sci Rep. 2016;6:30898.
14. Ito S, Nagoshi N, Tsuji O, Shibata S, Shinozaki M, Kawabata S, et al. LOTUS Inhibits Neuronal Apoptosis and Promotes Tract Regeneration in Contusive Spinal Cord Injury Model Mice. eNeuro. 2018;5(5).
15. Kojima K, Miyoshi H, Nagoshi N, Kohyama J, Itakura G, Kawabata S, et al. Selective Ablation of Tumorigenic Cells Following Human Induced Pluripotent Stem Cell-Derived Neural Stem/Progenitor Cell Transplantation in Spinal Cord Injury. Stem Cells Transl Med. 2019;8(3):260-70.
