# Supplementary material for: Modulation by DREADD reveals the therapeutic effect of human iPSC-derived neuronal activity on functional recovery after spinal cord injury
Source: Stem Cell Reports. 2022 Jan 11;17(1):127–42. doi: 10.1016/j.stemcr.2021.12.005 (PMC8758967; doi:10.1016/j.stemcr.2021.12.005)
Supplement: Document S2. Article plus supplemental information [file mmc4.pdf]

Stem Cell Reports
Article
OPEN ACCESS

Modulation by DREADD reveals the therapeutic effect of human iPSC-derived neuronal activity on functional recovery after spinal cord injury

Takahiro Kitagawa,[1,2] Narihito Nagoshi,[1,*] Yasuhiro Kamata,[1,2] Momotaro Kawai,[1,2] Kentaro Ago,[1,2] Keita Kajikawa,[1,2] Reo Shibata,[1,2] Yuta Sato,[3,4] Kent Imaizumi,[2] Tomoko Shindo,[5] Munehisa Shinozaki,[2] Jun Kohyama,[2] Shinsuke Shibata,[5,6] Morio Matsumoto,[1] Masaya Nakamura,[1] and Hideyuki Okano[2,*]
[1]Department of Orthopaedic Surgery, Keio University School of Medicine, 35 Shinanomachi, Shinjuku-ku, Tokyo 160-8582, Japan
[2]Department of Physiology, Keio University School of Medicine, 35 Shinanomachi, Shinjuku-ku, Tokyo 160-8582, Japan
[3]Graduate School of Science and Technology, Keio University, 3-14-1 Hiyoshi, Kohoku-ku, Yokohama City, Kanagawa 223-8522, Japan
[4]Laboratory for Marmoset Neural Architecture, RIKEN Center for Brain Science, 2-1 Hirosawa, Wako City, Saitama 351-0198, Japan
[5]Electron Microscope Laboratory, Keio University School of Medicine, 35 Shinanomachi, Shinjuku-ku, Tokyo 160-8582, Japan
[6]Division of Microscopic Anatomy, Graduate School of Medical and Dental Sciences, Niigata University, 1-757 Asahimachi-dori, Chuo-ku, Niigata City, Niigata 951-8510, Japan
*Correspondence: nagoshi@2002.jukuin.keio.ac.jp (N.N.), hidokano@a2.keio.jp (H.O.)
https://doi.org/10.1016/j.stemcr.2021.12.005

SUMMARY

Transplantation of neural stem/progenitor cells (NS/PCs) derived from human induced pluripotent stem cells (hiPSCs) is considered to be a promising therapy for spinal cord injury (SCI) and will soon be translated to the clinical phase. However, how grafted neuronal activity influences functional recovery has not been fully elucidated. Here, we show the locomotor functional changes caused by inhibiting the neuronal activity of grafted cells using a designer receptor exclusively activated by designer drugs (DREADD). *In vitro* analyses of inhibitory DREADD (hM4Di)-expressing cells demonstrated the precise inhibition of neuronal activity via administration of clozapine N-oxide. This inhibition led to a significant decrease in locomotor function in SCI mice with cell transplantation, which was exclusively observed following the maturation of grafted neurons. Furthermore, trans-synaptic tracing revealed the integration of graft neurons into the host motor circuitry. These results highlight the significance of engrafting functionally competent neurons by hiPSC-NS/PC transplantation for sufficient recovery from SCI.

INTRODUCTION

Stem cell-based approaches have been reported to be an effective therapy for spinal cord injury (SCI) (Cummings et al., 2005; Kobayashi et al., 2012; Nori et al., 2011). Previous reports from our group and others have demonstrated the efficacy of transplanting neural stem/progenitor cells (NS/PCs) derived from human induced pluripotent stem cells (hiPSCs), which are considered a promising cell source with limited ethical concerns (Fujimoto et al., 2012; Kobayashi et al., 2012; Nori et al., 2011; Okubo et al., 2016). Several beneficial factors of NS/PC transplantation have been proposed, including axonal regeneration with synaptic formation, remyelination, and neuroprotection by trophic factors (Assinck et al., 2017; Nori et al., 2017). Among these mechanisms, axonal regeneration is of great significance due to the characteristics of NS/PCs, which provide neuronal cells that integrate into host tissue and reconnect neuronal networks (Abematsu et al., 2010; Bonner et al., 2011; Bonner and Steward, 2015; Lu et al., 2012).

A number of previous studies emphasized the synaptic connection between graft and host neurons and implied the necessity of integrating graft neurons into host neuronal circuits (Ceto et al., 2020; Kadoya et al., 2016; Lu et al., 2012, 2019). For instance, our previous study transplanting neurogenic NS/PCs led to a further functional improvement of host animals, which suggested the potency of robust neuronal differentiation (Okubo et al., 2016). However, our studies and others advocated the importance of neuronal connectivity mainly by histological analyses, and there was a lack of direct proof displaying the relationship between neuronal activity in grafted neurons and locomotor outcomes. Definitive evidence of the therapeutic mechanism mediated by integrated graft-derived neurons is crucial to promote hiPSC-NS/PC transplantation therapy for SCI, which is now on the road toward clinical trials (Nagoshi et al., 2020; Tsuji et al., 2019).

Several approaches can be utilized to demonstrate how the transplanted cells actually contribute to the improvement of motor function. Administration of diphtheria toxin is frequently used to ablate engrafted human-derived cells (Abematsu et al., 2010; Cummings et al., 2005; Fujimoto et al., 2012). However, this methodology eliminates all the grafted cells and cannot assess the function of engrafted neurons. To compensate for this limitation, designer receptors exclusively activated by designer drugs (DREADD) have attracted attention for their ability to specifically control neuronal activity (Nichols and Roth, 2009; Roth, 2016). This is one of the chemogenetically engineered proteins that permit control of G protein signaling by administering the ligand clozapine N-oxide (CNO) and

This is an open access article under the CC BY license (http://creativecommons.org/licenses/by/4.0/).

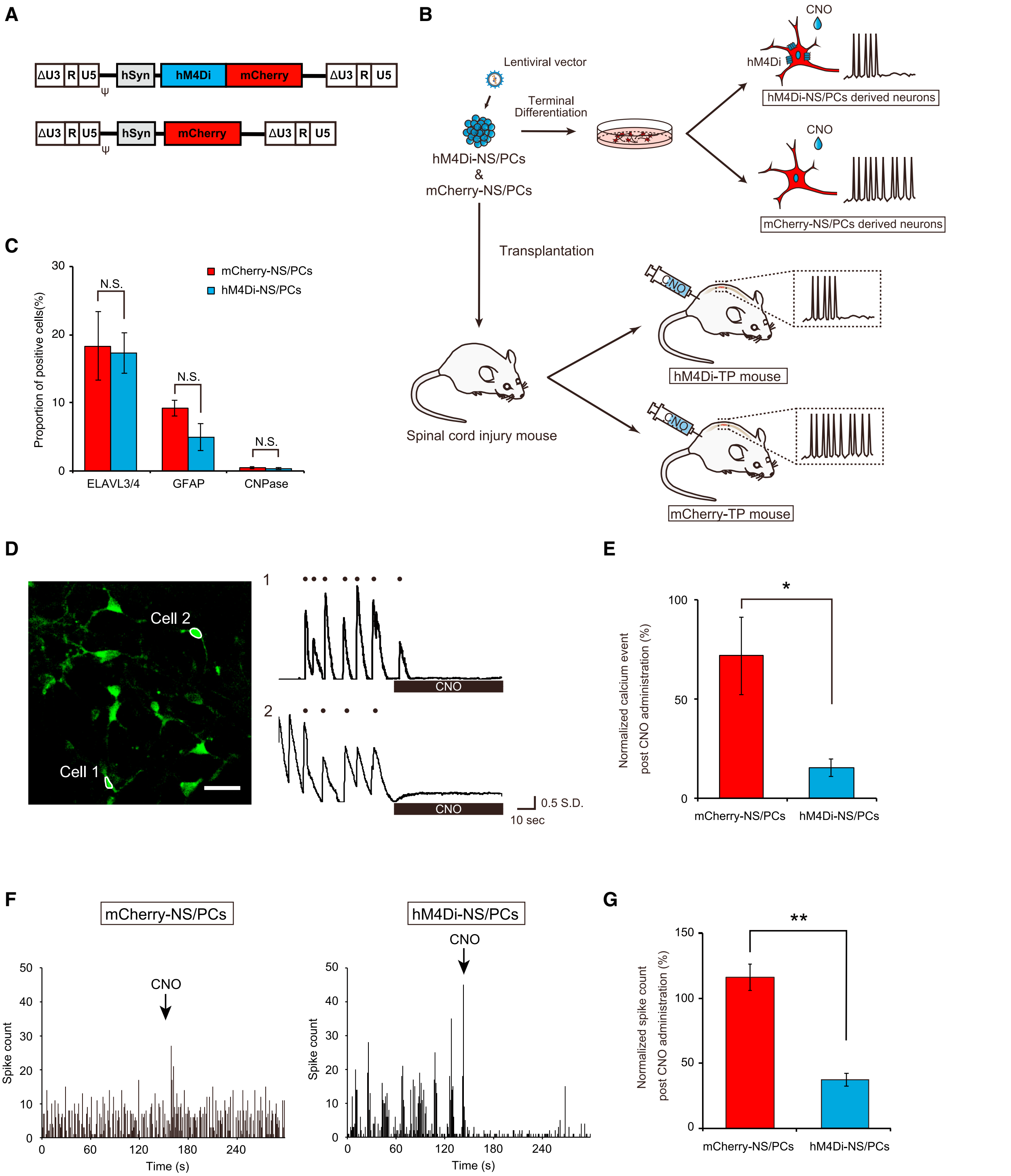

Figure 1. Establishment of hM4Di-expressing NS/PCs; differential potential and inhibitory functional assessments *in vitro*

(A) Schematic of the integrated proviral form of the lentiviral vectors. Top: CSIV-hSyn-hM4Di-mCherry. Bottom: CSIV-hSyn-mCherry.

(B) Schematic of the experiments.

(C) Percentage of cell-type-specific marker cells in differentiated cells of mCherry-NS/PCs and hM4Di-NS/PCs (mCherry-NS/PCs, n = 4 wells/2 independent experiments; hM4Di-NS/PCs, n = 4 wells/2 independent experiments).

(legend continued on next page)

manipulating neuronal activity (Aldrin-Kirk et al., 2016; Nichols and Roth, 2009; Roth, 2016). Several studies have conducted cell transplantation research using the DREADD system for central nervous system disorders (Aldrin-Kirk et al., 2016; Chen et al., 2016; Zou et al., 2016). In a model of Parkinson's disease, functional decline in host animals induced by the selective inhibition of transplanted dopamine neurons indicated the therapeutic efficacy of cell replacement therapy (Chen et al., 2016). In a previous study on SCI, one study transplanted neuroepithelial stem cells derived from embryonic postmortem specimens and assessed the impact of graft neuronal inhibition on locomotor function (Dell'Anno et al., 2018). However, this report had the following limitations that prohibited complete elucidation of the role of graft-derived neuronal function: limited functional recovery by the cell replacement, inadequate control animals that did not fully eliminate the possibility of off-target effects by the DREADD system, and no confirmation that the grafted neurons were connected to the host motor circuits. Most importantly, no previous study has revealed the direct contribution of neuronal activity in grafted hiPSC-NS/PCs using an SCI animal model.

The present study aimed to evaluate the association of graft neuronal activity and host locomotor function in hiPSC-derived NS/PC transplantation by controlling the neuronal activity using the inhibitory DREADD receptor hM4Di (Nichols and Roth, 2009). Adding a technique of trans-synaptic tracing, the current study rigorously clarified the mechanisms of cell transplantation therapy in SCI, especially focusing on the neuronal function.

RESULTS

The activity of hM4Di-expressing neurons derived from hiPSC-NS/PCs was inhibited by administration of CNO

We used lentiviral vectors to label NS/PCs, which derived neurons expressing hM4Di fused with mCherry (hM4Di-NS/PCs) and neurons expressing only mCherry as a negative control (mCherry-NS/PCs) (Figures 1A and 1B). The expression of these genes was controlled by the human Synapsin (hSyn) promoter (Egashira et al., 2018).

The differential potentials of the two cell lines were not different as determined by immunocytochemical (ICC) analysis, which yielded the following results: embryonic lethal abnormal vision-like protein 3/4 (ELAVL3/4)$^{+}$ neurons (17.3% ± 3.0% versus 18.4% ± 5.0%, $p = 1.000$), glial fibrillary acidic protein (GFAP)$^{+}$ astrocytes (4.9% ± 2.0% versus 9.3% ± 1.1%, $p = 0.400$), and cyclic nucleotide phosphodiesterase (CNPase)$^{+}$ oligodendrocytes (0.4% ± 0.0% versus 0.4% ± 0.1%, $p = 0.700$) (Figures 1C, S1A, and S1B).

To test the functionality of hM4Di in neurons, we co-transfected a lentiviral vector encoding the Janelia green fluorescent protein-calmodulin fusion protein (jGCaMP7f) gene (Dana et al., 2019), which is a genetically encoded calcium indicator, under the control of the hSyn promoter into hM4Di-NS/PCs and mCherry-NS/PCs (Figure S2A). The NS/PCs were differentiated to obtain neurons, followed by 30 days of culture for maturation. The matured neurons exhibited sporadic calcium events, indicating spontaneous neural activities (Figures 1D and S2B, Videos S1, and S2). The addition of CNO significantly decreased the number of calcium events in neurons differentiated from hM4Di-NS/PCs (5.6 ± 0.8 events/min versus 1.1 ± 0.3 events/min, $p < 0.001$), whereas that in neurons differentiated from mCherry-NS/PCs did not differ significantly from the base line (4.9 ± 0.7 events/min versus 3.5 ± 1.1 events/min, $p = 0.053$) (Figure S2C). The normalized number of calcium events after CNO administration was significantly lower in hM4Di-expressing neurons than in the negative control (15.5% ± 4.4% versus 71.5% ± 19.3%, $p = 0.016$) (Figure 1E).

To further confirm the electrophysiological activity in the cell population, a micro-electrode array (MEA) analysis was performed (Okubo et al., 2016; Sato et al., 2021). After 30 days of culture to allow neuronal maturation, neurons from both cell lines showed spontaneous activity before CNO administration (Figure 1F). The neuronal activity of cells differentiated from hM4Di-NS/PCs showed clear inhibition following the administration of CNO, whereas that of cells differentiated from mCherry-NS/PCs was not altered. The normalized spike

(D) Representative calcium transients of neurons differentiated from hM4Di-NS/PCs. Left: an image of neurons expressing jGCaMP7f in a single field of view. Scale bar, 30 μm. Right: time traces of representative cells highlighted with white outlines in the left image (the black dot indicates the calcium event).
(E) Average of normalized calcium events after CNO administration for neurons differentiated from mCherry-NS/PCs and hM4Di-NS/PCs (mCherry-NS/PCs, n = 15 cells/3 independent experiments; hM4Di-NS/PCs, n = 18 cells/3 independent experiments).
(F) Evaluation of neural activity by multi-electrode array assay. Representative time traces of the spike count by CNO administration (left, mCherry-NS/PCs; right, hM4Di-NS/PCs).
(G) Average normalized spike counts after CNO administration of neurons differentiated from mCherry-NS/PCs and hM4Di-NS/PCs (mCherry-NS/PCs, n = 10 wells/2 independent experiments; hM4Di-NS/PCs, n = 10 wells/2 independent experiments). *$p < 0.05$, **$p < 0.01$, and N.S., not significant according to the Mann-Whitney U test (C, E, and G). The data are presented as the mean ± SEM.

counts after CNO administration revealed that neuronal activity was significantly inhibited by CNO administration in differentiated cells from hM4Di-NS/PCs (37.2% ± 5.1% versus 116.0% ± 10.0%, $p < 0.001$) (Figure 1G). Together, these results show that we generated hM4Di-expressing neurons from hiPSC-NS/PCs that were well modulated by CNO administration.

Engrafted NS/PCs differentiated into neural cells in the injured spinal cord

We transplanted hM4Di-NS/PCs (hM4Di-TP group) and mCherry-NS/PCs (mCherry-TP group) into SCI mice at the subacute phase (Nori et al., 2011; Okubo et al., 2016). Histological analyses were performed to evaluate the characteristics of the engrafted cells at 10 weeks after transplantation.

mCherry-positive fibers showed graft-derived neuronal axons that extended caudally and rostrally from the engrafted site (Figure 2A). The neural differentiation and maturation of the engrafted cells were evaluated by double staining with human nuclear antigen (HNA) and cell-type-specific markers (Figures 2B and 2D). In both groups, the quantified proportions did not differ for ELAVL3/4^{+} neurons (54.5% ± 3.9% versus 51.6% ± 3.1%, $p = 0.699$), GFAP^{+} astrocytes (10.2% ± 0.6% versus 9.6% ± 1.6%, $p = 0.589$), or adenomatous polyposis coli CC-1 (APC)$^{+}$ oligodendrocytes (10.8% ± 2.4% versus 11.9% ± 2.5%, $p = 0.699$) (Figure 2C). Furthermore, no differences were observed in the number of immature cells quantified by the neural stem cell marker Nestin (4.4% ± 0.8% versus 5.0% ± 1.0%, $p = 0.589$) or the cell proliferation marker Ki67 (2.3% ± 0.3% versus 2.3% ± 0.1%, $p = 0.699$) (Figure 2E). In addition to the immunohistochemical (IHC) features, no tumor-like tissue formation of engrafted cells was observed by hematoxylin and eosin (H&E) staining (Figures S3A and S3B). These results demonstrated that transplanted NS/PCs dominantly differentiated into mature neurons and did not show different differentiation characteristics or tumorigenic changes that could be affected by the induction of each lentiviral vector.

Neurons differentiated from the grafted NS/PCs connected to host neurons by synaptic formation

To determine the ability of the transplanted neurons to integrate into host neuronal circuitry, we histologically assessed the synaptic formation of graft neurons. IHC staining of mCherry demonstrated that the descending graft fibers penetrated into the gray matter from the white matter (Figures 3A and 3B). The presynaptic boutons of grafted cells colocalized with both the excitatory postsynapse button marker postsynaptic density protein 95 (PSD95) and the inhibitory postsynapse button marker gephyrin (Figures 3C and 3D). Quantitative analysis revealed the inhibitory dominant proportion of graft-to-host synaptic connections (PSD95-positive synapse, 26.5% ± 6.6% versus 27.8% ± 5.8%, $p = 1.000$; gephyrin-positive synapse, 49.5% ± 3.1% versus 45.8% ± 3.5%, $p = 0.400$) (Figure 3E).

Additional evaluation of synaptic formation was performed by immunoelectron microscopy analysis. Connections suggesting excitatory and inhibitory synapses were confirmed in both grafted-to-host neurons and grafted-to-grafted neurons (Figures 3F and 3G) (Gray, 1959).

Transplanted NS/PCs enhanced functional recovery following SCI

To evaluate the therapeutic effect of NS/PC transplantation, recovery of locomotor function was assessed by the Basso mouse scale (BMS) and treadmill gait analysis (Basso et al., 2006; Okubo et al., 2016).

The BMS score was significantly improved by NS/PC transplantation, whereas no significant difference in the BMS score was detected between the two transplanted groups (repeated two-way ANOVA, $p = 0.007$; *post hoc* hM4Di-TP versus PBS, adjusted $p = 0.003$; mCherry-TP versus PBS, adjusted $p = 0.027$; hM4Di-TP versus mCherry-TP, adjusted $p = 1.000$) (Figure 4A). In treadmill gait analysis performed at 9 weeks after transplantation, animals in the transplanted groups showed significantly narrower paw angles and longer stride lengths than those in the non-transplanted group (paw angle: hM4Di-TP, 24.3° ± 2.7°; mCherry-TP, 26.9° ± 3.4°; PBS, 46.1° ± 4.4°; Kruskal-Wallis test, $p < 0.001$; *post hoc* hM4Di-TP versus PBS, adjusted $p < 0.001$; mCherry-TP versus PBS, adjusted $p = 0.006$; hM4Di-TP versus mCherry-TP, adjusted $p = 1.000$; stride length: hM4Di-TP, 4.0 ± 0.2 cm; mCherry-TP, 3.9 ± 0.2 cm; PBS, 2.9 ± 0.2 cm; Kruskal-Wallis test, $p = 0.002$; *post hoc* hM4Di-TP versus PBS, adjusted $p = 0.003$; mCherry-TP versus PBS, adjusted $p = 0.012$; hM4Di-TP versus mCherry-TP, adjusted $p = 1.000$) (Figures 4B and 4C). In contrast, neither the paw angle nor the stride length differed between the hM4Di-TP group and the mCherry-TP groups. From these results, transplanting NS/PCs enhanced locomotor functional recovery after SCI. In addition, there was no difference in functional recovery after hM4Di-NS/PC and mCherry-NS/PC transplantation.

The locomotor function of hM4Di-NS/PC-transplanted mice was controlled by CNO administration

Finally, we evaluated the effect of transplanted neurons on the locomotor function of host mice. Functional behavior was assessed before (pre-CNO), 1 h after (post-CNO), and 24 h after (washout) CNO administration. The BMS score with CNO administration was assessed at 3 and 9 weeks

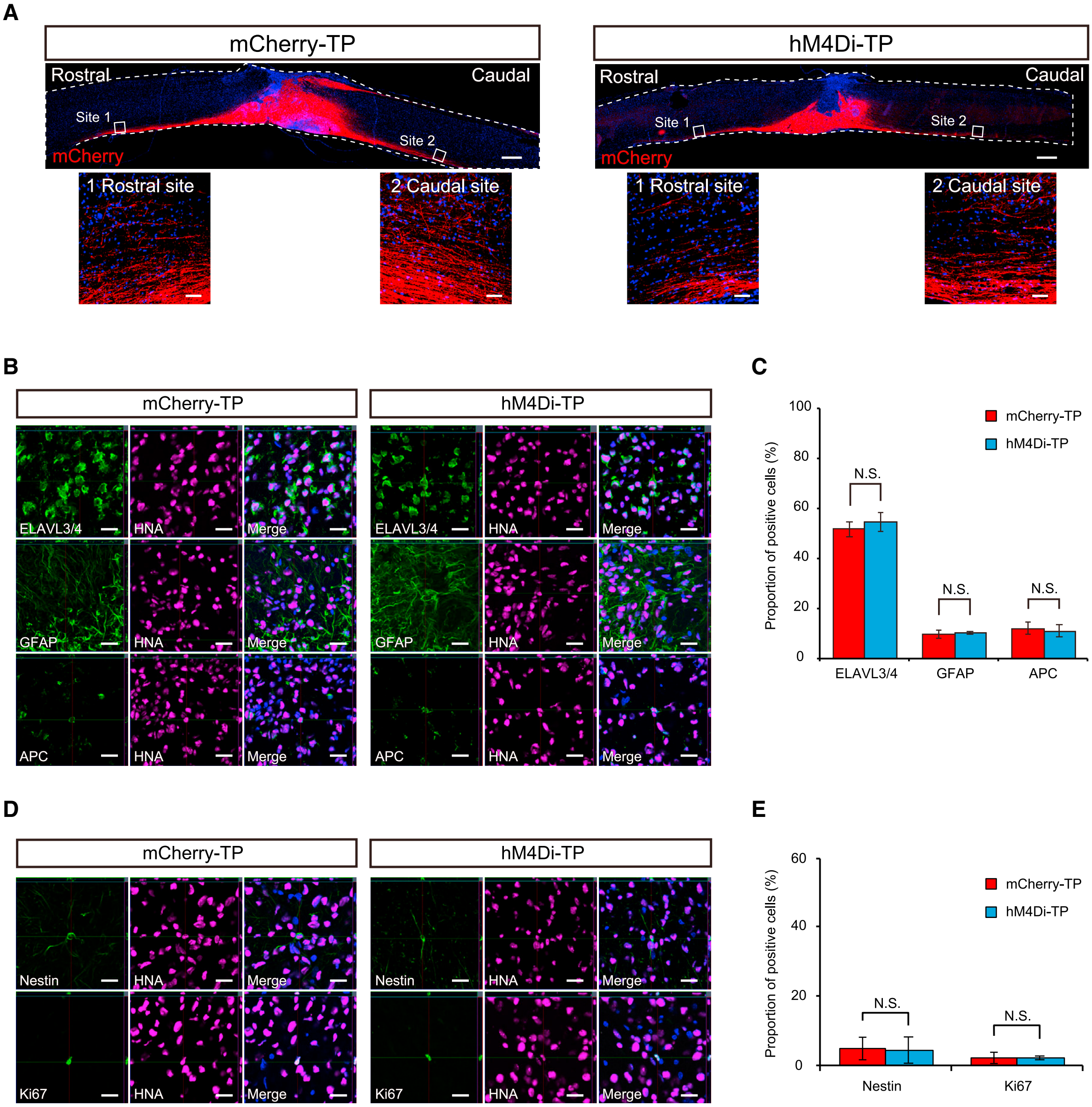

Figure 2. Histological appearance of a spinal cord with transplanted hiPSC-NS/PCs

(A) Representative image of sagittal sections stained for mCherry (left, mCherry-TP; right, hM4Di-TP). mCherry-positive engrafted neurons were elongated rostrally and caudally. Scale bars, 500 and 50 μm (enlarged images).

(B) Representative images of neural cells differentiated from graft cells of the mCherry-TP group (left) and hM4Di-TP group (right). HNA (human cell)-positive engrafted cells merged with ELAVL3/4 (neurons), GFAP (astrocytes), and APC (oligodendrocytes) in each transplanted group. Scale bars, 20 μm.

(C) Histograms showing the quantification of three engrafted neural lineage cells in each transplanted group (mCherry-TP group, n = 6; hM4Di-TP group, n = 6).

(D) Representative images of immature graft cells in the mCherry-TP group (left) and hM4Di-TP group (right). HNA (human cell)-positive engrafted cells merged with Nestin (immature cell) and Ki67 (immature cell) in each transplanted group. Scale bars, 20 μm.

(E) Histograms showing the quantification of immature cells in engrafted cells (mCherry-TP group, n = 6; hM4Di-TP group, n = 6). Not significant (N.S.) according to the Mann-Whitney U test (C and E). The data are presented as the mean ± SEM.

after transplantation. Treadmill gait analysis with CNO administration was performed at 9 weeks after transplantation (Figure 5A).

After administration of CNO at 3 weeks after transplantation, no functional changes were observed in the hM4Di-TP group or mCherry-TP group (mCherry-TP: pre-CNO,

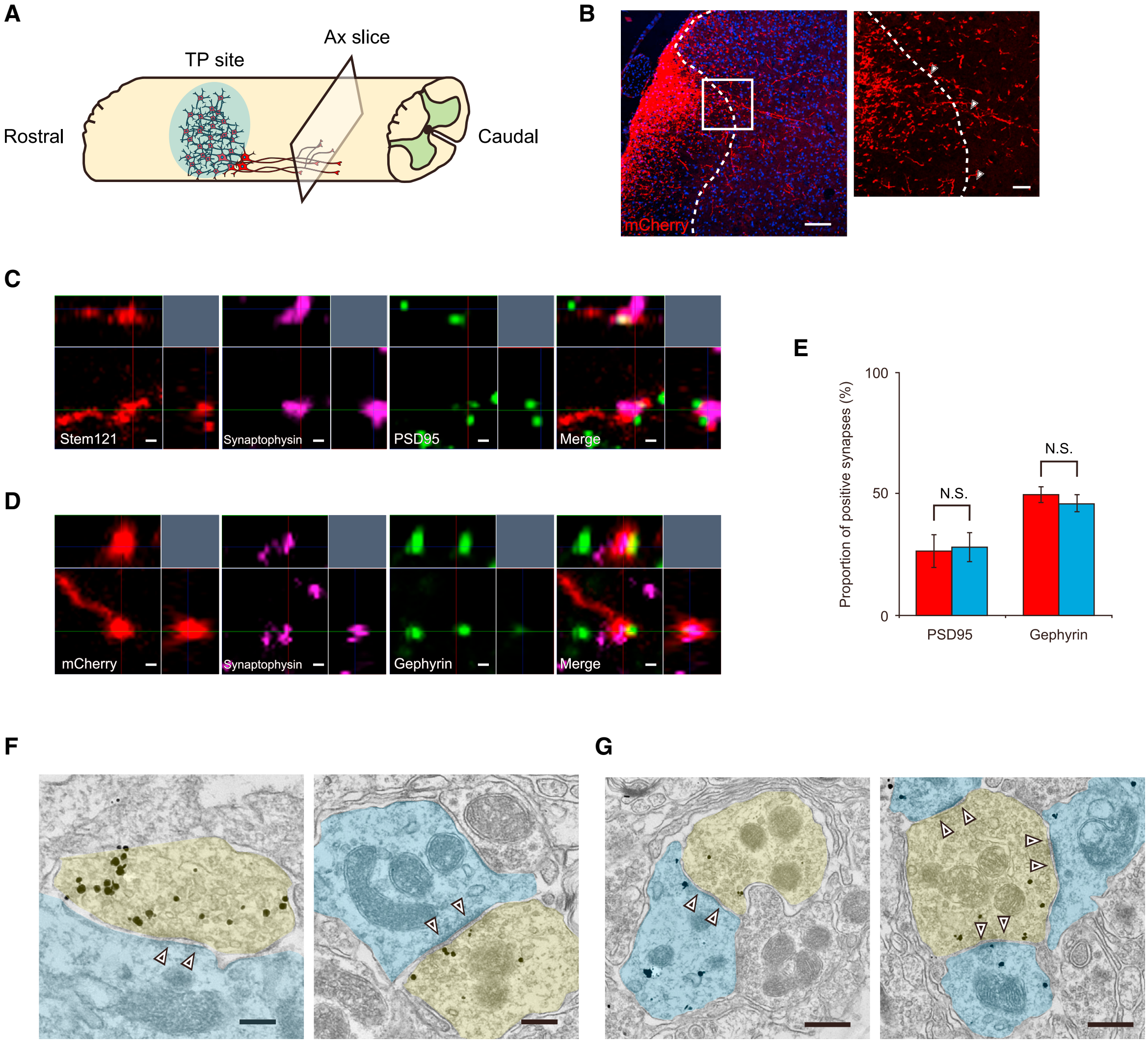

Figure 3. Synaptic formation of neurons derived from transplanted NS/PCs

(A and B) Schema (A) and representative image (B) of axial sections 4 mm caudal to the injury site stained for mCherry. mCherry-positive fibers elongated into the gray matter (arrowheads). Scale bars, 100 and 20 μm (enlarged image).

(C) High-magnification view of excitatory synaptic formation. The presynaptic marker synaptophysin merged with Stem121 and adjacent to the excitatory postsynaptic marker postsynaptic density 95. Scale bars, 0.5 μm.

(D) High-magnification view of inhibitory synaptic formation. The presynaptic marker synaptophysin merged with mCherry and adjacent to the inhibitory postsynaptic marker gephyrin. Scale bars, 0.5 μm.

(E) Histograms showing the quantification of excitatory ($PSD95^{+}$) and inhibitory ($gephyrin^{+}$) synapses in each transplanted group (mCherry-TP group, n = 3; hM4Di-TP group, n = 3).

(F) Immunoelectron microscopy image of a synaptic connection (white arrowheads) from immunogold-labeled mCherry-positive graft neuron (yellow) to host neuron (light blue). Symmetrical connection (left) and asymmetrical connection (right) were observed. Scale bars, 200 nm.

(G) Immunoelectron microscopy image of a synaptic connection (white arrowhead) between two graft neurons. The presynaptic button containing synaptic vesicles (yellow) and postsynaptic button without vesicles (light blue) are both immunogold labeled with mCherry. Symmetrical connection (left) and asymmetrical connection (right) were observed. Scale bars, 500 nm. Not significant (N.S.) according to the Mann-Whitney U test (E). The data are presented as the mean ± SEM.

3.2 ± 0.2; post-CNO, 3.1 ± 0.2; washout, 3.2 ± 0.3, Friedman test, p = 0.651; hM4Di-TP: pre-CNO, 3.1 ± 0.2; post-CNO, 3.1 ± 0.2; washout, 3.0 ± 0.2, Friedman test, p = 0.468) (Figure 5B). In contrast, at 9 weeks after transplantation, the hM4Di-TP group showed a significantly lower BMS score after administration of CNO, whereas the score in the mCherry-TP group was not significantly different (mCherry-TP: pre-CNO, 3.2 ± 0.3; post-CNO, 3.1 ± 0.3;

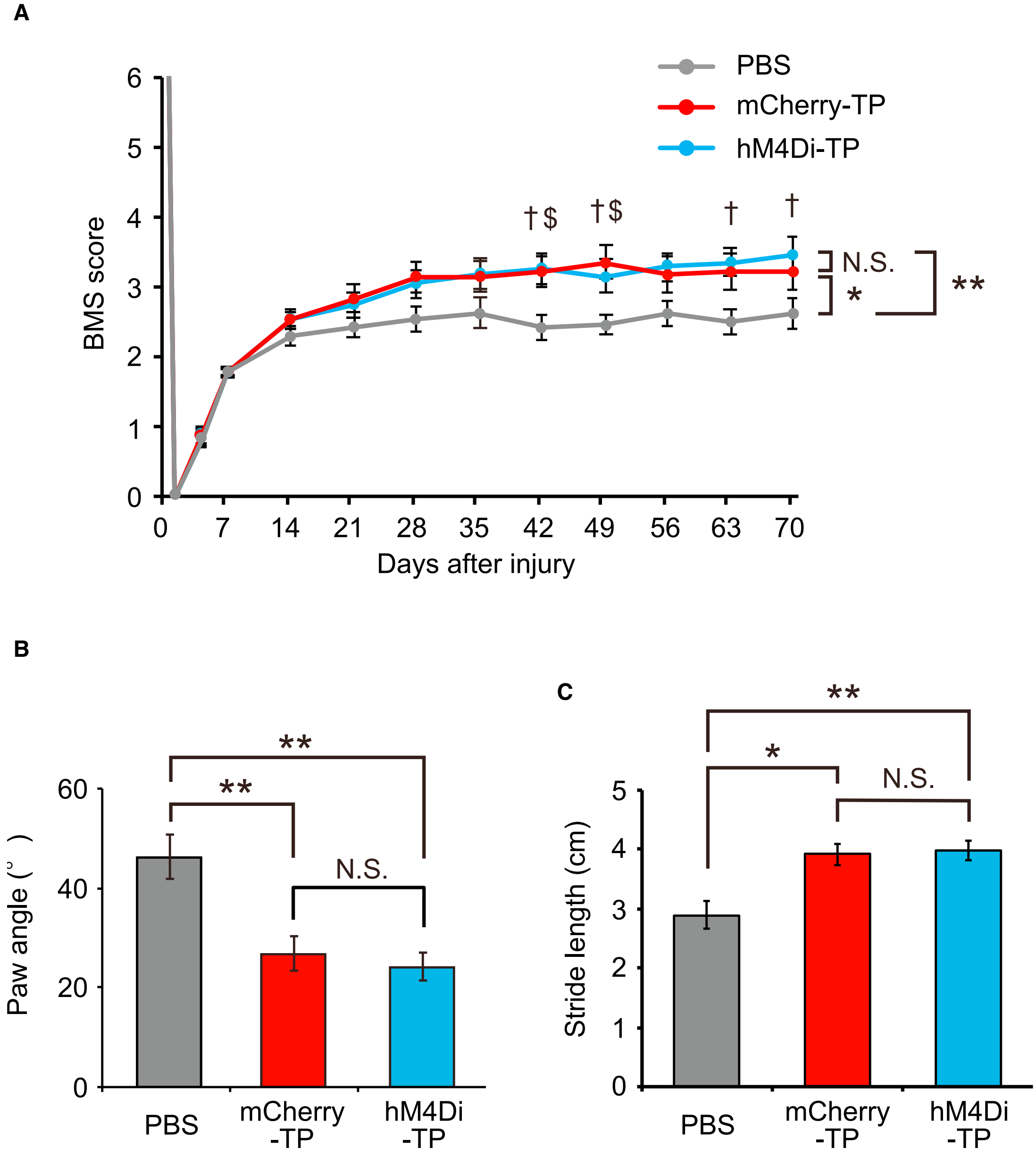

Figure 4. Transplanted NS/PCs promote functional recovery after SCI

(A) BMS scores before and after SCI in non-transplanted animals (PBS) and transplanted animals (mCherry-TP and hM4Di-TP) (PBS group, n = 20; mCherry-TP group, n = 18; hM4Di-TP group, n = 20).

(B) Comparison of paw angles determined by treadmill gait analysis performed at 9 weeks after transplantation (PBS group, n = 20; mCherry-TP group, n = 18; hM4Di-TP group, n = 20).

(C) Comparison of stride lengths determined by treadmill gait analysis performed at 9 weeks after transplantation (PBS group, n = 20; mCherry-TP group, n = 18; hM4Di-TP group, n = 20). $^{*}p < 0.05$, $^{**}p < 0.01$, and N.S., not significant according to the repeated-measures two-way ANOVA (A) and the Mann-Whitney U test following Kruskal-Wallis test (B and C). $^{\dagger}p < 0.05$, hM4Di-TP versus PBS, and $^{\$}p < 0.05$, mCherry-TP versus PBS, according to the two-sided unpaired Student t test. The data are presented as the mean ± SEM. All multiple testing data analyses were followed by Bonferroni correction.

washout, 3.2 ± 0.2, Friedman test, p = 0.143; hM4Di-TP: pre-CNO, 3.3 ± 0.2; post-CNO, 3.1 ± 0.2; washout, 3.3 ± 0.2, Freidman test, p = 0.010; *post hoc* pre-CNO versus post-CNO, adjusted p = 0.021; pre-CNO versus washout, adjusted p = 1.000; post-CNO versus washout, adjusted p = 0.174) (Figure 5C). Although the BMS scores of

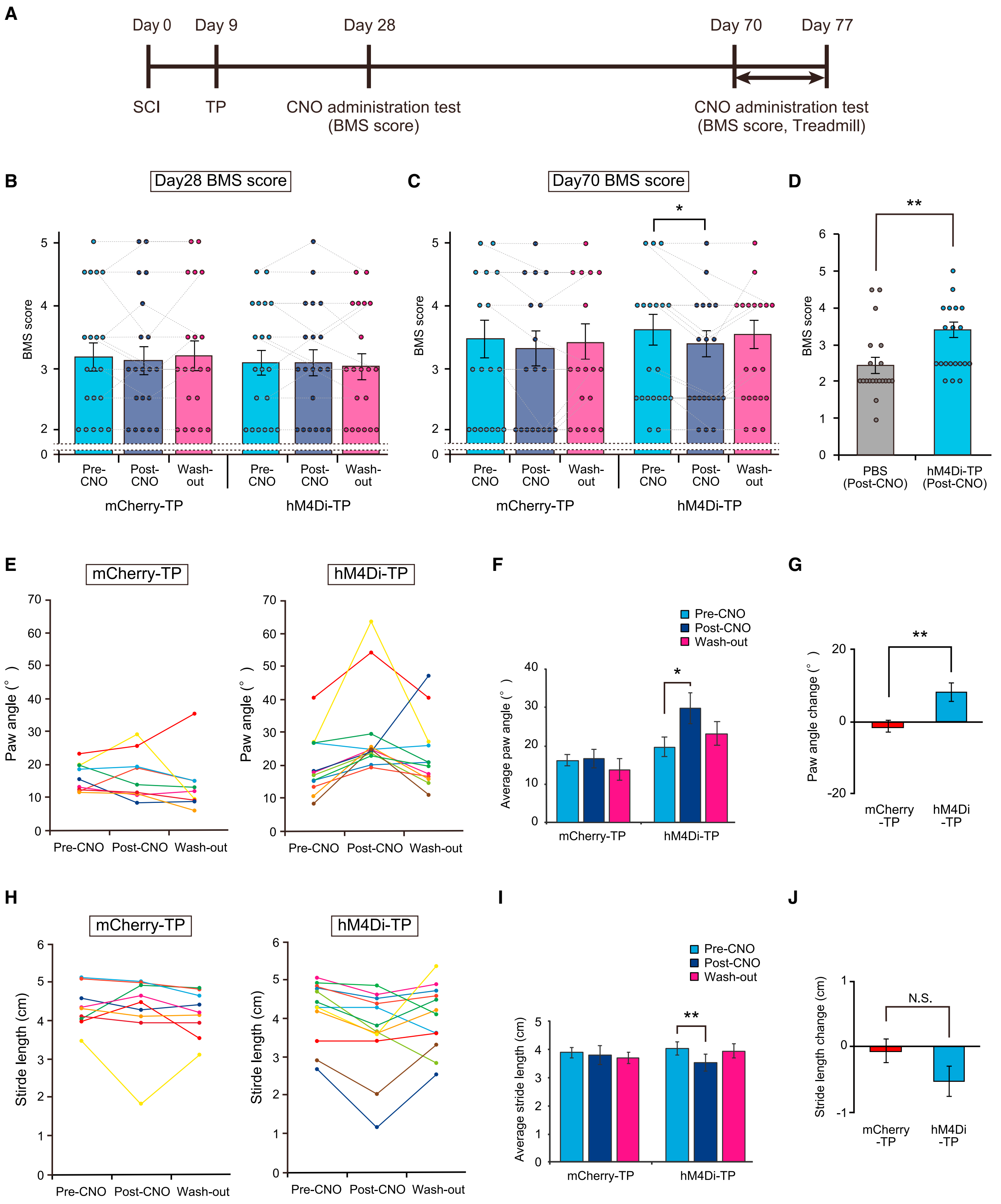

Figure 5. hM4Di-NS/PC-transplanted mice showed locomotor functional changes induced by the inhibition of graft neuronal activity

(A) Schematic representing the time schedule of the *in vivo* experiment.

(legend continued on next page)

hM4Di-TP mice declined following the inhibition of graft neuronal activity, the locomotor functions of mice in this condition remained refined compared with those of the non-transplanted animals, which showed no functional change after the administration of CNO (PBS post-CNO, 2.4 ± 0.2, hM4Di-TP versus PBS, p = 0.009) (Figures 5D, S4A, and S4B).

In the treadmill gait analysis test, the paw angle of the hM4Di-TP group was significantly enlarged following the administration of CNO (pre-CNO, 19.8° ± 2.6°; post-CNO, 29.9° ± 4.1°; washout, 23.2° ± 3.1°, Friedman test, p = 0.013; *post hoc* pre-CNO versus post-CNO, adjusted p = 0.009; pre-CNO versus washout, adjusted p = 0.717; post-CNO versus washout, adjusted p = 0.150) (Figures 5E and 5F). In addition, the stride length was significantly reduced after CNO administration in the hM4Di-TP group (pre-CNO, 4.2 ± 0.2 cm; post-CNO, 3.7 ± 0.3 cm; washout, 4.0 ± 0.2 cm, Friedman test, p = 0.013; *post hoc* pre-CNO versus post-CNO, adjusted p = 0.009; pre-CNO versus washout, adjusted p = 1.000; post-CNO versus washout, adjusted p = 0.618) (Figures 5H and 5I). In contrast, the parameters of treadmill gait analysis in the mCherry-TP group and the PBS group did not differ before and after CNO administration (mCherry-TP, paw angle: pre-CNO, 16.2° ± 1.4°; post-CNO, 16.6° ± 2.5°; washout, 13.8° ± 2.9°, Friedman test, p = 0.236; stride length: pre-CNO, 4.4 ± 0.2 cm; post-CNO, 4.3 ± 0.3 cm; washout, 4.2 ± 0.2 cm, Friedman test, p = 0.097) (Figures 5E, 5F, 5H, 5I, and S4C–S4F). The effect of CNO in the hM4Di-TP group tended to return to the basal levels after CNO withdrawal. In contrast, CNO administration did not alter the paw angles and stride lengths of mice in the mCherry-TP group. Assessment of functional changes before and after CNO administration revealed a significantly larger functional decline in the paw angle in the hM4Di-TP group compared with the mCherry-TP group and a tendency for a more substantial change in stride length in the hM4Di-TP group (paw angle: 10.2° ± 2.8° versus 0.4° ± 1.8°, p = 0.009; stride length: −0.5 ± 0.1 cm versus −0.1 ± 0.2, p = 0.069) (Figures 5G and 5J).

Together, the results show that locomotor function of only the hM4Di-TP group was controlled by CNO administration, suggesting that inhibiting the neuronal activity of the graft neurons adversely affected the locomotor functions of the host mice.

The functional change induced by graft neuronal activity inhibition was sustained in the mice during long-term follow-up

To confirm the sustained therapeutic effect of graft neuronal activity, we administered CNO to mice followed for 17 weeks after transplantation. Similar to the experiment at 9 weeks after transplantation, the BMS score significantly decreased in the hM4Di-TP group after administration of CNO (mCherry-TP: pre-CNO, 3.6 ± 0.4; post-CNO, 3.5 ± 0.5; washout, 3.5 ± 0.4; Friedman test, p = 0.607; hM4Di-TP: pre-CNO, 3.1 ± 0.5; post-CNO, 2.6 ± 0.5; washout, 3.1 ± 0.5, Friedman test, p < 0.001; *post hoc* pre-CNO versus post-CNO, adjusted p = 0.027; pre-CNO versus washout, adjusted p = 1.000; post-CNO versus washout, adjusted p = 0.027) (Figure 6A). The parameters of the treadmill gait analysis in hM4Di-TP animals showed a significantly larger change in paw angle and tended to show a decrease in stride length (Figures S5A–S5F).

Electrophysiological analysis by neuronal activity inhibition was also performed. The motor evoked potential (MEP) wave was analyzed before and after CNO administration, and the post-CNO parameters were normalized based on the pre-CNO parameters. The normalized maximal amplitude was significantly lower and the normalized peak latency was significantly longer in the hM4Di-TP

(B and C) BMS scores before CNO administration, after CNO administration, and after CNO washout. The scores at 28 days after SCI (B) and 70 days after SCI (C) are presented (mCherry-TP group, n = 18; hM4Di-TP group, n = 20).
(D) Comparison of BMS scores in CNO-administered mice on day 70 (PBS group, n = 20; hM4Di-TP group, n = 20).
(E) Raw data of paw angles before CNO administration, after CNO administration, and after CNO washout for mCherry-TP mice (left) and hM4Di-TP mice (right).
(F) Quantification of paw angles before CNO administration, after CNO administration, and after CNO washout (mCherry-TP group, n = 9; hM4Di-TP group, n = 12).
(G) Quantification of the paw angle changes before and after CNO administration in each animal (mCherry-TP group, n = 9; hM4Di-TP group, n = 12).
(H) Raw stride-length data before CNO administration, after CNO administration, and after CNO washout for mCherry-TP mice (left) and hM4Di-TP mice (right).
(I) Quantification of stride length before CNO administration, after CNO administration, and after CNO washout (mCherry-TP group, n = 9; hM4Di-TP group, n = 12).
(J) Quantification of the stride length changes before and after CNO administration in each animal (mCherry-TP group, n = 9; hM4Di-TP group, n = 12). *p < 0.05, **p < 0.01, and N.S., not significant (not displayed in the figures [B, C, F, and I]), according to the Wilcoxon signed rank test following Friedmann test (B, C, F, and I) and the Mann-Whitney U test (D, G, and J). The data are presented as the mean ± SEM (B–D, F, G, I, and J). All multiple testing data analyses were followed by Bonferroni correction.

group compared with the mCherry-TP group (normalized maximal amplitude 1.0 ± 0.1 versus 0.6 ± 0.1, p = 0.026; normalized peak latency 1.0 ± 0.04 versus 1.1 ± 0.03, p = 0.015) (Figures 6B and 6C). Accordingly, the functional decline of host animals by inhibiting engrafted neuronal activity was sustained in long-term-followed animals.

Trans-synaptic tracing revealed the integration of engrafted neurons into the host motor circuit

As presented above, differentiated neuronal cells directly affected locomotor function. To clarify the integration of graft neurons into the host motor circuitry, trans-synaptic tracing by wheat germ agglutinin (WGA) was performed (Fabian and Coulter, 1985). A Tet-inducible lentiviral vector containing the AcGFP1 and WGA genes linked by the porcine teschovirus-1 2A peptide sequence was transfected into NS/PCs (WGA-NS/PCs) (Figure 7A) (Ohashi et al., 2011). NS/PCs were transplanted into mice with thoracic contusion injuries in the subacute phase, and the mice were fed a rodent diet containing doxycycline for 2 weeks before their spinal cord sections were extracted (Kojima et al., 2019).

IHC analyses were performed to confirm the presence of WGA^{+}/AcGFP1^{-} cells, indicating trans-synaptic migration of WGA and thereby suggesting the synaptic connection of transplanted neurons (Figure 7B). Migration of WGA to ELAVL3/4^{+} neurons was confirmed, indicating the synaptic connection of graft neurons with other neurons (Figure 7C). WGA showed migration to both rostral and caudal sites. The WGA-positive area decreased with distance from the transplanted site; however, WGA was still observed 4 mm caudal to the epicenter (Figures S6A and S6B). As for migrated cells, WGA was detected not only in HNA^{+}/AcGFP1^{-} graft cells but also in HNA^{-} host cells, demonstrating the synaptic connection between graft neurons and host neurons (Figure 7D). To validate the connection with host motor neurons, we evaluated the migration of WGA to choline acetyltransferase (ChAT)$^{+}$ motor neurons at the caudal site (Figure 7E). WGA was confirmed in the ChAT^{+} host motor neurons in the region of lumbar enlargement, demonstrating that the engrafted neurons were integrated into the host motor circuits.

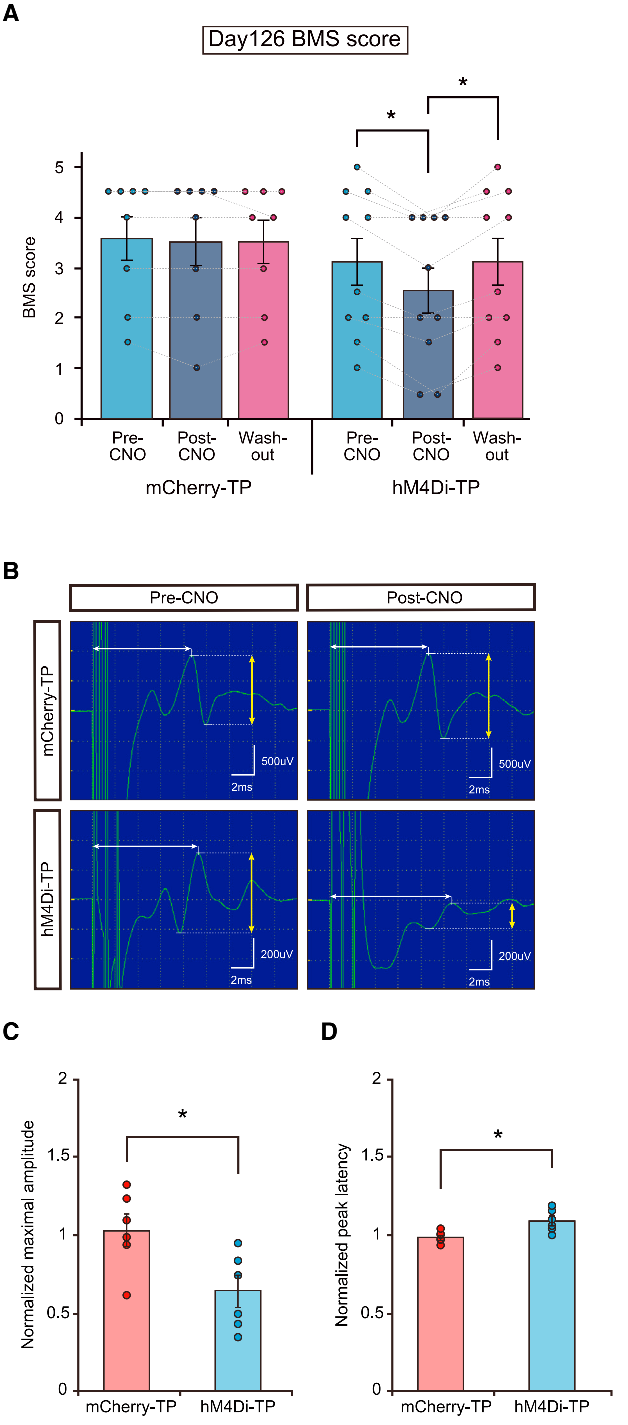

Figure 6. Neuronal activity inhibition of engrafted cells affected the locomotor function and electrophysiological parameters in long-term-followed animals

(A) BMS scores before CNO administration, after CNO administration, and after CNO washout (mCherry-TP group, n = 8; hM4Di-TP group, n = 10).

(B) Representative images of MEP waves in mCherry-TP animals and hM4Di-TP animals (left, pre-CNO; right, post-CNO).

(C) Average of normalized maximal amplitude in mCherry-TP group and hM4Di-TP group (mCherry-TP group, n = 6; hM4Di-TP group, n = 6).

(D) Average of normalized peak latency in mCherry-TP group and hM4Di-TP group (mCherry-TP group, n = 6; hM4Di-TP group, n = 6).

*$p < 0.05$ according to the Wilcoxon signed rank test following Friedman test (A) and the Mann-Whitney U test (C and D). The data are presented as the mean ± SEM (A, C, and D). All multiple testing data analyses were followed by Bonferroni correction.

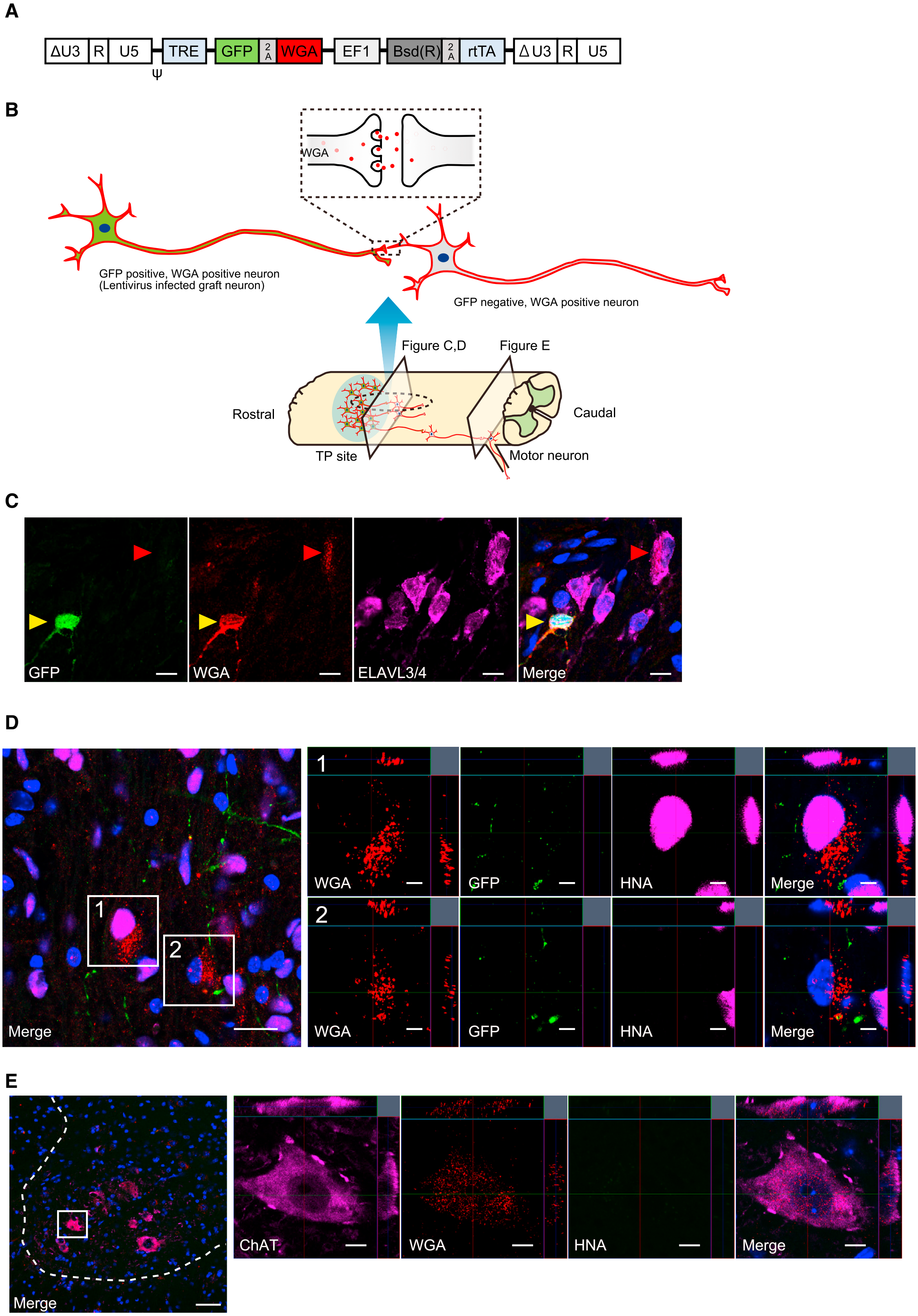

(legend on next page)

DISCUSSION

By generating hM4Di-expressing hiPSC-NS/PCs, we have shown the chemical inhibition of neuronal activity via a reduction in intracellular calcium events and attenuation of electrical activity. The precise and remote inhibition of neuronal activity was confirmed *in vitro*, and when these cells were transplanted during the subacute phase of SCI, recovered locomotor function at the chronic stage transiently declined following the administration of CNO in host animals. This neuronal function was corroborated by the trans-synaptic tracing of WGA, revealing the integration of grafted neurons into the host motor circuits. These results directly demonstrated the significance of neuronal activity in grafted hiPSC-NS/PCs for behavioral improvement after SCI.

Intriguingly, loss of motor function induced by the DREADD was not observed at 3 weeks but was observed after 9 weeks after transplantation, indicating that the grafted neurons remained immature at the early stage after transplantation and reached maturity over time, thereby leading to locomotor function. In parallel to these findings, our previous histological analyses revealed that $NeuN^{+}$ mature neuronal cell numbers increased gradually from 6 to 14 weeks after hiPSC-NS/PC transplantation (Nori et al., 2011). These results were corroborated by other *in vitro* studies revealing that cultured hiPSC-derived neurons required approximately 6 weeks to form synaptic connections and evoke spontaneous neuronal activity (Meijer et al., 2019; Wilson and Newell-Litwa, 2018). Together, these findings suggest that a certain amount of time is required for the transplanted NS/PCs to differentiate and mature into functional neurons, which contributes to locomotor improvement.

On the other hand, the DREADD-mediated inhibition of graft neuronal activity only partially deteriorated locomotor function, which did not reach the level of that in non-transplanted SCI animals. This suggests that graft-derived beneficial factors other than neuronal function influenced locomotor recovery. A candidate factor underlying this result is neuroprotection by the trophic factors. We previously demonstrated the secretion of nerve growth factor, brain-derived neurotrophic factor, and hepatocyte growth factor from hiPSC-derived NS/PCs, and these factors exerted a favorable effect on behavior soon after transplantation (Nori et al., 2011). The slight improvement in the early phase after transplantation could be theorized as the effect of these factors. An alternative factor for the beneficial mechanisms was remyelination by the transplanted NS/PC-derived oligodendrocytes (Assinck et al., 2017; Kawabata et al., 2016; Nori et al., 2017). However, the therapeutic effect of remyelination is still controversial (Duncan et al., 2018; Yasuda et al., 2011). The present study revealed that the frequency of oligodendrocyte differentiation was approximately 10%, which was noticeably lower than the more than 50% rate for neuronal differentiation. Although a small population of differentiated myelinating cells might contribute to locomotion, further detailed studies are required to estimate the contribution of differentiated oligodendrocytes. Taken together, hiPSC-NS/PCs most likely promote functional recovery via multiple mechanisms, and the present study demonstrated at least the significance of differentiated neuronal cells that directly contribute to locomotion.

The safety of using hiPSCs as a cell source for NS/PCs must be verified due to tumorigenicity concerns (Iida et al., 2017; Tanimoto et al., 2020). We have previously addressed this issue by using a Notch signal inhibitor (γ-secretase inhibitor) to suppress cell proliferation and promote neuronal differentiation (Okubo et al., 2016, 2018). In the present study, we prevented tumor-like overgrowth by pretreating NS/PCs with this inhibitor and successfully engrafted neuronal cells constituting functional neuronal circuits. Since we plan to use this inhibitor in actual clinical trials (Tsuji et al., 2019), our strategy of using hiPSCs for SCI could be validated in terms of both efficacy and safety.

The results of the current study raise the question of which neuronal cells derived from the graft most influence the locomotor function of host animals. Although there are several reports indicating functional recovery by maintaining the inhibitory and excitatory balance of neurons following SCI (Chen et al., 2018; Fandel et al., 2016; Huang et al., 2016), our present study did not reveal which

Figure 7. WGA expressed in graft neurons demonstrates the trans-synaptic integration into host neuronal circuits

(A) Schematic illustration of the integrated proviral form of the Tet-inducible lentiviral vector CSIV-TRE-AcGFP1-WGA-EF1a-Bsd(R)-rtTA2M2.

(B) Schematic image of the experiments. TP site, transplantation site.

(C) Representative images of migrating WGA. The yellow arrowheads indicate lentivirus-infected graft neurons (GFP, WGA double-positive neurons) and the red arrowheads indicate migrated WGA in GFP-negative neurons. Scale bars, 10 μm.

(D) GFP, WGA, and HNA staining identifies cells connected to lentivirus-infected neurons. Enlarged image 1 shows a non-lentivirus-infected graft cell stained with WGA. Enlarged image 2 shows a host cell stained with WGA. Scale bars, 20 and 5 μm (enlarged images).

(E) HNA, WGA, and ChAT staining revealed host motor neurons connected to lentivirus-infected neurons. Scale bars, 50 and 10 μm (enlarged images). Dashed whitline showes the edge of gray matter.

subtype of neuronal cells derived from the graft contributed to functional recovery. In addition, the connectivity of grafted neurons to each spinal tract is a matter of great concern and could also be important for clarifying the therapeutic mechanisms (Adler et al., 2017; Kumamaru et al., 2018, 2019). Further investigation will uncover more detailed functional contributions of NS/PC transplantation to the treatment of SCI.

In conclusion, we have successfully controlled the activity of neurons derived from hiPSC-NS/PCs in the injured spinal cord and confirmed the change in locomotor function induced by the suppression of graft-derived neuronal activity. The present study provided some mechanistic insight into functional recovery from SCI after hiPSC-NS/PC transplantation.

EXPERIMENTAL PROCEDURES

Lentiviral vector preparation

Recombinant lentiviral vector production was performed as described previously (Miyoshi et al., 1998). The details are described in the supplemental information.

Cell culture, lentiviral transduction, and *in vitro* neuronal differentiation assay

NS/PCs were cultured from a non-tumorigenic hiPSC clone (414C2), and lentiviral transduction was performed as previously reported (Nori et al., 2011; Okada et al., 2008; Okubo et al., 2018).

Differentiated NS/PCs were cultured for 30 days and ICC stained to evaluate the differentiation potential. Cultured cells were also evaluated by calcium imaging and MEA analyses. The detailed methods are described in the supplemental information.

SCI animal model and NS/PC transplantation

Contusive SCI was induced at the level of the 10th thoracic spinal vertebra in immunodeficient mice. Transplantation of NS/PCs was performed at 9 days after injury as previously reported (Kamata et al., 2021; Okubo et al., 2016). The details are provided in the supplemental information.

Histological analyses

Histological analyses were performed by H&E staining and IHC staining. The detailed methods and antibodies used for IHC staining are described in the supplemental information.

Immunoelectron microscopy analysis

Immunoelectron microscopy analysis was performed on spinal cord sections from hM4Di-TP mice and mCherry-TP mice. The detailed procedure was described previously (Shibata et al., 2019) and is provided in the supplemental information.

Neural activity inhibition assay (*in vivo*)

BMS scores (Basso et al., 2006) and treadmill gait analysis were evaluated before, 1 h after, and 24 h after CNO (10 mg/kg) administration. The detailed methods are described in the supplemental information.

Electrophysiology

Electrophysiological experiments were performed in long-term-followed hM4Di-TP mice and mCherry-TP mice. The detailed methods are described in the supplemental information.

Statistical analyses

Statistical analyses were performed using SPSS statistics (Japan IBM, Tokyo, Japan, v.26.0.0.0). Data are reported as the mean ± SEM. The sample sizes are indicated in the respective figure legends. The Mann-Whitney U test was performed for the *in vitro* ICC staining, calcium imaging, MEA imaging, *in vivo* IHC staining, MEP analysis, BMS score, and treadmill gait analyses in a non-paired situation. The Wilcoxon signed rank test was used to compare the BMS score and the treadmill parameter changes in the neuronal activity inhibition assay. Repeated-measures two-way ANOVA was used for the weekly BMS scoring followed by two-sided unpaired Student's t test for each time point. All multiple testing data were analyzed by the Kruskal-Wallis test (non-paired) or Friedman test (paired) and *post hoc* analysis with Bonferroni correction (described as adjusted p). *p* values of <0.05 indicate statistical significance (*$p < 0.05$ and **$p < 0.01$).

SUPPLEMENTAL INFORMATION

Supplemental information can be found online at https://doi.org/10.1016/j.stemcr.2021.12.005.

AUTHOR CONTRIBUTIONS

T.K. designed the project, performed most of the experiments, and interpreted the data. T.K. and N.N. wrote the manuscript. N.N., Y.K., M.K., K.A., K.K., R.S., Y.S., K.I., M.S., J.K., T.S., and S.S. provided experimental support and ideas for the project. M.N. and H.O. supervised the overall project. T.K., N.N., H.O., and M.N. provided financial support. N.N., M.M., M.N., and H.O. edited the manuscript and gave administrative support. All authors read and approved the final manuscript.

CONFLICT OF INTERESTS

H.O. and M.N. are compensated scientific consultants to K Pharma, and H.O. is also a compensated scientific consultant to SanBio Ltd. No management, preparation, analysis, interpretation, or review of data was performed by the funding sources.

ACKNOWLEDGMENTS

We appreciate the assistance and instruction provided by Drs. M. Ishikawa, H. Miyoshi, S. Nori, O. Tsuji, K. Kojima, S. Ito, Y. Tanimoto, Y. Hoshino, S. Hashimoto, and T. Shibata, all of whom are members of the spinal cord research team at the Department of Orthopedic Surgery and Physiology, Keio University School of Medicine, Tokyo, Japan. We also appreciate the advice from Dr. K. Tanaka (Department of Psychiatry, Keio University School of Medicine, Tokyo, Japan). We thank Prof. S. Yamanaka (Kyoto University, Kyoto, Japan) for the human iPSC

clone and Dr. Y. Miyashita (The University of Tokyo, Tokyo, Japan) for providing a vector containing WGA. We also thank T. Harada, K. Yasutake, M. Akizawa, K. Matsumura, and R. Ikeda for their assistance with the experiments and animal care. This work was supported by the following grants: Research Center Network for Realization of Regenerative Medicine by AMED Japan under grant nos. JP21bm0204001, JP20bm0204001, JP19bm0204001, JP20bk0104017, and JP19bk0104017 to H.O. and M.N., and JP20bm0704046 to S.S. and T.S.; Keio University Grant-in-Aid for Encouragement of Young Medical Scientists (2019 and 2020) to T.K.; and The General Insurance Association of Japan Medical Research Grants 2019 and 2020 to T.K.

Received: May 25, 2021
Revised: December 10, 2021
Accepted: December 11, 2021
Published: January 11, 2022

REFERENCES

Abematsu, M., Tsujimura, K., Yamano, M., Saito, M., Kohno, K., Kohyama, J., Namihira, M., Komiya, S., and Nakashima, K. (2010). Neurons derived from transplanted neural stem cells restore disrupted neuronal circuitry in a mouse model of spinal cord injury. J. Clin. Invest. *120*, 3255–3266. https://doi.org/10.1172/JCI42957.

Adler, A.F., Lee-Kubli, C., Kumamaru, H., Kadoya, K., and Tuszynski, M.H. (2017). Comprehensive monosynaptic rabies virus mapping of host connectivity with neural progenitor grafts after spinal cord injury. Stem Cell Rep. *8*, 1525–1533. https://doi.org/10.1016/j.stemcr.2017.04.004.

Aldrin-Kirk, P., Heuer, A., Wang, G., Mattsson, B., Lundblad, M., Parmar, M., and Bjorklund, T. (2016). DREADD modulation of transplanted DA neurons reveals a novel parkinsonian dyskinesia mechanism mediated by the serotonin 5-HT6 receptor. Neuron *90*, 955–968. https://doi.org/10.1016/j.neuron.2016.04.017.

Assinck, P., Duncan, G.J., Hilton, B.J., Plemel, J.R., and Tetzlaff, W. (2017). Cell transplantation therapy for spinal cord injury. Nat. Neurosci. *20*, 637–647. https://doi.org/10.1038/nn.4541.

Basso, D.M., Fisher, L.C., Anderson, A.J., Jakeman, L.B., McTigue, D.M., and Popovich, P.G. (2006). Basso mouse scale for locomotion detects differences in recovery after spinal cord injury in five common mouse strains. J. Neurotrauma *23*, 635–659. https://doi.org/10.1089/neu.2006.23.635.

Bonner, J.F., Connors, T.M., Silverman, W.F., Kowalski, D.P., Lemay, M.A., and Fischer, I. (2011). Grafted neural progenitors integrate and restore synaptic connectivity across the injured spinal cord. J. Neurosci. *31*, 4675–4686. https://doi.org/10.1523/JNEUROSCI.4130-10.2011.

Bonner, J.F., and Steward, O. (2015). Repair of spinal cord injury with neuronal relays: from fetal grafts to neural stem cells. Brain Res. *1619*, 115–123. https://doi.org/10.1016/j.brainres.2015.01.006.

Ceto, S., Sekiguchi, K.J., Takashima, Y., Nimmerjahn, A., and Tuszynski, M.H. (2020). Neural stem cell grafts form extensive synaptic networks that integrate with host circuits after spinal cord injury. Cell Stem Cell *27*, 430–440.e435. https://doi.org/10.1016/j.stem.2020.07.007.

Chen, B., Li, Y., Yu, B., Zhang, Z., Brommer, B., Williams, P.R., Liu, Y., Hegarty, S.V., Zhou, S., Zhu, J., et al. (2018). Reactivation of dormant relay pathways in injured spinal cord by KCC2 manipulations. Cell *174*, 1599. https://doi.org/10.1016/j.cell.2018.08.050.

Chen, Y., Xiong, M., Dong, Y., Haberman, A., Cao, J., Liu, H., Zhou, W., and Zhang, S.C. (2016). Chemical control of grafted human PSC-derived neurons in a mouse model of Parkinson's disease. Cell Stem Cell *18*, 817–826. https://doi.org/10.1016/j.stem.2016.03.014.

Cummings, B.J., Uchida, N., Tamaki, S.J., Salazar, D.L., Hooshmand, M., Summers, R., Gage, F.H., and Anderson, A.J. (2005). Human neural stem cells differentiate and promote locomotor recovery in spinal cord-injured mice. Proc. Natl. Acad. Sci. U S A *102*, 14069–14074. https://doi.org/10.1073/pnas.0507063102.

Dana, H., Sun, Y., Mohar, B., Hulse, B.K., Kerlin, A.M., Hasseman, J.P., Tsegaye, G., Tsang, A., Wong, A., Patel, R., et al. (2019). High-performance calcium sensors for imaging activity in neuronal populations and microcompartments. Nat. Methods *16*, 649–657. https://doi.org/10.1038/s41592-019-0435-6.

Dell'Anno, M.T., Wang, X., Onorati, M., Li, M., Talpo, F., Sekine, Y., Ma, S., Liu, F., Cafferty, W.B.J., Sestan, N., and Strittmatter, S.M. (2018). Human neuroepithelial stem cell regional specificity enables spinal cord repair through a relay circuit. Nat. Commun. *9*, 3419. https://doi.org/10.1038/s41467-018-05844-8.

Duncan, G.J., Manesh, S.B., Hilton, B.J., Assinck, P., Liu, J., Moulson, A., Plemel, J.R., and Tetzlaff, W. (2018). Locomotor recovery following contusive spinal cord injury does not require oligodendrocyte remyelination. Nat. Commun. *9*, 3066. https://doi.org/10.1038/s41467-018-05473-1.

Egashira, Y., Mori, Y., Yanagawa, Y., and Takamori, S. (2018). Development of lentiviral vectors for efficient glutamatergic-selective gene expression in cultured hippocampal neurons. Sci. Rep. *8*, 15156. https://doi.org/10.1038/s41598-018-33509-5.

Fabian, R.H., and Coulter, J.D. (1985). Transneuronal transport of lectins. Brain Res. *344*, 41–48. https://doi.org/10.1016/0006-8993(85)91187-4.

Fandel, T.M., Trivedi, A., Nicholas, C.R., Zhang, H., Chen, J., Martinez, A.F., Noble-Haeusslein, L.J., and Kriegstein, A.R. (2016). Transplanted human stem cell-derived interneuron precursors mitigate mouse bladder dysfunction and central neuropathic pain after spinal cord injury. Cell Stem Cell *19*, 544–557. https://doi.org/10.1016/j.stem.2016.08.020.

Fujimoto, Y., Abematsu, M., Falk, A., Tsujimura, K., Sanosaka, T., Juliandi, B., Semi, K., Namihira, M., Komiya, S., Smith, A., and Nakashima, K. (2012). Treatment of a mouse model of spinal cord injury by transplantation of human induced pluripotent stem cell-derived long-term self-renewing neuroepithelial-like stem cells. Stem Cells *30*, 1163–1173. https://doi.org/10.1002/stem.1083.

Gray, E.G. (1959). Electron microscopy of synaptic contacts on dendrite spines of the cerebral cortex. Nature *183*, 1592–1593. https://doi.org/10.1038/1831592a0.

Huang, Y.J., Lee, K.H., Murphy, L., Garraway, S.M., and Grau, J.W. (2016). Acute spinal cord injury (SCI) transforms how GABA affects nociceptive sensitization. Exp. Neurol. *285*, 82–95. https://doi.org/10.1016/j.expneurol.2016.09.005.

Iida, T., Iwanami, A., Sanosaka, T., Kohyama, J., Miyoshi, H., Nagoshi, N., Kashiwagi, R., Toyama, Y., Matsumoto, M., Nakamura, M., and Okano, H. (2017). Whole-genome DNA methylation analyses revealed epigenetic instability in tumorigenic human ips cell-derived neural stem/progenitor cells. Stem Cells *35*, 1316–1327. https://doi.org/10.1002/stem.2581.

Kadoya, K., Lu, P., Nguyen, K., Lee-Kubli, C., Kumamaru, H., Yao, L., Knackert, J., Poplawski, G., Dulin, J.N., Strobl, H., et al. (2016). Spinal cord reconstitution with homologous neural grafts enables robust corticospinal regeneration. Nat. Med. *22*, 479–487. https://doi.org/10.1038/nm.4066.

Kamata, Y., Isoda, M., Sanosaka, T., Shibata, R., Ito, S., Okubo, T., Shinozaki, M., Inoue, M., Koya, I., Shibata, S., et al. (2021). A robust culture system to generate neural progenitors with gliogenic competence from clinically relevant induced pluripotent stem cells for treatment of spinal cord injury. Stem Cells Transl. Med. *10*, 398–413. https://doi.org/10.1002/sctm.20-0269.

Kawabata, S., Takano, M., Numasawa-Kuroiwa, Y., Itakura, G., Kobayashi, Y., Nishiyama, Y., Sugai, K., Nishimura, S., Iwai, H., Isoda, M., et al. (2016). Grafted human iPS Cell-Derived oligodendrocyte precursor cells contribute to robust remyelination of demyelinated axons after spinal cord injury. Stem Cell Rep. *6*, 1–8. https://doi.org/10.1016/j.stemcr.2015.11.013.

Kobayashi, Y., Okada, Y., Itakura, G., Iwai, H., Nishimura, S., Yasuda, A., Nori, S., Hikishima, K., Konomi, T., Fujiyoshi, K., et al. (2012). Pre-evaluated safe human iPSC-derived neural stem cells promote functional recovery after spinal cord injury in common marmoset without tumorigenicity. PLoS One *7*, e52787. https://doi.org/10.1371/journal.pone.0052787.

Kojima, K., Miyoshi, H., Nagoshi, N., Kohyama, J., Itakura, G., Kawabata, S., Ozaki, M., Iida, T., Sugai, K., Ito, S., et al. (2019). Selective ablation of tumorigenic cells following human induced pluripotent stem cell-derived neural stem/progenitor cell transplantation in spinal cord injury. Stem Cells Transl. Med *8*, 260–270. https://doi.org/10.1002/sctm.18-0096.

Kumamaru, H., Kadoya, K., Adler, A.F., Takashima, Y., Graham, L., Coppola, G., and Tuszynski, M.H. (2018). Generation and post-injury integration of human spinal cord neural stem cells. Nat. Methods *15*, 723–731. https://doi.org/10.1038/s41592-018-0074-3.

Kumamaru, H., Lu, P., Rosenzweig, E.S., Kadoya, K., and Tuszynski, M.H. (2019). Regenerating corticospinal axons innervate phenotypically appropriate neurons within neural stem cell grafts. Cell Rep. *26*, 2329–2339.e2324. https://doi.org/10.1016/j.celrep.2019.01.099.

Lu, P., Gomes-Leal, W., Anil, S., Dobkins, G., Huie, J.R., Ferguson, A.R., Graham, L., and Tuszynski, M. (2019). Origins of neural progenitor cell-derived axons projecting caudally after spinal cord injury. Stem Cell Rep. *13*, 105–114. https://doi.org/10.1016/j.stemcr.2019.05.011.

Lu, P., Wang, Y., Graham, L., McHale, K., Gao, M., Wu, D., Brock, J., Blesch, A., Rosenzweig, E.S., Havton, L.A., et al. (2012). Long-distance growth and connectivity of neural stem cells after severe spinal cord injury. Cell *150*, 1264–1273. https://doi.org/10.1016/j.cell.2012.08.020.

Meijer, M., Rehbach, K., Brunner, J.W., Classen, J.A., Lammertse, H.C.A., van Linge, L.A., Schut, D., Krutenko, T., Hebisch, M., Cornelisse, L.N., et al. (2019). A single-cell model for synaptic transmission and plasticity in human ipsc-derived neurons. Cell Rep. *27*, 2199–2211.e2196. https://doi.org/10.1016/j.celrep.2019.04.058.

Miyoshi, H., Blomer, U., Takahashi, M., Gage, F.H., and Verma, I.M. (1998). Development of a self-inactivating lentivirus vector. J. Virol. *72*, 8150–8157. https://doi.org/10.1128/JVI.72.10.8150-8157.1998.

Nagoshi, N., Okano, H., and Nakamura, M. (2020). Regenerative therapy for spinal cord injury using iPSC technology. Inflamm. Regen. *40*, 40. https://doi.org/10.1186/s41232-020-00149-0.

Nichols, C.D., and Roth, B.L. (2009). Engineered G-protein coupled receptors are powerful tools to investigate biological processes and behaviors. Front Mol. Neurosci. *2*, 16. https://doi.org/10.3389/neuro.02.016.2009.

Nori, S., Nakamura, M., and Okano, H. (2017). Plasticity and regeneration in the injured spinal cord after cell transplantation therapy. Prog. Brain Res. *231*, 33–56. https://doi.org/10.1016/bs.pbr.2016.12.007.

Nori, S., Okada, Y., Yasuda, A., Tsuji, O., Takahashi, Y., Kobayashi, Y., Fujiyoshi, K., Koike, M., Uchiyama, Y., Ikeda, E., et al. (2011). Grafted human-induced pluripotent stem-cell-derived neurospheres promote motor functional recovery after spinal cord injury in mice. Proc. Natl. Acad. Sci. U S A *108*, 16825–16830. https://doi.org/10.1073/pnas.1108077108.

Ohashi, Y., Tsubota, T., Sato, A., Koyano, K.W., Tamura, K., and Miyashita, Y. (2011). A bicistronic lentiviral vector-based method for differential transsynaptic tracing of neural circuits. Mol. Cell. Neurosci. *46*, 136–147. https://doi.org/10.1016/j.mcn.2010.08.013.

Okada, Y., Matsumoto, A., Shimazaki, T., Enoki, R., Koizumi, A., Ishii, S., Itoyama, Y., Sobue, G., and Okano, H. (2008). Spatiotemporal recapitulation of central nervous system development by murine embryonic stem cell-derived neural stem/progenitor cells. Stem Cells *26*, 3086–3098. https://doi.org/10.1634/stemcells.2008-0293.

Okubo, T., Iwanami, A., Kohyama, J., Itakura, G., Kawabata, S., Nishiyama, Y., Sugai, K., Ozaki, M., Iida, T., Matsubayashi, K., et al. (2016). Pretreatment with a gamma-secretase inhibitor prevents tumor-like overgrowth in human ipsc-derived transplants for spinal cord injury. Stem Cell Rep. *7*, 649–663. https://doi.org/10.1016/j.stemcr.2016.08.015.

Okubo, T., Nagoshi, N., Kohyama, J., Tsuji, O., Shinozaki, M., Shibata, S., Kase, Y., Matsumoto, M., Nakamura, M., and Okano, H. (2018). Treatment with a gamma-secretase inhibitor promotes functional recovery in human ipsc- derived transplants for chronic spinal cord injury. Stem Cell Rep. *11*, 1416–1432. https://doi.org/10.1016/j.stemcr.2018.10.022.

Roth, B.L. (2016). DREADDs for neuroscientists. Neuron *89*, 683–694. https://doi.org/10.1016/j.neuron.2016.01.040.

Sato, T., Imaizumi, K., Watanabe, H., Ishikawa, M., and Okano, H. (2021). Generation of region-specific and high-purity neurons from human feeder-free iPSCs. Neurosci. Lett. *746*, 135676. https://doi.org/10.1016/j.neulet.2021.135676.

Shibata, S., Iseda, T., Mitsuhashi, T., Oka, A., Shindo, T., Moritoki, N., Nagai, T., Otsubo, S., Inoue, T., Sasaki, E., et al. (2019). Large-area fluorescence and electron microscopic correlative imaging with multibeam scanning electron microscopy. Front Neural Circuits *13*, 29. https://doi.org/10.3389/fncir.2019.00029.

Tanimoto, Y., Yamasaki, T., Nagoshi, N., Nishiyama, Y., Nori, S., Nishimura, S., Iida, T., Ozaki, M., Tsuji, O., Ji, B., et al. (2020). In vivo monitoring of remnant undifferentiated neural cells following human induced pluripotent stem cell-derived neural stem/progenitor cells transplantation. Stem Cells Transl. Med *9*, 465–477. https://doi.org/10.1002/sctm.19-0150.

Tsuji, O., Sugai, K., Yamaguchi, R., Tashiro, S., Nagoshi, N., Kohyama, J., Iida, T., Ohkubo, T., Itakura, G., Isoda, M., et al. (2019). Concise review: laying the groundwork for a first-in-human study of an induced pluripotent stem cell-based intervention for spinal cord injury. Stem Cells *37*, 6–13. https://doi.org/10.1002/stem.2926.

Wilson, E.S., and Newell-Litwa, K. (2018). Stem cell models of human synapse development and degeneration. Mol. Biol. Cell *29*, 2913–2921. https://doi.org/10.1091/mbc.E18-04-0222.

Yasuda, A., Tsuji, O., Shibata, S., Nori, S., Takano, M., Kobayashi, Y., Takahashi, Y., Fujiyoshi, K., Hara, C.M., Miyawaki, A., et al. (2011). Significance of remyelination by neural stem/progenitor cells transplanted into the injured spinal cord. Stem Cells *29*, 1983–1994. https://doi.org/10.1002/stem.767.

Zou, D., Chen, L., Deng, D., Jiang, D., Dong, F., McSweeney, C., Zhou, Y., Liu, L., Chen, G., Wu, Y., and Mao, Y. (2016). DREADD in parvalbumin interneurons of the dentate gyrus modulates anxiety, social interaction and memory extinction. Curr. Mol. Med. *16*, 91–102. https://doi.org/10.2174/1566524016666151222150024.

Stem Cell Reports, Volume *17*

Supplemental Information

Modulation by DREADD reveals the therapeutic effect of human iPSC--derived neuronal activity on functional recovery after spinal cord injury

Takahiro Kitagawa, Narihito Nagoshi, Yasuhiro Kamata, Momotaro Kawai, Kentaro Ago, Keita Kajikawa, Reo Shibata, Yuta Sato, Kent Imaizumi, Tomoko Shindo, Munehisa Shinozaki, Jun Kohyama, Shinsuke Shibata, Morio Matsumoto, Masaya Nakamura, and Hideyuki Okano

Supplemental Information

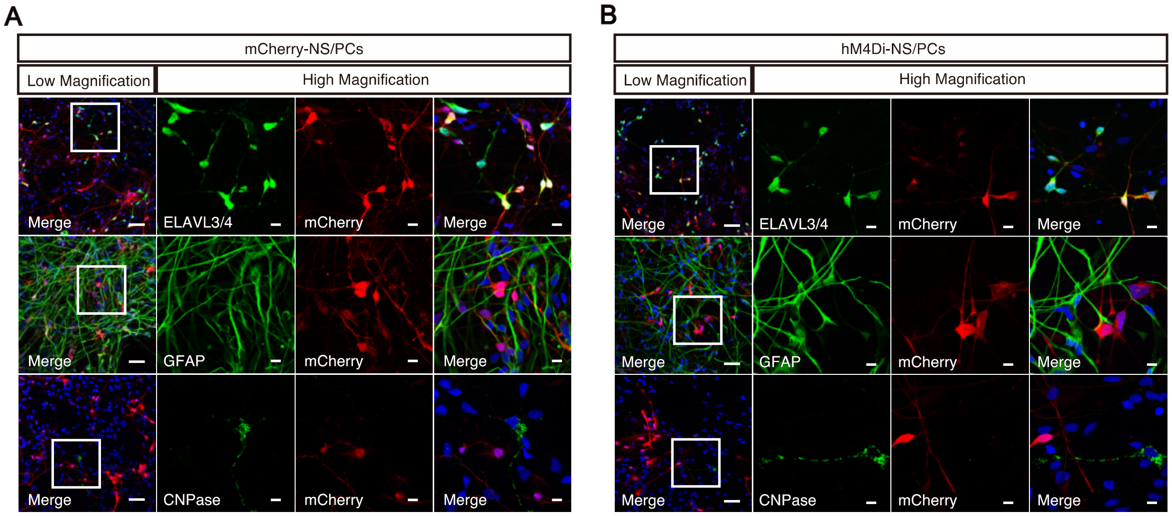

Figure S1. *In vitro* differentiation potential of hM4Di-NS/PCs and mCherry-NS/PCs

(A and B) Representative immunocytochemical images of three neural lineage cell types in mCherry-NS/PCs (A) and hM4Di-NS/PCs (B): ELAVL3/4 (neurons), GFAP (astrocytes), CNPase (oligodendrocytes). Scale bar, 50 μm for low-magnification images and 10 μm for high-magnification images.

ELAVL3/4: embryonic lethal abnormal vision-like protein 3/4, GFAP: glial fibrillary acidic protein, CNPase: cyclic nucleotide phosphodiesterase.

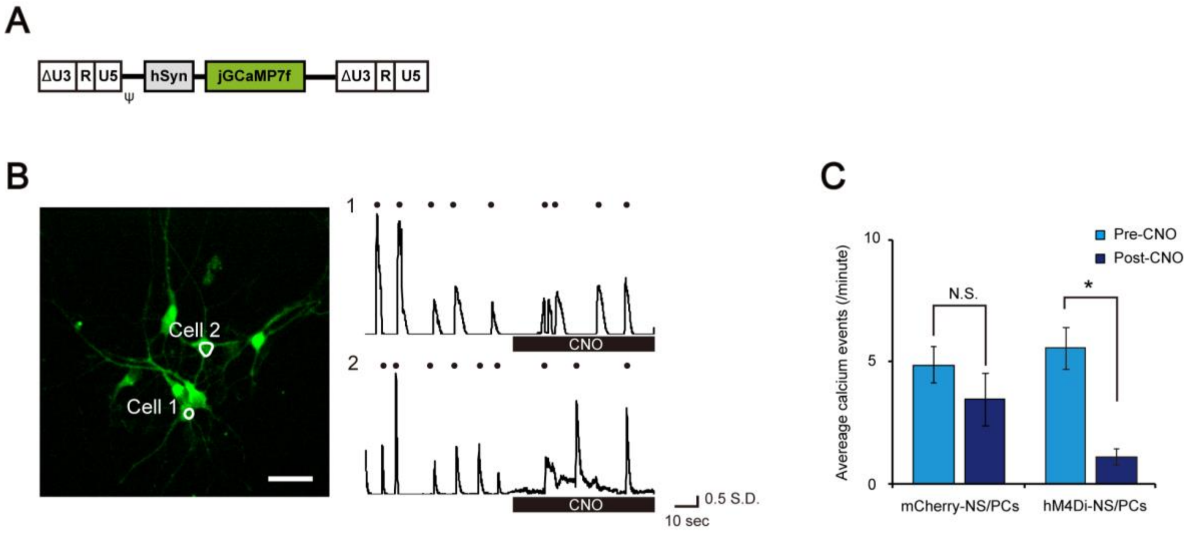

Figure S2. Calcium imaging of neurons differentiated from NS/PCs with CNO administration.

(A) Schematic illustration of the integrated proviral form of the lentiviral vector CSIV-hSyn-jGCaMP7f, which encodes the jGCaMP7f protein under the control of the hSyn promoter.

(B) Representative calcium transients of neurons differentiated from mCherry-NS/PCs. Left: An image of neurons expressing jGCaMP7f in a single field of view. Right: Time traces of representative cells highlighted with white outlines in the left image (the black dot indicates the calcium event). Scale bar, 30 μm.

(C) Average calcium events before and after CNO administration in neurons differentiated from mCherry-NS/PCs and hM4Di-NS/PCs (mCherry-NS/PCs, n = 15 cells / 3 independent experiments, hM4Di-NS/PCs, n = 18 cells / 3 independent experiments).

* $p < 0.05$ and not significant (N.S.) according to the Wilcoxon signed rank test (C). The data are presented as the mean ± SEM (C).

NS/PCs: neural stem/progenitor cells, CNO: clozapine N-oxide, hSyn: human synapsin

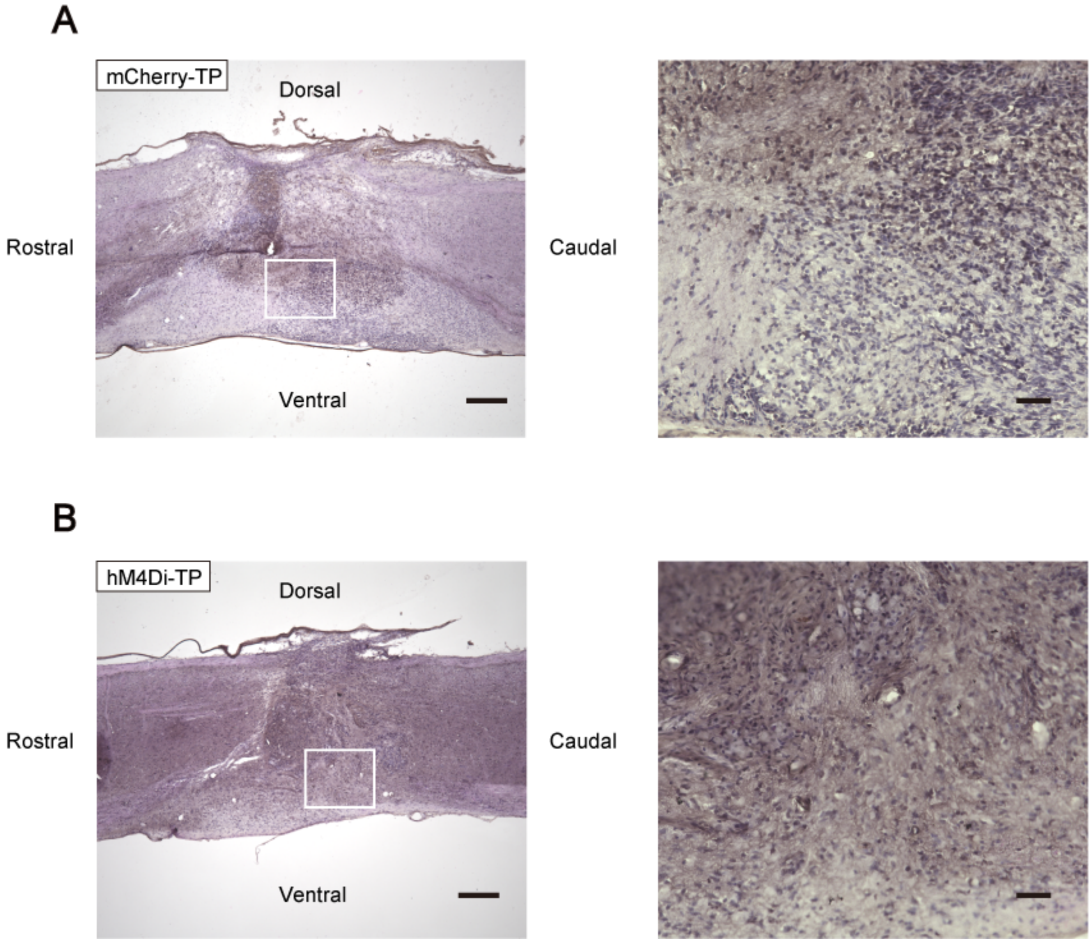

Figure S3. H&E staining of a spinal cord sections

(A and B) Representative images of H&E stained sections. No nuclear atypia nor rosette formation was observed. A: mCherry-TP group, B: hM4Di-TP group. Scale bar, 300μm for low-magnification images and 50 μm for high-magnification images.

TP: transplantation, H&E: hematoxylin-eosin

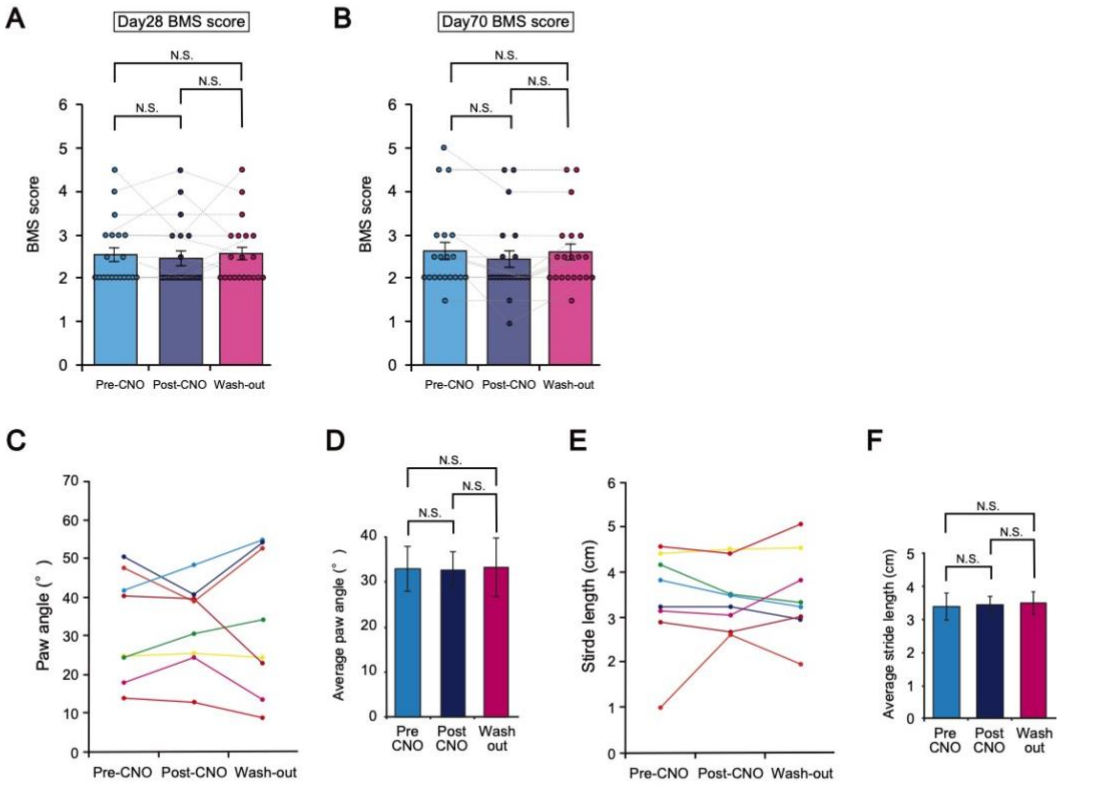

Figure S4. Neuronal activity inhibition assay for PBS group mice

(A and B) BMS scores before CNO administration, after CNO administration and after CNO washout. The scores at 28 days after SCI (A) and 70 days after SCI (B) are presented. (n = 20) (Day28; pre-CNO, 2.6 ± 0.2, post-CNO, 2.5 ± 0.2, wash-out, 2.6 ± 0.2, Friedman test, p = 0.895, day70; pre-CNO, 2.6 ± 0.2, post-CNO, 2.4 ± 0.2, wash-out, 2.6 ± 0.2, Friedman test, p = 0.016, *post hoc* pre-CNO vs. post-CNO, adjusted p = 0.114, pre-CNO vs. wash-out, adjusted p = 1.000, post-CNO vs. wash-out, adjusted p = 0.114).

(C) Raw data of paw angles before CNO administration, after CNO administration and after CNO wash-out for PBS-mice (n = 8).

(D) Quantification of paw angles before CNO administration, after CNO administration and after CNO wash-out for PBS mice (n = 8, pre-CNO, 32.9 ± 5.0°, post-CNO, 32.8 ± 4.0°, wash-out, 33.4 ± 6.6°, Friedman test, p = 1.000).

(E) Raw stride length data before CNO administration, after CNO administration and after CNO wash-out for PBS-mice (n = 8).

(F) Quantification of stride length before CNO administration, after CNO administration and after CNO wash-out for PBS-mice (n = 8, pre-CNO, 3.4 ± 0.4cm, post-CNO, 3.4 ± 0.3cm, wash-out, 3.5 ± 0.3cm, Friedman test, $p = 0.687$).

Not significant (N.S.) according to the Wilcoxon signed rank test following Friedmann test(A, B, D and F). The data are presented as the mean ± SEM (A, B, D and F).

TP: transplantation, BMS: Basso Mouse Scale, CNO: clozapine N-oxide.

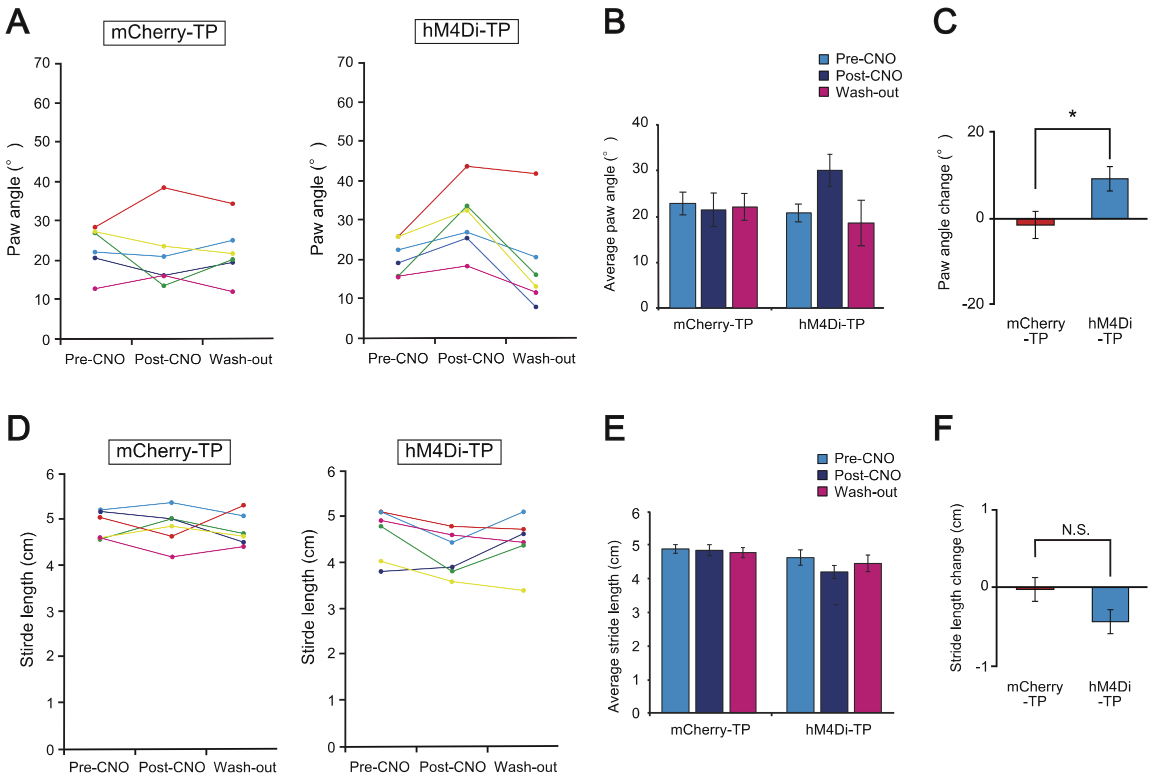

Figure S5. Treadmill analysis data in long-term followed animals

(A) Raw data of paw angles before CNO administration, after CNO administration and after CNO wash-out for mCherry-TP mice (left) and hM4Di-TP mice (right).

(B) Quantification of paw angles before CNO administration, after CNO administration and after CNO wash-out (mCherry-TP group, n = 6, hM4Di-TP group, n = 6, mCherry-TP: pre-CNO, 23.1 ± 2.4°, post-CNO, 21.5 ± 3.7°, wash-out, 22.3 ± 3.0°, Friedman test, $p = 0.607$, hM4Di-TP: pre-CNO, 20.9 ± 1.9°, post-CNO, 30.1 ± 3.5°, wash-out, 18.7 ± 5.0°, Friedman test, $p = 0.009$, *post hoc* pre-CNO vs. post-CNO, adjusted $p = 0.084$, pre-CNO vs. wash-out, adjusted $p = 1.000$, post-CNO vs. wash-out, adjusted $p = 0.084$).

(C) Quantification of the paw angle changes before and after CNO administration in each animal (mCherry-TP group, n = 6, hM4Di-TP group, n = 6, mCherry-TP, -1.6 ± 3.2 vs. hM4Di-TP, 9.2 ± 2.8, $p = 0.041$).

(D) Raw stride length data before CNO administration, after CNO administration and after CNO wash-out for mCherry-TP mice (left) and hM4Di-TP mice (right).

(E) Quantification of stride length before CNO administration, after CNO administration and after CNO wash-out (mCherry-TP group, n = 6, hM4Di-TP group, n = 6, mCherry-TP: pre-CNO, 4.9 ± 0.1cm, post-CNO, 4.9 ± 0.2cm, wash-out, 4.8 ± 0.1cm, Friedman test, $p = 0.846$, hM4Di-TP: pre-CNO, 4.6 ± 0.2cm, post-CNO, 4.2 ± 0.2cm, wash-out, 4.5 ± 0.2cm, Friedman test, $p = 0.135$).

(F) Quantification of the stride length changes before and after CNO administration in each animal (mCherry-TP group, n = 6, hM4Di-TP group, n = 6, mCherry-TP, 0.0 ± 0.2cm vs. hM4Di-TP, -0.4 ± 0.1cm, $p = 0.093$).

* $p < 0.05$ according to the Wilcoxon signed rank test following Friedman test (B and E) and the Mann-Whitney U test (C and F). The data are presented as mean ± SEM. All multiple testing data analyses were followed by Bonferroni correction.

TP: transplantation, BMS: Basso Mouse Scale, CNO: clozapine N-oxide.

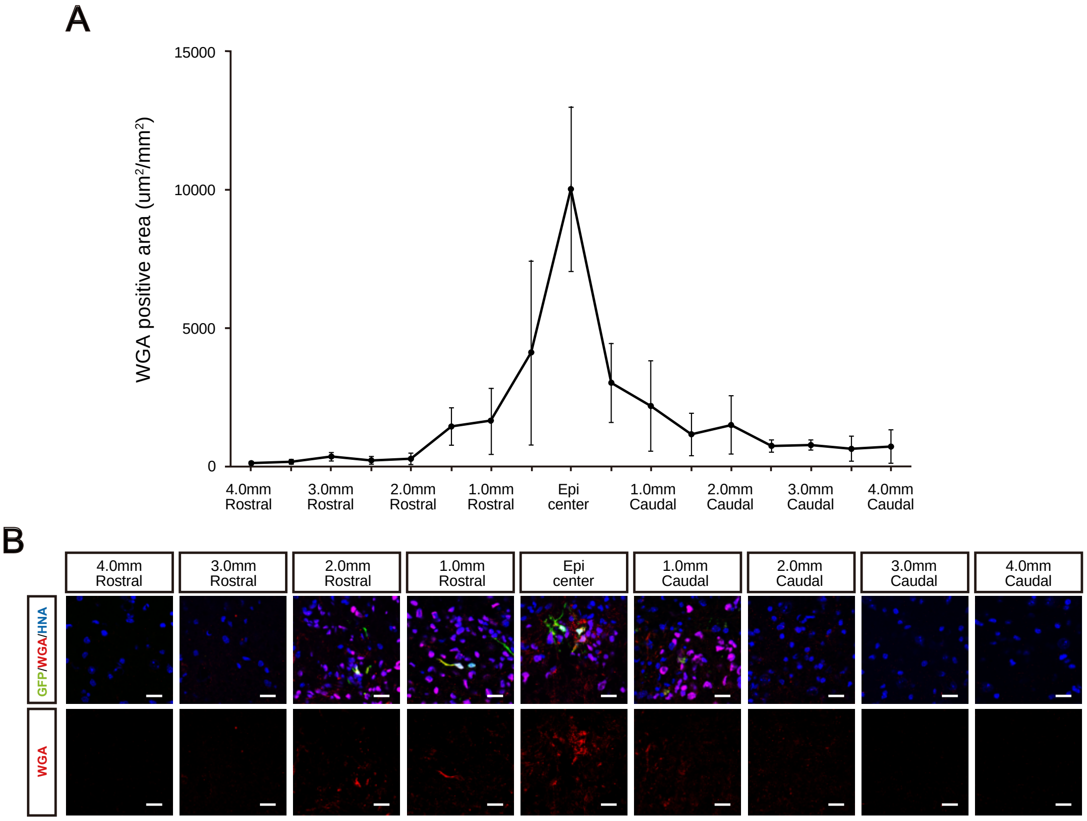

Figure S6. Migrated WGA in the injured spinal cord

(A) Quantitative data of WGA^+/GFP^- areas in different regions (n = 3).

(B) Representative image of migrated WGA at each region. Scale bar, 20 μm.

The data are presented as the mean ± SEM (A).

WGA: Wheat germ agglutinin.

Supplemental Experimental Procedures

Lentiviral vector preparation

Human Synapsin (hSyn), hM4Di and mCherry cDNAs were amplified from pAAV-hSyn-hM4Di-mCherry (addgene #50475) by the polymerase chain reaction (PCR) (1). jGCaMP7f cDNA was amplified from pGP-CMV-jGCaMP7f (addgene #104483). AcGFP1-P2A-WGA cDNA was PCR-amplified from a vector kindly provided by Dr. Yasushi Miyashita (The University of Tokyo, Tokyo, Japan). All cDNAs were transferred into lentiviral vector plasmids: AcGFP1-P2A-WGA cDNA was transferred into the tetracycline(Tet)-inducible lentiviral vector CSIV-TRE-RfA-EF1-Bsd(t), other cDNAs were transferred into the lentiviral vectors CSIV-RfA and CSII-RfA. Recombinant lentiviral vector production was performed as described previously (2). Titer determination was performed by a qRT-PCR based lentiviral titer assay according to the manufacturer's instructions (631235, Cellartis-Takara Bio, Shiga, Japan).

Cell culture and lentiviral transduction

Neural stem/progenitor cells (NS/PCs) were derived as previously reported from a non-tumorigenic clone 414C2 human induced pluripotent stem cells (hiPSCs), that was reprogrammed with episomal plasmid vectors containing six factors (Oct3/4, Sox2, Klf4, L-Myc, LIN28, and p53shRNA) (3-5). The hiPSCs were cultured for 12 days on adhesion culture with mouse embryonic fibroblasts (MEFs). Then, the collected hiPSCs were formed into embryo bodies in floating culture for 30 days. Aggregated cells were dissociated and differentiated into NS/PCs in KBM neural stem cell medium (16050100, Kohjin Bio, Saitama, Japan) containing 2% NeuroBrew-21 (130-093-566, Miltenyi Biotec, North Rhine-Westphalia, Germany), 20 ng/ml human fibroblast growth factor (FGF)-basic (100-18B, Peprotech, Cranbury, NJ, USA) and 10 ng/ml human recombinant leukemia inhibitory factor (hLIF: LIF-1010, Merck Millipore, Burlington, MA, USA). NS/PCs were cultured for 10 to 14 days to allow sphere formation (primary neurospheres). After two passages, the lentiviral vectors were transduced and again cultured as tertiary neurospheres (LV/hSyn-hM4Di-mCherry: hM4Di-NS/PCs, LV/hSyn-mCherry: mCherry-NS/PCs and LV/TRE-AcGFP1-P2A-WGA-EF1-Bsd(t): WGA-NS/PCs).

Neuronal differentiation analysis (*in vitro*)

Dissociated NS/PCs from forth neurospheres were plated onto poly-D lysin/laminin coated 8-well

chamber slides at a density of 1×10^5 cells/well. The cells were cultured in KBM neural stem cell medium containing 2% NeuroBrew-21 at 37°C in 5% CO2 and 95% air for 30 days. Differentiated cells were fixed with 4% paraformaldehyde (PFA) in 0.1 M PBS and stained with the following primary antibodies for immunocytochemical staining: rabbit anti-mCherry (ab167453, Abcam, Cambridge, MA, USA, 1:1000), mouse anti-embryonic lethal abnormal vision-like protein 3/4 (ELAVL 3/4: A21271, Thermo Fisher Scientific, Waltham, MA, USA, 1:200), rat anti-glial fibrillary acidic protein (GFAP: 13-0300, Thermo Fisher Scientific, Waltham, MA, USA, 1:100) and anti-cyclic nucleotide phosphodiesterase (CNPase: C5922, Sigma-Aldrich, St.Louis, MO, USA, 1:2000). Nuclei were stained with Hoechst 33258 (Sigma-Aldrich, St.Louis, MO, USA, 10μg/ml). All *in vitro* images were obtained by confocal laser scanning microscopy (LSM 700, Carl Zeiss, Jena, Germany). Analyses were performed semiautomatically with Zen 2012 SP5 (version 14.0.0.0, Carl Zeiss, Jena, Germany) or a customized macro using ImageJ software(ver. 2.1.0/1.53.c).

Calcium imaging analysis

Tertiary neurospheres of hM4Di-NS/PCs and mCherry-NS/PCs were dissociated and LV/hSyn-jGCaMP7f was transduced. The cells were cultured again as forth neurospheres.

Astrocytes were mechanically dissociated from the cerebral cortices of 3 days old mice (C57B6J, Charles river laboratories Japan, Kanagawa, Japan) and plated on the poly-D lysin coated glass wells. Astrocytes were cultured as feeder cells for 3-7 days at 37°C in 5% CO2 and 95% air. Forth neurospheres of hM4Di-NS/PCs and mCherry-NS/PCs were then dissociated, plated at a density of 2×10^5 cells/well and cultured in Neurobasal Plus Medium (A35829-01, Thermo Fisher Scientific, Waltham, MA, USA) containing: 10 μg/ml Recombinant human brain-derived neurotrophic factor (BDNF: B-250, Alamone labs, Jerusalem, Israel), 10 μg/ml recombinant human glial cell line-derived neurotrophic factor (GDNF: G-240, Alamone labs, Jerusalem, Israel), 100 mM dibutyryladenosine cyclic monophosphate sodium salt (dbcAMP: D0627, Sigma-Aldrich, St.Louis, MO, USA), 200 mM L-ascorbic acid (A8960, Sigma-Aldrich, St.Louis, MO, USA) and 2% B-27 Plus Supplement (A3582801, Thermo Fisher Scientific, Waltham, MA, USA). After culture for 30 days, the cell culture medium was replaced with Hanks' balanced salt solution (HBSS: 14025092, Thermo Fisher Scientific, Waltham, MA, USA) for calcium imaging analysis. Clozapine N-oxide (CNO: BML-NS-105-0025, Enzo Life Sciences, Farmingdale, NY, USA, 10μM of final concentration) was added after 60 seconds of

imaging (total of 120 seconds of observation). Movies were captured at 32 frames/second using an IX83 inverted microscope (Olympus, Tokyo, Japan) equipped with an electron multiplying CCD camera (Hamamatsu Photonics, Shizuoka, Japan) and a pE-4000 LED illumination system (CoolLED, Andover, UK). Data were corrected and analyzed by the miniscope 1-photon-based calcium imaging signal extraction pipeline (6), followed by manual annotation based on identified ROIs and signals using a dedicated GUI written in MATLAB (MathWorks, Inc., MA, USA). Calcium events of detected neurons were identified as the beginning of the rising phase of $\Delta F/F0$ as previously reported (peak $\Delta F/F0>0.5$ standard deviation units from the baseline, derivation of $\Delta F/F0>2$ standard deviation units from the baseline) (7). Normalized calcium event counts after CNO administration for each cell were calculated as follows: Normalized calcium events after CNO administration = calcium events after CNO administration/calcium events before CNO administration (%).

Micro-electrode array

Astrocyte feeder cells were plated on the poly-D lysin coated micro-electrode array (MEA) plates (M768-tMEA-48W, Axion Biosystems, Atlanta, GA, USA). Feeder cells were cultured for 3-7 days at 37°C in 5% CO2 and 95% air. Forth neurospheres of hM4Di-NS/PCs and mCherry-NS/PCs were dissociated, plated at a density of 1×10^5 cells/well and cultured in Neurobasal Plus Medium containing: 10 μg/ml recombinant human BDNF, 10 μg/ml recombinant human GDNF, 100 mM dbcAMP, 200 mM L-ascorbic acid and 2% B-27 Plus Supplement.

All channels were sampled at a rate of 12.5 kHz/channel and filtered using a 200-3000 Hz Butterworth bandpass filter. CNO (10 μM of final concentration) was added after 150 seconds (total of 300 seconds of observation). Spikes were detected at a threshold of 6×SD from the baseline electrode noise. Spikes were counted using the Axion Integrated Studio program (Axion Biosystems, Atlanta, GA, USA). The normalized spike count after CNO administration for each well was calculated as follows: Normalized spike count after CNO administration = spike counts after CNO administration/spike counts before CNO administration (%).

SCI animal model and NS/PC transplantation

Eight weeks old female non-obese diabetic severe combined immunodeficient mice (NOD-SCID, Charles river laboratories Japan, Kanagawa, Japan) were anesthetized with an intraperitoneal

injection of ketamine (100 mg/kg) and xylazine(10 mg/kg). Before making an incision, 12.5 mg/kg ampicillin was administered subcutaneously. Laminectomy was then performed by removing the 10th thoracic spinal vertebral laminae, and the dorsal surface of the dura mater was exposed. Contusive SCI was induced at the 10th level by an Infinite Horizon impactor (70-75 kdyn; Precision Systems and Instrumentation, Lexington, KY, USA), as described previously (8, 9).

7 days after SCI, motor function was evaluated by Basso Mouse Scale (BMS) score. Scores of 2.5 or higher and 1 or lower were excluded from the study. Mice for the neuronal activity inhibition assay (58 mice total) were randomly assigned to one of three groups (hM4Di-TP group, n = 20; mCherry-TP, group n = 18; PBS group, n = 20). Additionally, 18 mice were prepared for the long-term follow up experiment and randomly assigned to two groups (hM4Di-TP group, n = 10; mCherry-TP, group n = 8). 10 mice were prepared for the trans-synaptic tracing assay.

For mice included in the transplantation group, NS/PCs cultured with a small-molecule gamma secretase inhibitor, N-[N-(3,5-difluorophenacetyl)-l-alanyl]-S-phenylglycine t-butyl ester (DAPT: D5942, Sigma-Aldrich, St.Louis, MO, USA, 10 μM) (8), for one day were transplanted into the lesion epicenter of the injury site (5×10^5 cells in 2 μl of PBS, hM4Di-TP group: hM4Di-NS/PCs, mCherry-TP group: mCherry-NS/PCs, trans-synaptic tracing assay group: WGA-NS/PCs) with a Hamilton syringe (87931, Hamilton, Reno, NV, USA) and a 28 G metal needle using a microstereotaxix injection system (KDS310, Muromachi-Kikai Co., Ltd., Tokyo, JAPAN). NS/PCs were injected at a rate of 1 μl/minute, and the syringe was left at the injection site for two minutes after the injection before being removed. Using the same method, 2 μl of PBS was injected into the lesion epicenter of the injury site in PBS group mice.

All experiments were performed in accordance with the Guidelines for the Care and Use of Laboratory Animals of Keio University (Tokyo, Japan, Permit Number; 13020) and NIH Guide for the Care and Use of Laboratory Animals. All surgeries were performed under anesthesia.

Histological analyses

Ten weeks after transplantation, animals were anesthetized and euthanized by transcardial perfusion with 0.1 M PBS containing 4% PFA, followed by sequential soaking overnight in 10% and 30% sucrose. Spinal cord tissues were the embedded in optimal cutting temrerture (O.C.T.) compound (Sakura Finetechnical Co., Ltd., Tokyo, Japan) and sectioned at a thickness of 16 mm for the sagittal

plane and 20 mm for the axial plane.

Spinal cord sections were immunohistochemically stained for histological analysis using the following primary antibodies; mouse anti-human nuclear antigen (HNA: MAB4383, Merck Millipore, Burlington, MA, USA, 1:400), rabbit anti-mCherry (ab167453, 1:1000), mouse anti-ELAVL 3/4 (A21271, 1:200), rabbit anti-GFAP (16825-1-AP, Proteintech, Rosemont, IL, USA, 1:4000), mouse anti-adenomatous poluposis coli CC-1 (APC: OP80, Merck Millipore, Burlington, MA, USA, 1:400), rabbit anti-Nestin (18741, Immuno-Biological Laboratories, Gunma, Japan, 1:400), rabbit anit-Ki67 (NCL-Ki67p, Leica Biosystems, Buffalo Grove, IL, USA, 1:2000), mouse anti-synaptophysin (MAB329, Merck Millipore, Burlington, MA, USA, 1:10000), mouse anti-Gephyrin(1470011, Synaptic Systems, Goettingen, Germany, 1:1000), anti-post synaptic density 95 (PSD95: 51-6900, Thermo Fisher Scientific, Waltham, MA, USA, 1:100), mouse anti-human cytoplasm antibody (STEM121: Y40420, Cellartis-Takara Bio, Shiga, Japan, 1:200), rabbit anti-wheat germ agglutinin (WGA: T4144, Thermo Fisher Scientific, Waltham, MA, USA, 1:20000), goat anti-GFP (600-101-215, Rockland Immunochemicals, Pottstown, PA, USA, 1:1000), goat anti-choline acetyltransferase antibody (ChAT: AB144P, Merck Millipore, Burlington, MA, USA, 1:200). Nuclei were stained with Hoechst 33258 (10μg/ml). Sample images were obtained using a fluorescence microscope (BZ 9000, Keyence Co., Osaka, Japan) or a confocal laser scanning microscopy. Analyses were performed in combination with Zen 2012 SP5 and a customized macro using ImageJ software.

Immunoelectron microscopy analysis

Briefly, spinal cord tissues were prepared as mentioned above by perfusion and by postfixation with 4% PFA, followed by the cryoprotective treatment with 15% and 30% sucrose, embedding and freezing into a cryomold with Frozen Section Compound (FSC22, Leica Microsystems, Germany). Frozen sections were prepared at 20 μm thickness with a cryostat (CM3050s, Leica Microsystems, Germany). Sections were incubated with 5% Blockace (DS Pharma Biomedical, Osaka, Japan) supplemented with 0.01% Saponin in 0.1 M PB for an hour. The sections were stained with a rabbit anti-mCherry (ab167453, 1:200) primary antibody for 72 hours at 4°C and incubated with a FluoroNanogold-conjugated goat anti-rabbit secondary antibody (A24922, Thermo Fisher Scientific, Waltham, MA, USA, 1:100) for 24 hours at 4°C. After 2.5% glutaraldehyde fixation, nanogold signals were enhanced with Silver Enhancement solution for seven minutes at 25°C. The sections were

postfixed with 1.0% Osmium Tetroxide for 90 minutes at 4°C, *en bloc* stained with uranyl acetate for 20 minutes at 4°C, dehydrated through a graded ethanol series, and embedded into pure Epon. Ultrathin sections (80 nm) were prepared with an ultramicrotome (UC7, Leica Microsystems, Germany) and electron stained with uranyl acetate and lead citrate. Ultrathin sections were finally observed under a transmission electron microscope (JEM1400 plus, JEOL, Tokyo, Japan).

Behavioral analyses

Investigators blinded to identify the experimental groups performed this assessment. Hind limb locomotor function was evaluated using the BMS scoring system (10) on a weekly basis. The gait analyses were performed on a treadmill (DigiGait system, Mouse Specifics, Framingham, MA, USA) at the final follow-up point. The stride length and stance angle were determined on a treadmill set to a speed of 7 cm/second (8).

Neural activity inhibiting assay (*in vivo*)

BMS scores were evaluated as a baseline function (8-10). CNO (10mg/kg) dissolved in saline was intraperitoneally administered to all animals (11, 12). Functional analyses were performed again at 1 and 24 hours after CNO administration. BMS scoring of mice with neuronal activity inhibition was performed at 28 days after SCI and at the final follow-up. Treadmill gait analysis of mice with neural activity inhibition was performed at the final follow-up for mice with BMS scores of three or more on either side of the hind limb in the hM4Di-TP and mCherry-TP groups. The administration of CNO and the functional analysis were performed at time points similar to those used for BMS scoring.

Electrophysiology

Electrophysiological experiments were performed using a Neuropack S1 MEB9402 signal processor (Nihon Kohden, Tokyo, Japan) at 18 weeks post injury as previously described (13, 14). Mice were anesthetized by intraperitoneal injections of three types mixed anesthesia (5 mg/kg butorphanol, 0.75 mg/kg medetomidine, and 4 mg/kg midazolam). The spinal cord at the Th5 level was exposed and stimulated by a nichrome wire tied to artificial dura mater that was placed on the exposed spinal cord. The signal in the hindlimb was detected by wire electrodes (gage 0.0508 mm, nichrome). The active electrode was placed in the quadriceps muscle belly, the reference electrode was placed near the

distal quadriceps tendon of the muscle, and the ground electrode was placed in the tail. Stimulation with an intensity of 1.0–3.0 mA, 1-3 train, duration of 0.2 ms, and interstimulus interval of 1 Hz was used. An average of 20 MEP wave was aggregated as a representative wave. The maximal amplitude was measured from the lowest point to the highest point of the wave. Peak latency was measured as the length of time from the stimulation to the highest point of the MEP wave. After the first evaluation, 10mg/kg CNO was administered under anesthesia. MEP wave was re-evaluated 30 minutes after CNO administration. Post-CNO measurements were normalized as follows: Normalized measurement = post-CNO measurement / pre-CNO measurement.

Induction of doxycycline

Mice allocated to the trans-synaptic tracing assay group were fed an autoclaved rodent diet supplemented with 0.0625% doxycycline(DOX: D13110903, Research Diets, New Brunswick, NJ, USA) beginning at 56 days after SCI for a period of 14 days (15).

Supplemental References

1. Roth BL. DREADDs for Neuroscientists. Neuron. 2016;89(4):683-94.

2. Miyoshi H, Blomer U, Takahashi M, Gage FH, Verma IM. Development of a self-inactivating lentivirus vector. J Virol. 1998;72(10):8150-7.

3. Okada Y, Matsumoto A, Shimazaki T, Enoki R, Koizumi A, Ishii S, et al. Spatiotemporal recapitulation of central nervous system development by murine embryonic stem cell-derived neural stem/progenitor cells. Stem Cells. 2008;26(12):3086-98.

4. Nori S, Okada Y, Yasuda A, Tsuji O, Takahashi Y, Kobayashi Y, et al. Grafted human-induced pluripotent stem-cell-derived neurospheres promote motor functional recovery after spinal cord injury in mice. Proc Natl Acad Sci U S A. 2011;108(40):16825-30.

5. Okubo T, Nagoshi N, Kohyama J, Tsuji O, Shinozaki M, Shibata S, et al. Treatment with a Gamma-Secretase Inhibitor Promotes Functional Recovery in Human iPSC- Derived Transplants for Chronic Spinal Cord Injury. Stem Cell Reports. 2018;11(6):1416-32.

6. Lu J, Li C, Singh-Alvarado J, Zhou ZC, Frohlich F, Mooney R, et al. MIN1PIPE: A Miniscope 1-Photon-Based Calcium Imaging Signal Extraction Pipeline. Cell Rep. 2018;23(12):3673-84.

7. Nemoto A, Kobayashi R, Yoshimatsu S, Sato Y, Kondo T, Yoo AS, et al. Direct Neuronal

Reprogramming of Common Marmoset Fibroblasts by ASCL1, microRNA-9/9*, and microRNA-124 Overexpression. Cells. 2020;10(1).

8. Okubo T, Iwanami A, Kohyama J, Itakura G, Kawabata S, Nishiyama Y, et al. Pretreatment with a gamma-Secretase Inhibitor Prevents Tumor-like Overgrowth in Human iPSC-Derived Transplants for Spinal Cord Injury. Stem Cell Reports. 2016;7(4):649-63.

9. Kamata Y, Isoda M, Sanosaka T, Shibata R, Ito S, Okubo T, et al. A robust culture system to generate neural progenitors with gliogenic competence from clinically relevant induced pluripotent stem cells for treatment of spinal cord injury. Stem Cells Transl Med. 2021;10(3):398-413.

10. Basso DM, Fisher LC, Anderson AJ, Jakeman LB, McTigue DM, Popovich PG. Basso Mouse Scale for locomotion detects differences in recovery after spinal cord injury in five common mouse strains. J Neurotrauma. 2006;23(5):635-59.

11. Dell'Anno MT, Wang X, Onorati M, Li M, Talpo F, Sekine Y, et al. Human neuroepithelial stem cell regional specificity enables spinal cord repair through a relay circuit. Nat Commun. 2018;9(1):3419.

12. Jendryka M, Palchaudhuri M, Ursu D, van der Veen B, Liss B, Katzel D, et al. Pharmacokinetic and pharmacodynamic actions of clozapine-N-oxide, clozapine, and compound 21 in DREADD-based chemogenetics in mice. Sci Rep. 2019;9(1):4522.

13. Tashiro S, Nishimura S, Iwai H, Sugai K, Zhang L, Shinozaki M, et al. Functional Recovery from Neural Stem/Progenitor Cell Transplantation Combined with Treadmill Training in Mice with Chronic Spinal Cord Injury. Sci Rep. 2016;6:30898.

14. Ito S, Nagoshi N, Tsuji O, Shibata S, Shinozaki M, Kawabata S, et al. LOTUS Inhibits Neuronal Apoptosis and Promotes Tract Regeneration in Contusive Spinal Cord Injury Model Mice. eNeuro. 2018;5(5).

15. Kojima K, Miyoshi H, Nagoshi N, Kohyama J, Itakura G, Kawabata S, et al. Selective Ablation of Tumorigenic Cells Following Human Induced Pluripotent Stem Cell-Derived Neural Stem/Progenitor Cell Transplantation in Spinal Cord Injury. Stem Cells Transl Med. 2019;8(3):260-70.
